# Nucleophilic Addition of Stabilized Phosphorus Ylides to *Closo*-Decaborate Nitrilium Salts: A Synthetic Route to Boron Cluster-Functionalized Iminoacyl Phosphoranes and Their Application in Potentiometric Sensing

**DOI:** 10.3390/molecules31020231

**Published:** 2026-01-09

**Authors:** Vera V. Voinova, Eugeniy S. Turyshev, Sergey S. Novikov, Nikita A. Selivanov, Alexander Yu. Bykov, Ilya N. Klyukin, Andrey P. Zhdanov, Mikhail S. Grigoriev, Konstantin Yu. Zhizhin, Nikolay T. Kuznetsov

**Affiliations:** 1 N.S. Kurnakov Institute of General and Inorganic Chemistry of the Russian Academy of Sciences, Leninskii pr.31, 119991 Moscow, Russia; veravoinova@rx24.ru (V.V.V.); exsergion@gmail.com (S.S.N.); goovee@yandex.ru (N.A.S.); bykov@igic.ras.ru (A.Y.B.); klukinil@gmail.com (I.N.K.); zhizhin@igic.ras.ru (K.Y.Z.); ntkuz@igic.ras.ru (N.T.K.); 2 Institute for African Studies of the Russian Academy of Sciences (IAS), st. Spiridonovka 30/1, 123001 Moscow, Russia; tyrishev@gmail.com; 3 Faculty of Fundamental Physical and Chemical Engineering, Lomonosov Moscow State University, Leninskie Gory 1, 119991 Moscow, Russia; 4 Frumkin Institute of Physical Chemistry and Electrochemistry, Russian Academy of Sciences, Leninskii pr. 31, Bldg 4, 119071 Moscow, Russia; mickgrig@mail.ru

**Keywords:** *closo*-decaborate, phosphorus ylides, nucleophilic addition, ion-selective electrodes

## Abstract

This work explores a novel and efficient synthetic approach to a new class of boron cluster derivatives via the nucleophilic addition of stabilized phosphorus ylides, Ph_3_P=CHR^2^ (R^2^ = COOEt, CN), to a series of nitrilium salts of the *closo*-decaborate anion, [2-B_10_H_9_NCR^1^]^−^ (R^1^ = Me, Et, *^n^*Pr, *^i^*Pr, Ph). The reaction proceeds regio- and stereospecifically, affording a diverse range of iminoacyl phosphorane derivatives, [2-B_10_H_9_NH=C(R^1^)C(PPh_3_)R^2^]^−^, in high isolated yields (up to 95%). The obtained compounds (10 examples) were isolated as tetrabutylammonium or tetraphenylphosphonium salts and thoroughly characterized by multinuclear NMR (^11^B, ^1^H, ^13^C, ^31^P), high-resolution mass spectrometry, and single-crystal X-ray diffraction. The reaction feasibility was found to be strongly influenced by the steric hindrance of the R^1^ group. Furthermore, the practical utility of these novel hybrids was demonstrated by employing the [2-B_10_H_9_NH=C(CH_3_)C(COOC_2_H_5_)=PPh_3_]^−^ anion as a highly effective membrane-active component in ion-selective electrodes. The developed tetraphenylphosphonium (TPP^+^) sensor exhibited a near-Nernstian response, a low detection limit of 3 × 10^−8^ M, and excellent selectivity over a range of common inorganic and organic cations, showcasing the potential of *closo*-borate-based ionophores in analytical chemistry.

## 1. Introduction

Anionic boron clusters and carboranes have attracted considerable research interest owing to their broad applicability across various scientific and technological domains. These compounds are integral to the development of antiviral and antimicrobial agents [1,2,3,4], materials with tailored magnetic and optical properties, catalytic systems [5,6], and energy conversion devices [7,8,9]. Recently, the utilization of boron clusters in Boron Neutron Capture Therapy (BNCT) has gained substantial momentum, driven by the emergence of new stable accelerator-based neutron sources [10,11,12,13].

The development of effective pharmaceutical agents and materials with predefined properties necessitates robust synthetic methodologies for substituted *closo*-borate anions that permit the introduction of diverse functional groups. The three-dimensional aromaticity of boron clusters enables hydrogen substitution via electrophilic, radical, and nucleophilic pathways. Nevertheless, the most promising strategies for directed functionalization involve the modification of pre-introduced *exo*-polyhedral groups. These include nucleophilic ring-opening of cyclic substituents [14,15], electrophilic alkylation of sulfo [16,17,18] and amino [19,20] groups, and *ipso*-substitution reactions [21,22,23].

Nucleophilic addition reactions to nitrilium substituents are of particular significance, as they provide a versatile route for incorporating a wide array of functional moieties, including those suitable for constructing complex biomimetic systems [24,25,26,27,28]. To date, the literature reports only two examples of C-nucleophile addition to nitrilium derivatives of *closo*-borates: reactions with azomethine ylides [29] and compounds bearing an activated methylene group [30].

Stabilized phosphorus ylides constitute a promising class of reagents in the context of functionalization. These versatile 1,2-dipolar compounds feature a negative charge localized on the carbon atom, stabilized by an adjacent phosphonium group, which imparts unique reactivity [31]. Carbonyl-stabilized phosphorus ylides have been successfully employed in cyclization reactions to facilitate the efficient synthesis of 1-fluoroalkyl-5-substituted-1,2,3-triazoles, which represent valuable structural motifs in medicinal chemistry [32]. Furthermore, polymers derived from phosphorus ylides exhibit promising antifouling properties and selective bactericidal activity, rendering them attractive candidates for biomedical applications [33]. The chemical diversity inherent in these compounds opens new avenues for catalyst design and precision molecular engineering [34].

A critical aspect of employing modified boron clusters lies in the development of analytical systems for environmental monitoring. The control of organic pollutants in industrial and agricultural effluents represents a pressing global challenge. *Closo*-decaborate derivatives are employed as active components in potentiometric sensor membranes designed for the detection of substances such as lidocaine, procaine, articaine, terbinafine, and quaternary ammonium compounds. Advances in synthetic methodologies enable the production of derivatives bearing novel functional groups, thereby expanding the capabilities for identifying challenging-to-detect organic compounds in complex matrices [35,36].

In the present study, we expand the range of C-nucleophilic reagents employed and provide a detailed examination of the nucleophilic addition of stabilized phosphorus ylides to nitrilium derivatives of *closo*-decaborate anion. Additionally, this work describes the synthesis of the compound TPP[B_10_H_9_NH=C(CH_3_)C(COOC_2_H_5_)=PPh_3_] and investigates its potential application as a membrane-active component in a potentiometric sensor for the direct determination of the tetraphenylphosphonium cation.

## 2. Results

### 2.1. Synthesis and Characterization of Borylated Iminoacyl Phosphoranes

**Figure 1:**
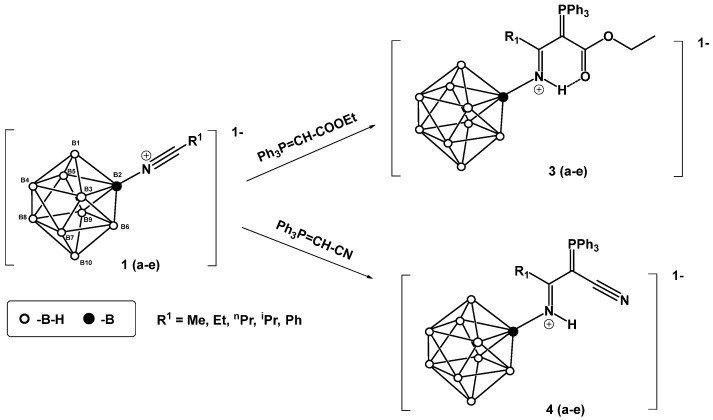
Reaction scheme of iminoacylation process of stabilized phosphorus ylides.

This work investigates the nucleophilic addition of stabilized phosphorus ylides, as neutral C-nucleophiles Ph_3_P=CH-R^2^ (R^2^ = COOEt, CN), to the activated triple bond of *closo*-decaborate nitrilium derivatives [2-B_10_H_9_NCR^1^]^−^ (R^1^ = Me, Et, *^n^*Pr, *^i^*Pr, *^t^*Bu, Ph) (Figure 1). The reaction proceeds efficiently and selectively to yield the corresponding iminoacylation products. It is noteworthy that no byproducts arising from triphenylphosphine group migration, which are common in analogous reactions with platinum nitrile complexes, were observed [37,38]. The reaction feasibility (specifically, the required temperature and reagent excess) was found to be strongly influenced by the steric hindrance of the R^1^ group. While high yields were eventually achieved for most substrates through condition optimization, the reactivity profiles differed significantly. For derivatives with R^1^ = Me, Et, *^n^*Pr, Ph, the reaction proceeded smoothly at room temperature in dichloromethane using an equimolar amount of the ylide. However, the isobutyronitrile-derived substrate (R^1^ = *^i^*Pr) required heating to 70 °C in acetonitrile and a fivefold excess of the phosphorus ylide. No reaction was observed for the *tert*-butyl derivative (R^1^ = *^t^*Bu) even under more forcing conditions. The resulting compounds, [2-B_10_H_9_N=HC(R^1^)(C=PPh_3_R^2^)], can be classified as highly stabilized phosphorus ylides [39,40,41,42,43,44].

All obtained salts (Bu_4_N)[2-B_10_H_9_N=HC(R^1^)(C=PPh_3_R^2^)] were characterized by ^11^B{^1^H}, ^1^H, ^13^C{^1^H}, and ^31^P NMR spectroscopy, as well as HRMS-ESI^−^.

The NMR spectra reveal distinct structural features for the ester-substituted products **3 (a-e)** versus their cyano-analogs **4 (a-e)**. In the ^11^B{^1^H} NMR spectra, both series exhibit similar patterns, though signals for **4 (a-e)** are shifted downfield. For derivatives **3 (a-e)**, signals appear in characteristic ranges: apical B(10) (δ −0.9 to −0.6) and B(1) (δ −3.4 to −2.3), substituted equatorial B(2) (δ −14.2 to −13.9), and two sets of equatorial signals for B(3,5,6,9) and B(4,7,8) between δ −25.4 and −28.3. In contrast, the cyano derivatives **4 (a-e)** display apical signals at δ −1.6 to −0.6 and −3.7 to −3.1, with B(2) at δ −14.3 to −13.8. Notably, the equatorial belt in **4 (a-e)** splits into three distinct signals (δ −25.8 to −25.2, −29.0 to −28.6, and −30.6 to −29.7), reflecting lower symmetry.

The ^1^H NMR spectra highlight a fundamental structural difference: derivatives **3 (a-e)** show a broad NH signal at δ 10.43–10.21, indicative of a strong intramolecular hydrogen bond (C=O … H-N). Conversely, in **4 (a-e)**, the NH signal shifts significantly upfield to δ 6.09–5.73, confirming the absence of such bonding. While the ethoxy group signals in **3** confirm the ester structure, the alkyl/aryl substituents in **4** appear as broadened signals, suggesting greater conformational flexibility.

^13^C{^1^H} NMR analysis further differentiates the series. In **3 (a-e)**, the phosphorylated methine group (P-C-C=O) resonates at δ 63.0–61.2 (^1^*J^C-P^* 124–123 Hz) with carbonyl signals at δ 178.5–169.3. For **4 (a-e)**, the corresponding phosphorylated methine fragment (P-C-C≡N) shifts to δ 46.7–43.1 (^1^*J^C-P^* 120.7–120.0 Hz), with the nitrile carbon appearing as a doublet at δ 123.1–119.9. ^31^P{^1^H} NMR spectra for all compounds show a single signal in the narrow range δ 19.4–16.9, consistent with a single phosphorus environment.

HRMS-ESI^−^ data confirm the composition and structure of the obtained compounds. In the spectra of derivatives **3 (a-e)**, peaks corresponding to molecular ions [A]^−^ are observed, where A = [B_10_H_9_NHC(R^1^)(Ph_3_PCCOOEt)], as well as peaks of aggregated forms, such as {2[A] + Na}^−^ and {[A] + Na + 2HCOO-H}^−^. In the spectra of derivatives **4 (a-e)**, peaks [A]^−^ are observed, where A = [B_10_H_9_NHC(R^1^)(Ph_3_PCCN)].

### 2.2. X-Ray Structure Determination

**Figure 2:**
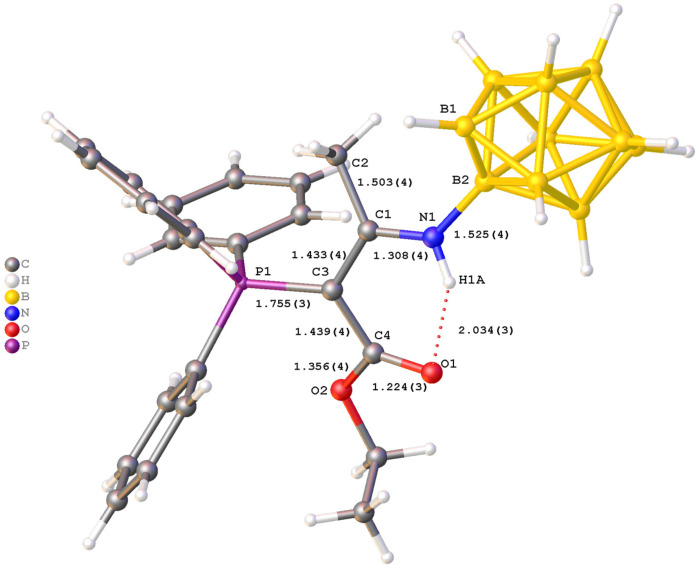
Molecular structure of anion in (Ph_4_P)(**3a**).

The structure of two compounds, (Ph_4_P)(**3a**) and (Bu_4_N)(**4b**), was determined by single-crystal X-ray diffraction. The crystal structure of (Ph_4_P)(**3a**) is built from tetraphenylphosphonium cations and substituted *closo*-decaborate anions. In the molecular structure of (Ph_4_P)(**3a**) (Figure 2), the anion has the geometry of a slightly distorted bicapped Archimedean antiprism. The substituent is in an equatorial position. The N(1)–B(2) bond length is 1.525(4) Å, which is typical for a single B–N(sp^2^) bond. The bond lengths N(1)–C(1) and C(1)–C(3) (1.308(4) and 1.433(4) Å, respectively) have an intermediate order between single and double bonds, although the N(1)–C(1) bond is closer to a double bond, while the C(1)–C(3) bond length corresponds to a single C(sp^2^)–C(sp^2^) bond. A similar structure is found in acylated derivatives [40,42] and adducts of stabilized phosphorus ylides and phenyl isothiocyanate [45].

The carboxylate group shows characteristic bond lengths: C(4)=O(1) 1.224(3) Å (double bond) and C(4)–O(2) 1.356(4) Å (single bond) [46]. The geometry of the N(1)-C(1)-C(3)-P(1) fragment is nearly planar. The torsional angle value is −6.9° (3). All bond lengths and angles in the phenyl substituents at the phosphorus atoms and the cation are typical for aromatic systems. The phosphorus centers have a tetrahedral environment with angles in the range of 102–114°. It is worth noting that the structural rigidity of the substituent is provided by an intramolecular hydrogen bond—N(1)–H(1A)···O(1) with parameters:

H(1A)···O(1) = 2.03 Å;

N(1)···O(1) = 2.718(3) Å;

N(1)–H(1A)···O(1) = 133.8°.

This H-bond forms a six-membered ring between the carboxyl group and the amino group of the imine. The N(1)···O(1) distance is significantly shorter than the sum of the van der Waals radii (~3.07 Å), confirming a substantial interaction energy.

**Figure 3:**
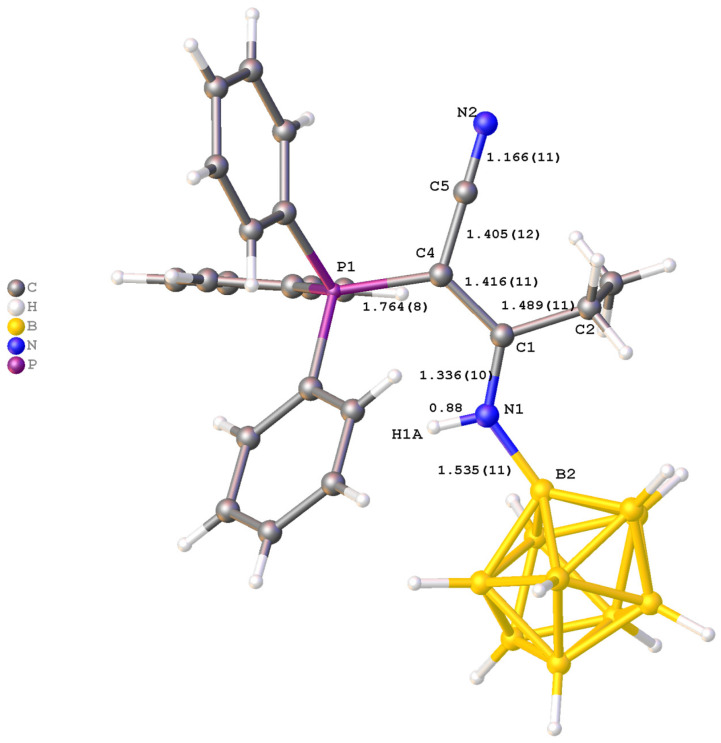
Molecular structure of anion in (Bu_4_N)(**4b**).

In the molecular structure of (Bu_4_N)(**4b**), the substituent in the boron cluster anion is also in an equatorial position (Figure 3). The N(1)–B(2) bond length is 1.535(11) Å, which is typical for a single B–N bond with an sp^2^-hybridized nitrogen atom. The bond lengths N(1)–C(1) and C(1)–C(4) (1.336(10) and 1.416(11) Å, respectively) indicate an intermediate bond order between single and double. The N(1)–C(1) bond demonstrates a pronounced double bond character, while the C(1)–C(4) bond is closer to a single Csp^2^–Csp^2^ bond. The C(4)–C(5) bond length (1.405(12) Å) also has an intermediate character between single and double, while the C(5)–N(2) bond length (1.166(11) Å) corresponds to a triple C≡N bond.

The iminoacylated ylide fragment is almost planar, as evidenced by the torsional angles:

B(2)N(1)C(1)C(4): −177.8(9)°;

N(1)C(1)C(4)C(5): −174.2(9)°;

C(1)C(4)C(5)N(2): 179.3(11)°.

All key atoms N(1), C(1), C(4), C(5), and N(2) are in a *trans*-conformation relative to each other, forming a fully conjugated π-system. The bond lengths and angles in the phenyl substituents at the phosphorus atom P(1) are typical for such structures.

The comparison of **(Ph_4_P)(3a)** and **(Bu_4_N)(4b)** reveals that the spatial arrangement of the ylide substituent is governed by a balance between electronic conjugation and steric requirements. In **(Ph_4_P)(3a)**, the ylide fragment adopts a specific *E*-conformation (relative to the C(1)-C(3) bond), locked by the intramolecular N–H···O hydrogen bond. This conformation creates a planar, six-membered pseudo-cyclic ring that rigidly extends from the boron cage. This rigid planar geometry has significant steric implications. The bulky triphenylphosphonium group is positioned *anti* to the boron cluster relative to the C(1)–C(3) bond axis to minimize steric hindrance with the cage. Consequently, the phosphorus atom and its three phenyl rings effectively shield one face of the ylidic carbon, while the boron cluster shields the “tail” of the substituent. This steric effect restricts rotation around the P–C and C–C bonds, locking the substituent into a fixed orientation that is optimal for π-overlap but effectively inaccessible to external nucleophiles. This steric protection is a key factor in the remarkable hydrolytic and thermal stability of these compounds, preventing the typical degradation pathways (like hydrolysis) often seen in non-stabilized ylides.

The synthesis of these derivatives confirms that the chosen strategy is a powerful method for functionalizing *closo*-decaborate anion. The combination of resonance stabilization, intramolecular H-bonding (locking the conformation), and steric shielding (by PPh_3_ and the boron cage) renders these substituents chemically inert to hydrolysis and oxidation while maintaining specific reactivity for sensing applications.

### 2.3. Potentiometric Response Characteristics of the Sensors

The measurements were conducted using a measuring electrode fitted with a membrane, with both the measuring and reference electrodes simultaneously immersed in the sample solution. The solution was continuously stirred during the measurements. The measurement was continued until a stable reading was obtained on the instrument: 20–40 s for concentrations between 10^−2^ and 10^−5^, and 45–60 s for concentrations between 10^−6^ and 10^−8^. After the measurements, the electrodes were rinsed with deionized water and reused.

The tetraphenylphosphonium (TPP) ion-selective membrane sensors were calibrated, and the potentiometric selectivity coefficients were determined. The potentiometric response characteristics of the sensor were found to be dependent on the amount of the membrane active compound—TPP[B_10_H_9_NH=C(CH_3_)C(COOC_2_H_5_)=PPh_3_] in the membrane composition (Table 1).

**Figure 4:**
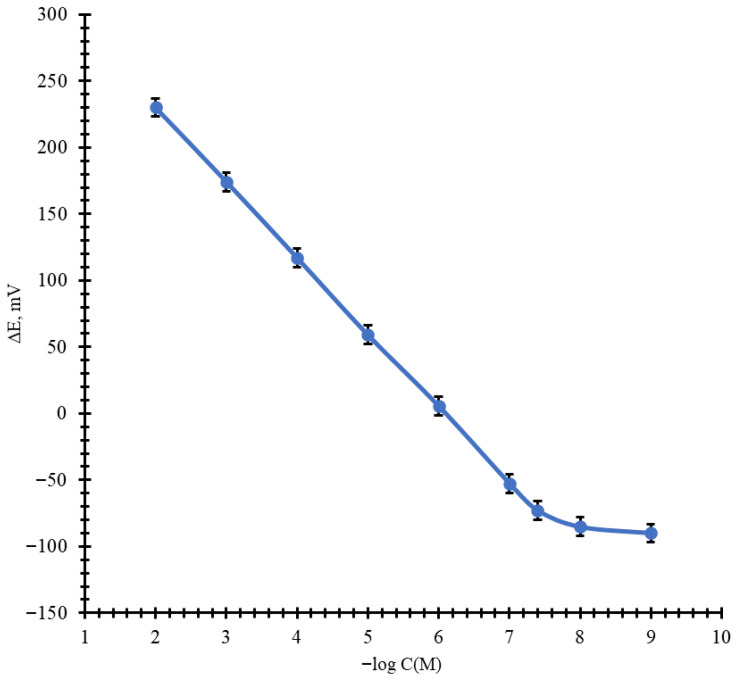
Average potentiometric calibration plot for the TPP-selective sensor (Membrane No. 2).

As follows from the results obtained, the sensor based on the membrane No. 2 (Figure 4) exhibited optimal characteristics. This sensor showed a near-Nernstian response in the concentration range of 1 × 10^−7^–1 × 10^−2^ mol/L in the pH range of 4.0–7.5, and the lower detection limit (LOD) of 3 × 10^−8^ mol/L.

The average calibration curve was constructed from 50 measurements. The measured potential values differ by no more than ±0.3 mV. The potential for concentrations of 10^−2^–10^−5^ stabilizes within 15–30 s, for concentrations of 10^−5^–10^−7^ within 30–60 s. The TPP sensor with membrane No. 2 was studied for 6 months (100 measurements), and the potential drift was no more than ±2 mV. Averaged calibration curves for other compositions are provided in the Supplementary Materials (Figure S53).

The interference of some common cations on the sensor response was studied by the mixed solution method [47]. The potentiometric measurements were carried out using test solutions containing the constant concentration of an interfering cation (0.01 mol/L). The calculated selectivity coefficients values (pK^pot^ _TPP, Cation_) are shown in Table 2. These values clearly indicate that the TPP sensor is fairly selective to TPP cations over the different tested cations. The selectivity coefficients were calculated using 5 measurements. The errors in calculating the selectivity coefficients (pK^pot^ _TPP/Cation_) were no more than ±0.1. A graphical method for determining the selectivity coefficients is provided in the Supplementary Materials (Figures S54–S66).

The interfering ions were chosen to reflect the most common inorganic cations present in environmental and laboratory aqueous media (alkali/alkaline-earth ions, NH_4_^+^, H^+^), and ions of different charge that may alter membrane ion-exchange equilibria. Bu_4_N^+^ was included as a representative lipophilic organic monovalent cation to evaluate competitive extraction/ion pairing with the target TPP^+^.

Selective determination of TPP in the presence of TBA can be quite challenging because both cations are lipophilic, organic, and singly charged. However, tetraphenylphosphonium is frequently used as an analytical reagent in extractive spectrophotometry for the detection of various metals [48,49,50].

The use of TPP[B_10_H_9_NH=C(CH_3_)C(COOC_2_H_5_)=PPh_3_] allows for the selective identification of TPP^+^ in the presence of Bu_4_N^+^ with a selectivity coefficient (pK^pot^ _TPP/Cation_) of 2.3. This is achieved thanks to TPP[B_10_H_9_NH=C(CH_3_)C(COOC_2_H_5_)=PPh_3_], which acts as an ion pair. Its lipophilic anionic component is water-insoluble and remains stable over time, maintaining a stable equilibrium between TPP^+^ ions in the membrane phase and the solution.

The low detection limit is attributed to the low content of this membrane component, just 1%, which reduces ion diffusion from the membrane into the solution when there is zero current.

The proposed TPP-selective membrane sensor offers valuable performance features, making it effective for the quick detection of tetraphenylphosphonium chloride, whether in its pure form or in complex salt solutions.

The sensing mechanism can be described within the classical PVC-membrane ISE concept. The membrane incorporates a lipophilic anionic species (the functionalized *closo*-decaborate), which provides ion-exchange/ion-association sites for bulky organic cations. Upon contact with the sample, TPP^+^ is preferentially extracted into the membrane phase and forms an ion pair with the lipophilic anion, giving rise to a phase-boundary potential. The near-Nernstian slope observed for TPP^+^ is consistent with the response expected for a monovalent cation. The selectivity pattern (including discrimination against Bu_4_N^+^) is attributed to differences in the partitioning/ion-pairing equilibria of competing cations in the plasticized PVC membrane.

Some undoubtedly interesting questions remain open. One such question is the mechanism of ion exchange of the large TPP cation between the membrane and solution phases. It is known that the boron cluster carries a permanent negative charge and participates in electrostatic interactions. Additionally, the specific distribution of the functionalized boron cluster within the polymer matrix—whether it is homogeneously dispersed or forms aggregates—has not yet been characterized by surface analysis methods (e.g., SEM/EDX). The influence of such microstructural factors on the sensitivity and selectivity needs further exploration. We hope that these questions will attract the interest of researchers, and that answers to them will facilitate the development of functional materials with specific electroanalytical properties.

## 3. Materials and Methods

### 3.1. NMR Spectroscopy

^1^H, ^31^P{^1^H}, ^13^C{^1^H}, ^11^B{^1^H} NMR spectra of solutions in CD_2_Cl_2_ or (CD_3_)_2_CO were recorded on a q-ONE AS400 Quantum I Plus spectrometer (Q.One Instruments Ltd., Wuhan, China) at frequencies of 399.879; 161.874; 100.549; 128.297 MHz, respectively, with internal deuterium stabilization. TMS, 85% phosphoric acid and BF_3_·OEt_2_ were used as external standards, respectively.

### 3.2. High-Resolution Mass Spectroscopy

High-resolution mass spectra were recorded on a LCMS-9030 device (Shimadzu, Kyoto, Japan) by electrospray ionization mass spectrometry (ESI-MS). Measurements were carried out in negative and positive ion mode; samples were dissolved in acetonitrile and injected into the mass-spectrometer chamber from an HPLC system LC-40 Nexera (Shimadzu, Kyoto, Japan). The following parameters were used: capillary voltage −3.0 kV (4 kV for positive ions); mass scanning range: *m*/*z* 50–1000; external calibration with a NaI solution in MeOH/H_2_O; drying and heating gases (nitrogen) (each 10 L/min); nebulizing gas (nitrogen) (3 L/min); interface temperature: 250 °C; and flow rate 100% acetonitrile or (50:50 (*v*/*v*) acetonitrile/water 0.4 mL/min. Molecular ions in the spectra were analyzed and matched with the appropriately calculated *m*/*z* and isotopic profiles in the LabSolutions v.5.114 program.

### 3.3. X-Ray Diffraction Experiments

Diffraction data were collected at 20 °C on a Bruker KAPPA APEX II automatic four-circle diffractometer equipped with a CCD area detector (Bruker AXS Inc.: Madison, WI, USA) (MoKα radiation, λ = 0.71073 Å) [51]. The experiment was performed at the Center for the collective use of physical methods of research of the Frumkin Institute of Physical Chemistry and Electrochemistry of the Russian Academy of Sciences (IPCE RAS). The unit cell parameters were refined using the entire data set [52]. The structure was solved by the direct method [53] and refined by full-matrix least-squares on F^2^ in the anisotropic approximation for all non-hydrogen atoms [54]. The hydrogen atoms of the boron cluster were located from difference Fourier maps and refined isotropically, with *U_iso_*(H) = 1.2 *U*_eq_(B). The positions of the hydrogen atoms in the organic part of the molecule were calculated geometrically, and their isotropic thermal parameters were set to *U_iso_*(H) = 1.2 *U*_eq_(C) for CH_2_ groups and 1.5 *U*_eq_(C) for methyl groups. The absolute structure was not determined. Crystal structure images were prepared using the OLEX2 software package (version 1.5) [55].

Single crystals suitable for analysis were grown by isothermal crystallization from an acetonitrile/1-hexanol solvent mixture. The atomic coordinates have been deposited with the Cambridge Crystallographic Data Centre (CCDC 2497196 and 2497197). Key crystallographic data are presented in Table S1.

### 3.4. Potentiometric Characteristic Measurements

Working standard solutions of tetraphenylphosphonium chloride (TPPCl) were prepared in a concentration range of 10^−1^–10^−8^ mol/L by serial dilution. Constant pH and ionic strength were maintained during the measurements.

The electrode membranes were fabricated using a standard potentiometric technique [56]. To prepare a PVC-matrix membrane, a weighted sample of the *closo*-decaborate salt (7.5–30.0 mg) was dissolved in 1.0 mL of bis(1-butylpentyl) adipate (BBPA) plasticizer. This solution was then mixed with a solution of 435 mg of PVC in 10 mL of tetrahydrofuran (THF). The resulting membrane cocktail was degassed by sonication for 30 min and cast into a glass ring (28 mm i.d.) fixed on a Petri dish. The solvent was allowed to evaporate at room temperature for approximately 48 h to form a polymer film with a thickness of about 300 µm. To ensure the reproducibility of the potentiometric characteristics, the homogeneity of the mixture and the evaporation rate of THF were carefully controlled.

Disks with a diameter of 6 mm were punched from the master film and mounted in a standard Philips IS 561 electrode body. The body was filled with an inner solution (10 mM KCl, 1.0 mM TPPCl). Before use, the assembled sensor was conditioned in a 1.0 mM TPPCl solution for 24 h and subsequently rinsed with distilled water.

**Figure 5:**
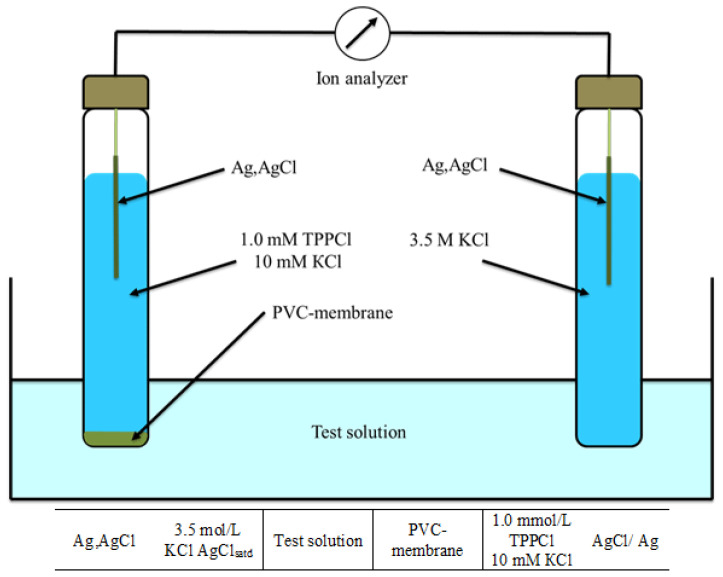
The scheme of the electroanalytical cell.

Potential measurements were carried out at 21 ± 1 °C in stirred solutions using a Radelkis OP-300 pH/ion analyzer (Budapest, Hungary). The electromotive force (EMF) was measured under zero-current conditions for the electrochemical cell shown in Figure 5. A Radelkis OP 0820 silver/silver chloride electrode (Radelkis Kft., Budapest, Hungary) served as the external reference electrode. The potential was recorded after stabilizing to within ±0.2 mV. Calibration plots were constructed by plotting the potential versus −log[TPPCl]. All electroanalytical parameters were determined in accordance with IUPAC recommendations [47].

### 3.5. Materials

Ethyl bromoacetate (Aldrich, St. Louis, MO, USA, 98%), chloroacetonitrile (Aldrich, 99%), and triphenylphosphine (Aldrich, 99%) were used without additional purification. All solvents were purified according to the literature method [57]. Phosphorus ylides were prepared according to literature procedures [58] (for Ph_3_P=CH-COOEt (**2a**)) and [59] (for Ph_3_P=CH-CN (**2b**). Nitrilium derivatives of *closo*-decaborate anions were synthesized by known procedures [30,60].

### 3.6. General Synthetic Procedure for Compounds (Bu_4_N)[2-B_10_H_9_NH=C(R^1^)C(PPh_3_)R^2^] (R^2^ = COOEt, R^1^ = Me *(**3a**)*, Et *(**3b**)*, ^n^Pr *(**3c**)*, Ph *(**3e**)*; R^2^ = CN, R^1^ = Me *(**4a**)*, Et *(**4b**)*, ^n^Pr *(**4c**)*, Ph *(**4e**)*), (Ph_4_P)[2-B_10_H_9_NH=C(Me)C(PPh_3_)COOEt] (Ph_4_P)3(a)

A solution of the respective nitrilium derivative (0.65 mmol) and the phosphorus ylide (0.65 mmol) in dichloromethane (5 mL) was stirred for 30 min under a dry argon atmosphere at room temperature. The solution was then concentrated under reduced pressure, and the residue was purified by flash chromatography on silica gel, eluting with a mixture of DCM/acetonitrile (5:1). The collected target fraction was concentrated, and the product was recrystallized from an ethanol/isopropanol mixture and dried over P_2_O_5_.

### 3.7. Synthetic Procedure for Compounds (Bu_4_N)[2-B_10_H_9_NH=C(^i^Pr)C(PPh_3_)R^2^] (R^2^ = COOEt *(**3d**)*, CN *(**4d**)*)

A solution of the nitrilium derivative **2d** (0.5 mmol) and the phosphorus ylide (2.5 mmol) in MeCN (5 mL) was stirred for 5 h under a dry argon atmosphere at 70 °C. The solution was concentrated under reduced pressure, and the residue was purified by flash chromatography on silica gel, eluting with a mixture of DCM/acetonitrile (5:1). The collected target fraction was concentrated, and the product was recrystallized from an ethanol/isopropanol mixture and dried over P_2_O_5_.


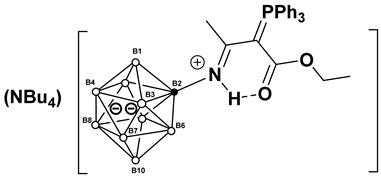


(Bu_4_N)[2-B_10_H_9_NHC(Ph_3_PCCOOEt)CH_3_)] (**3a**). Yield 340 mg (91%).

^11^B [^1^H]-NMR (CD_2_Cl_2_) δ (ppm): −0.6 (s, 1B, B(10)), −3.4 (s, 1B, B(1)), −14.1 (s, 1B, B(2)), −25.3 (s, 4B, B(3,5,6,9)), −28.3 (s, 3B, B(4,7,8)). ^1^H-NMR (CD_2_Cl_2_) δ (ppm): 10.23 (s, 1H, NH), 7.74, 7.65, 7.56 (m, 15H, (C_6_H_5_)_3_P), 3.55 (q, 2H, OCH_2_CH_3_, *J* = 7.2 Hz), 3.18 (8H, NBu_4_), 1.63, 1.62 (m, 11H, NHCCH_3_ + NBu_4_), 1.46 (8H, NBu_4_), 1.00 (12H, NBu_4_), 0.48 (t, 3H, OCH_2_CH_3_, *J* = 7.2 Hz). ^13^C NMR (CD_2_Cl_2_) δ (ppm): 172.9 (d, C=O, *J^C^*^-*P*^ = 16.7 Hz), 168.8 (d, NH=C, *J^C^*^-*P*^ = 10.7 Hz), 133.9 (d, (C_6_H_5_)_3_P *J^C^*^-*P*^ = 9.9 Hz), 133.5 (d, (C_6_H_5_)_3_P, *J^C^*^-*P*^ = 3.2 Hz), 129.8 (d, (C_6_H_5_)_3_P, *J^C^*^-*P*^ = 12.3 Hz), 125.9 (d, (C_6_H_5_)_3_P, *J^C^*^-*P*^ = 92.2 Hz), 63.0 (d, C-C=O, *J^C-P^* = 124.8 Hz), 59.6 (OCH_2_CH_3_), 59.4 (NBu_4_), 24.6 (NBu_4_), 24.1 (d, NHCCH_3_, *J^C^*^-*P*^ = 3.2 Hz), 20.3 (NBu_4_), 14.0 (NBu_4_), 13.8 (OCH_2_CH_3_). ^31^P NMR (CD_2_Cl_2_) δ (ppm): 18.6. HRMS (ESI^−^) (*m*/*z*): 506.3260 (A refers to the molecular weight of [(B_10_H_9_NHCCH_3_(C_6_H_5_)_3_PCCOOC_2_H_5_)], calculated for [A]^−^ 506.3252), 1035.6416 (calculated for {2[A]+Na]^−^} 1035.6402).


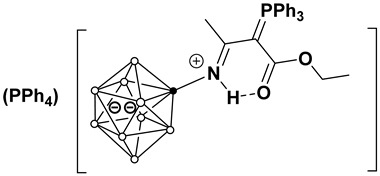


(Ph_4_P)[2-B_10_H_9_NHC(Ph_3_PCCOOEt)CH_3_)] **Ph_4_P(3a)**. Yield 380 mg (95%).

^11^B[^1^H]-NMR ((CD_3_)_2_CO) δ (ppm): 0.1 (s, 1B, B(10)), −3.6 (s, 1B, B(1)), −13.9 (s, 1B, B(2)), −24.9 (s, 4B, B(3,5,6,9)), −28.2 (s, 3B, B(4,7,8)). ^1^H-NMR ((CD_3_)_2_CO) δ (ppm): 10.41 (s, 1H, NH), 8.01, 7.88, 7.85, 7.74, 7.69 (m, 35H, (C_6_H_5_)_3_P + Ph_4_P), 3.46 (q, 2H, OCH_2_CH_3_, *J* = 7.2 Hz), 1.66 (s, 3H, NHCCH_3_), 0.43 (t, 3H, OCH_2_CH_3_, *J* = 7.2 Hz).


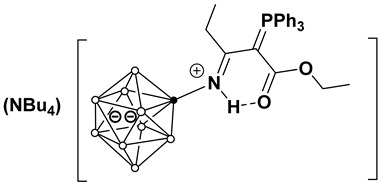


(Bu_4_N)[2-B_10_H_9_NHC(Ph_3_PCCOOEt)C_2_H_5_)] (**3b**). Yield 325 mg (85%).

^11^B[^1^H]-NMR (CD_2_Cl_2_) δ (ppm): −0.6 (s, 1B, B(10)), −2.9 (s, 1B, B(1)), −14.2 (s, 1B, B(2)), −25.1 (s, 4B, B(3,5,6,9)), −27.9 (s, 3B, B(4,7,8)). ^1^H-NMR (CD_2_Cl_2_) δ (ppm): 10.24 (s, 1H, NH), 7.72, 7.65, 7.55 (m, 15H, (C_6_H_5_)_3_P), 3.52 (q, 2H, OCH_2_CH_3_, *J* = 7.2 Hz), 3.17 (8H, NBu_4_), 2.19 (q, 2H, NH=CCH_2_CH_3_, *J* = 7.4 Hz), 1.61 (8H, NBu_4_), 1.45 (8H, NBu_4_), 1.00 (12H, NBu_4_), 0.68 (t, 3H, NH=CCH_2_CH_3_, *J* = 7.4 Hz), 0.48 (t, 3H, OCH_2_CH_3_, *J* = 7.2 Hz). ^13^C NMR (CD_2_Cl_2_) δ (ppm): 178.5 (d, C=O, *J^C^*^-*P*^ = 16.7 Hz), 169.0 (d, NH=C, *J^C^*^-*P*^ = 10.3 Hz), 133.8 (d, (C_6_H_5_)_3_P *J^C^*^-*P*^ = 9.5 Hz), 133.4 (d, (C_6_H_5_)_3_P, *J^C^*^-*P*^ = 3.2 Hz), 129.8 (d, (C_6_H_5_)_3_P, *J^C^*^-*P*^ = 12.3 Hz), 126.4 (d, (C_6_H_5_)_3_P, *J^C^*^-*P*^ = 92.2 Hz), 61.2 (d, C-C=O, *J^C^*^-*P*^ = 124.0 Hz), 59.7 (OCH_2_CH_3_), 59.3 (NBu_4_), 26.7 (d, NHCCH_2_CH_3_, *J^C^*^-*P*^ = 3.2 Hz), 24.6 (NBu_4_), 20.3 (NBu_4_), 14.0 (NBu_4_), 13.6 (NHCCH_2_CH_3_ + OCH_2_CH_3_). ^31^P NMR (CD_2_Cl_2_) δ (ppm): 17.9. HRMS (ESI^−^) (*m*/*z*): 520.3413 (A refers to the molecular weight of [(B_10_H_9_NHCC_2_H_5_(C_6_H_5_)_3_PCCOOC_2_H_5_)], calculated for [A]^−^ 520.3409), 632.3180 (calculated for {[A] + Na + 2HCOO-H]^−^} 632.3181), 1063.6724 (calculated for {2[A] + Na]^−^} 1063.6712).


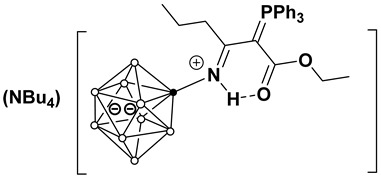


(Bu_4_N)[2-B_10_H_9_NHC(Ph_3_PCCOOEt)^n^C_3_H_7_)] (**3c**). Yield 352 mg (90%).

^11^B[^1^H]-NMR (CD_2_Cl_2_) δ (ppm): −0.6 (s, 1B, B(10)), −3.0 (s, 1B, B(1)), −14.2 (s, 1B, B(2)), −25.3 (s, 4B, B(3,5,6,9)), −27.9 (s, 3B, B(4,7,8)). ^1^H-NMR (CD_2_Cl_2_) δ (ppm): 10.29 (s, 1H, NH), 7.70, 7.65, 7.55 (m, 15H, (C_6_H_5_)_3_P), 3.52 (q, 2H, OCH_2_CH_3_, *J* = 7.1 Hz), 3.17 (8H, NBu_4_), 2.09 (m, 2H, NH=CCH_2_CH_2_CH_3_), 1.61 (8H, NBu_4_), 1.45 (8H, NBu_4_), 1.21 (m, 2H, NH=CCH_2_CH_2_CH_3_), 1.00 (12H, NBu_4_), 0.41 (t, 6H, OCH_2_CH_3_ + NHCCH_2_CH_2_CH_3_, *J* = 7.2 Hz). ^13^C NMR (CD_2_Cl_2_) δ (ppm): 177.2 (d, C=O, *J^C^*^-*P*^ = 16.7 Hz), 169.0 (d, NH=C, *J^C^*^-*P*^ = 10.3 Hz), 133.8 (d, (C_6_H_5_)_3_P *J^C^*^-*P*^ = 9.9 Hz), 133.3 (d, (C_6_H_5_)_3_P, *J^C^*^-*P*^ = 3.2 Hz), 129.8 (d, (C_6_H_5_)_3_P, *J^C^*^-*P*^ = 12.7 Hz), 126.6 (d, (C_6_H_5_)_3_P, *J^C^*^-*P*^ = 92.2 Hz), 61.4 (d, C-C=O, *J^C^*^-*P*^ = 123.6 Hz), 59.7 (OCH_2_CH_3_), 59.3 (NBu_4_), 35.1 (NHCCH_2_CH_2_CH_3_), 24.6 (NBu_4_), 22.4 (NHCCH_2_CH_2_CH_3_), 20.3 (NBu_4_), 14.0 (NBu_4_), 13.8 (OCH_2_CH_3_), 13.6 (NHCCH_2_CH_2_CH_3_). ^31^P NMR (CD_2_Cl_2_) δ (ppm): 17.9. HRMS (ESI^−^) (*m*/*z*): 535.3542 (A refers to the molecular weight of [(B_10_H_9_NHCC_3_H_7_(C_6_H_5_)_3_PCCOOC_2_H_5_)], calculated for [A]^−^ 535.3565), 647.3308 (calculated for {[A] + Na + 2HCOO-H]^−^} 647.3301), 1092.6996 (calculated for {2[A] + Na]^−^} 1092.6992).


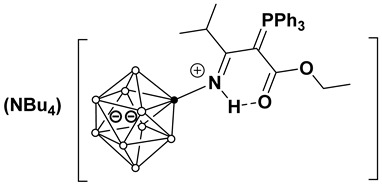


(Bu_4_N)[2-B_10_H_9_NHC(Ph_3_PCCOOEt)^i^C_3_H_7_)] (**3d**). Yield 311 mg (80%).

^11^B[^1^H]-NMR (CD_2_Cl_2_) δ (ppm): −0.6 (s, 1B, B(10)), −2.3 (s, 1B, B(1)), −13.9 (s, 1B, B(2)), −25.1 (s, 4B, B(3,5,6,9)), −27.7 (s, 3B, B(4,7,8)). ^1^H-NMR (CD_2_Cl_2_) δ (ppm): 8.81 (s, 1H, NH), 7.71, 7.66, 7.55 (m, 15H, (C_6_H_5_)_3_P), 3.59 (q, 2H, OCH_2_CH_3_, *J* = 7.1 Hz), 3.15 (8H, NBu_4_), 3.02 (m, 1H, NH=CCH(CH_3_)_2_), 1.60 (8H, NBu_4_), 1.44 (8H, NBu_4_), 1.13 (d, 6H, NH=CCH(CH_3_)_2_, *J* = 7.1 Hz), 1.00 (12H, NBu_4_), 0.61 (t, 3H, OCH_2_CH_3_, *J* = 7.1 Hz). ^13^C NMR (CD_2_Cl_2_) δ (ppm): 183.3 (d, C=O, *J^C-P^* =12.3 Hz), 168.4 (d, NH=C, *J^C-P^* = 11.5 Hz), 133.7 (d, (C_6_H_5_)_3_P, *J^C-P^* =9.9 Hz), 133.2 (d, (C_6_H_5_)_3_P, *J^C-P^* = 3.2 Hz), 129.9 (d, (C_6_H_5_)_3_P, *J^C-P^* = 12.7 Hz), 125.9 (d, (C_6_H_5_)_3_P, *J^C-P^* = 91.8 Hz), 60.6 (d, C-C=O, *J^C-P^* = 122.4 Hz), 59.5 (OCH_2_CH_3_), 59.3 (NBu_4_), 37.4 (d, NH=CH(CH_3_)_2_, *J^C-P^* = 4.7 Hz), 24.6 (NBu_4_), 20.2 (NBu_4_), 19.2 (NH=CH(CH_3_)_2_), 14.0 (NBu_4_ + OCH_2_CH_3_). ^31^P NMR (CD_2_Cl_2_) δ (ppm): 18.4. HRMS (ESI^−^) (*m*/*z*): 535.3542 (A refers to the molecular weight of [(B_10_H_9_NHCC_3_H_7_(C_6_H_5_)_3_PCCOOC_2_H_5_)], calculated for [A]^−^ 535.3565), 647.3308 (calculated for {[A] + Na + 2HCOO-H]^−^} 647.3301), 1092.7005 (calculated for {2[A] + Na]^−^} 1092.6992).


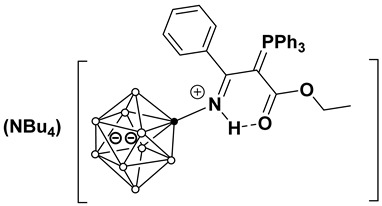


(Bu_4_N)[2-B_10_H_9_NHC(Ph_3_PCCOOEt)C_6_H_5_)] (**3e**). Yield 353 mg (87%).

^11^B[^1^H]-NMR (CD_2_Cl_2_) δ (ppm): −1.0 (s, 1B, B(10)), −2.5 (s, 1B, B(1)), −13.9 (s, 1B, B(2)), −25.3 (s, 4B, B(3,5,6,9)), −28.0 (s, 2B, B(7,8)), −30.1 (s, 1B, B(4)). ^1^H-NMR (CD_2_Cl_2_) δ (ppm): 10.43 (s, 1H, NH), 7.49, 7.46, 7.38 (m, 15H, (C_6_H_5_)_3_P), 6.99, 6.83, 6.73 (m, 5H, NH=C-C_6_H_5_), 3.57 (q, 2H, OCH_2_CH_3_, *J* = 7.1 Hz), 3.10 (8H, NBu_4_), 1.57 (8H, NBu_4_), 1.41 (8H, NBu_4_), 0.99 (12H, NBu_4_), 0.44 (t, 3H, OCH_2_CH_3_, *J* = 7.2 Hz). ^13^C NMR (CD_2_Cl_2_) δ (ppm): 174.6 (d, C=O, *J^C-P^* = 16.7 Hz), 169.2 (d, NH=C, *J^C-P^* = 10.9 Hz), 135.9 (NH=C-C_6_H_5_), 133.6 (d, (C_6_H_5_)_3_P *J^C-P^*=9.5 Hz), 132.7 (d, (C_6_H_5_)_3_P, *J^C-P^* = 3.2 Hz), 130.6 (NH=C-C_6_H_5_), 129.4 (d, (C_6_H_5_)_3_P, *J^C-P^* = 12.3 Hz), 129.3 (NH=C-C_6_H_5_), 127.8 (NH=C-C_6_H_5_), 125.5 (d, (C_6_H_5_)_3_P, *J^C-P^* = 93.0 Hz), 63.7 (d, C-C=O, *J^C-P^* = 111.7 Hz), 60.0 (OCH_2_CH_3_), 59.3 (NBu_4_), 24.6 (NBu_4_), 20.3 (NBu_4_), 14.0 (NBu_4_), 13.6 (OCH_2_CH_3_). ^31^P NMR (CD_2_Cl_2_) δ (ppm): 19.4. HRMS (ESI^−^) (*m*/*z*): 535.3542 (A refers to the molecular weight of [(B_10_H_9_NHCC_3_H_7_(C_6_H_5_)_3_PCCOOC_2_H_5_)], calculated for [A]^−^ 535.3565), 647.3308 (calculated for {[A] + Na + 2HCOO-H]^−^} 647.3301), 1092.6996 (calculated for {2[A] + Na]^−^} 1092.6992).


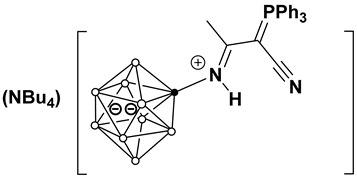


(Bu_4_N)[2-B_10_H_9_NHC(Ph_3_PCCN)CH_3_)] (**4a**). Yield 327 mg (93%).

^11^B[^1^H]-NMR (CD_2_Cl_2_) δ (ppm): −0.6 (s, 1B, B(10)), −3.7 (s, 1B, B(1)), −13.8 (s, 1B, B(2)), −25.4 (s, 4B, B(3,5,6,9)), −28.8 (s, 3B, B(4,7,8)). ^1^H-NMR (CD_2_Cl_2_) δ (ppm): 7.78, 7.62, 7.59 (m, 15H, (C_6_H_5_)_3_P), 6.91 (s, 1H, NH), 3.14 (8H, NBu_4_), 2.15 (br.s, 3H, NH=CCH_3_), 1.60 (8H, NBu_4_), 1.43 (8H, NBu_4_), 0.99 (12H, NBu_4_). ^13^C NMR (CD_2_Cl_2_) δ (ppm): 174.3 (NH=C), 135.0 (C_6_H_5_)_3_P), 134.2 (d, (C_6_H_5_)_3_P, *J^C-P^* = 10.3 Hz), 130.5 (d, (C_6_H_5_)_3_P, *J^C-P^* = 12.7 Hz), 121.3 (C-CN), 119.9 ((C_6_H_5_)_3_P), 59.3 (NBu_4_), 44.0 (d, C-CN, *J^C-P^* = 120.7 Hz), 24.5 (NBu_4_), 22.4 (d, NHCCH_3_, *J^C-P^* = 4.4 Hz), 20.2 (NBu_4_), 14.0 (NBu_4_). ^31^P NMR (CD_2_Cl_2_) δ (ppm): 17.0. HRMS (ESI^−^) (*m*/*z*): 459.3002 (A refers to the molecular weight of [(B_10_H_9_NHCCH_3_(C_6_H_5_)_3_PCCN)], calculated for [A]^−^ 459.2993).


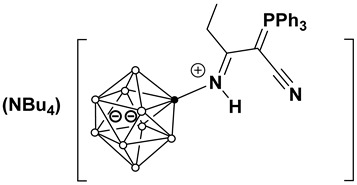


(Bu_4_N)[2-B_10_H_9_NHC(Ph_3_PCCN)C_2_H_5_)] (**4b**). Yield 320 mg (89%).

^11^B[^1^H]-NMR (CD_2_Cl_2_) δ (ppm): −0.7 (s, 1B, B(10)), −3.6 (s, 1B, B(1)), −14.3 (s, 1B, B(2)), −25.4 (s, 4B, B(3,5,6,9)), −28.7 (s, 2B, B(7,8)), −30.0 (s, 1B, B(4)). ^1^H-NMR (CD_2_Cl_2_) δ (ppm): 7.79, 7.65, 7.54 (m, 15H, (C_6_H_5_)_3_P), 5.79 (s, 1H, NH), 3.13 (10H, NH=CCH_2_CH_3_ + NBu_4_), 1.58 (8H, NBu_4_), 1.44 (8H, NBu_4_), 1.30 (br.m, 3H, NH=CCH_2_CH_3_), 0.98 (12H, NBu_4_). ^13^C NMR (CD_2_Cl_2_) δ (ppm): 180.7 (NH=C), 135.2 (C_6_H_5_)_3_P), 134.3 (d, (C_6_H_5_)_3_P, *J^C-P^* = 10.7 Hz), 130.7 (d, (C_6_H_5_)_3_P, *J^C-P^* = 12.7 Hz), 120.4 (d, (C_6_H_5_)_3_P, *J^C-P^* = 89.0 Hz), 120.5 (d, CN, *J^C-P^* = 13.9 Hz), 59.3 (NBu_4_), 43.1 (d, C-CN, *J^C-P^* = 120.4 Hz), 28.1 (NHCCH_2_CH_3_), 24.5 (NBu_4_), 20.2 (NBu_4_), 14.0 (NBu_4_), 12.8 (NHCCH_2_CH_3_). ^31^P NMR (CD_2_Cl_2_) δ (ppm): 16.9. HRMS (ESI^−^) (*m*/*z*): 473.3157 (A refers to the molecular weight of [(B_10_H_9_NHCC_2_H_5_(C_6_H_5_)_3_PCCN)], calculated for [A]^−^ 473.3150).


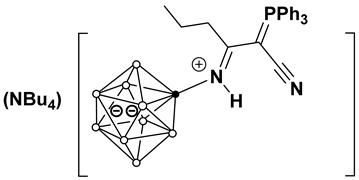


(Bu_4_N)[2-B_10_H_9_NHC(Ph_3_PCCN)^n^C_3_H_7_)] (**4c**). Yield 318 mg (87%).

^11^B[^1^H]-NMR (CD_2_Cl_2_) δ (ppm): −0.7 (s, 1B, B(10)), −3.6 (s, 1B, B(1)), −14.2 (s, 1B, B(2)), −25.3 (s, 4B, B(3,5,6,9)), −28.6 (s, 2B, B(7,8)), −29.7 (s, 1B, B(4)). ^1^H-NMR (CD_2_Cl_2_) δ (ppm): 7.78, 7.65, 7.56 (m, 15H, (C_6_H_5_)_3_P), 5.78 (s, 1H, NH), 3.11 (8H, NBu_4_), 3.05 (br.m, 2H, NH=CCH_2_CH_2_CH_3_), 1.78 (br.m, 2H, NH=CCH_2_CH_2_CH_3_), 1.58 (8H, NBu_4_), 1.44 (8H, NBu_4_), 0.99 (m, 15H, NBu_4_ + NH=CCH_2_CH_2_CH_3_). ^13^C NMR (CD_2_Cl_2_) δ (ppm): 179.3 (NH=C), 135.0 ((C_6_H_5_)_3_P,), 134.3 (d, (C_6_H_5_)_3_P, *J^C-P^* = 10.3 Hz), 130.5 (d, (C_6_H_5_)_3_P, *J^C-P^* = 13.1 Hz), 120.5 (d, (C_6_H_5_)_3_P, *J^C^*^-*P*^= 89.2 Hz), 120.3 (d, CN, *J^C-P^* = 12.7 Hz), 59.3 (NBu_4_), 44.1 (d, C-CN, *J^C-P^* = 120.0 Hz), 30.2 (NHCCH_2_CH_2_CH_3_), 24.5 (NBu_4_), 22.4 (NHCCH_2_CH_2_CH_3_).20.2 (NBu_4_), 14.0 (NBu_4_ + NHCCH_2_CH_2_CH_3_). ^31^P NMR (CD_2_Cl_2_) δ (ppm): 16.9. HRMS (ESI^−^) (*m*/*z*): 487.3315 (A refers to the molecular weight of [(B_10_H_9_NHCC_3_H_7_(C_6_H_5_)_3_PCCN)], calculated for [A]^−^ 487.3306).


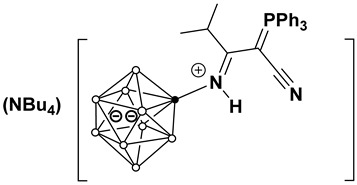


(Bu_4_N)[2-B_10_H_9_NHC(Ph_3_PCCN)^n^C_3_H_7_)] (**4d**). Yield 300 mg (82%).

^11^B[^1^H]-NMR (CD_2_Cl_2_) δ (ppm): −0.9 (s, 1B, B(10)), −3.1 (s, 1B, B(1)), −14.2 (s, 1B, B(2)), −25.2 (s, 4B, B(3,5,6,9)), −28.9 (s, 2B, B(7,8)), −29.8 (s, 1B, B(4)). ^1^H-NMR (CD_2_Cl_2_) δ (ppm): 7.79, 7.65, 7.55 (m, 15H, (C_6_H_5_)_3_P), 5.73 (s, 1H, NH), 4.29 (m, 1H, NH=CCH(CH_3_)_2_), 3.11 (8H, NBu_4_), 1.58 (8H, NBu_4_), 1.42 (14H, NBu_4_ + NH=CCH(CH_3_)_2_), 1.00 (12H, NBu_4_). ^13^C NMR (CD_2_Cl_2_) δ (ppm): 183.6 (NH=C), 135.2 (d, (C_6_H_5_)_3_P, *J^C-P^*=3.2 Hz), 134.4 (d, (C_6_H_5_)_3_P, *J^C-P^*=10.4 Hz), 130.6 (d, (C_6_H_5_)_3_P, *J^C-P^*=13.1 Hz), 120.3 (d, (C_6_H_5_)_3_P, *J^C-P^*=90.2 Hz), 120.6 (CN), 59.3 (NBu_4_), 43.5 (d, C-CN, *J^C-P^*=120.3 Hz), 32.6 (NHCH(CH_3_)_2_), 24.5 (NBu_4_), 20.2 (NBu_4_), 18.8 (NHCH(CH_3_)_2_), 14.0 (NBu_4_). ^31^P NMR (CD_2_Cl_2_) δ (ppm): 19.4. HRMS (ESI^−^) (*m*/*z*): 487.3314 (A refers to the molecular weight of [(B_10_H_9_NHCC_3_H_7_(C_6_H_5_)_3_PCCN)], calculated for [A]^−^ 487.3306).


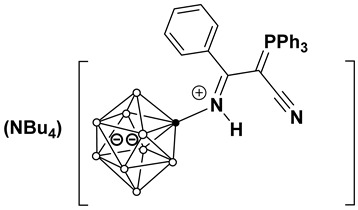


(Bu_4_N)[2-B_10_H_9_NHC(Ph_3_PCCN)C_6_H_5_)] (**4e**). Yield 351 mg (92%).

^11^B[^1^H]-NMR (CD_2_Cl_2_) δ (ppm): −1.6 (s, 1B, B(10)), −3.7 (s, 1B, B(1)), −14.0 (s, 1B, B(2)), −25.8 (s, 4B, B(3,5,6,9)), −29.0 (s, 2B, B(7,8)), −30.6 (s, 1B, B(4)). ^1^H-NMR (CD_2_Cl_2_) δ (ppm): 7.75, 7.63, 7.41 (br.m, 20H, (C_6_H_5_)_3_P+ NH=CC_6_H_5_), 6.09 (s, 1H, NH), 3.06 (8H, NBu_4_), 1.53 (8H, NBu_4_), 1.39 (8H, NBu_4_), 0.98 (12H, NBu_4_). ^13^C NMR (CD_2_Cl_2_) δ (ppm): 176.4 (NH=C), 134.3 (d, (C_6_H_5_)_3_P, *J^C-P^* = 10.3 Hz), 130.5, 127.9 ((C_6_H_5_)_3_P + NH=CC_6_H_5_), 120.4 (CN), 59.2 (NBu_4_), 46.1 (C-CN, *J^C-P^* = 122.8 Hz), 24.5 (NBu_4_), 20.2 (NBu_4_), 14.0 (NBu_4_). ^31^P NMR (CD_2_Cl_2_) δ (ppm): 17.4. HRMS (ESI^−^) (*m*/*z*): 522.3113 (A refers to the molecular weight of [(B_10_H_9_NHCC_6_H_5_(C_6_H_5_)_3_PCCN)], calculated for [A]^−^ 522.3129).

## 4. Conclusions

In summary, we have developed a novel and efficient methodology for the functionalization of the *closo*-decaborate cluster via the nucleophilic addition of stabilized phosphorus ylides to its nitrilium derivatives. This reaction provides a direct and high-yielding route to a new family of boron–hybrid iminoacyl phosphoranes, which were comprehensively characterized. The study revealed a significant steric effect of the substituent R^1^ on the reactivity of the nitrilium precursor.

Furthermore, we demonstrated the practical utility of these novel hybrid materials by implementing one of the synthesized anions as a membrane-active component in ion-selective electrodes. The fabricated tetraphenylphosphonium sensor exhibited outstanding analytical performance, including a low detection limit, high selectivity, and a wide linear range. This work not only expands the chemistry of boron clusters by introducing a new class of functional derivatives but also paves the way for their application in electroanalytical chemistry, particularly in the determination of biologically and industrially relevant organic cations.

## Figures and Tables

**Table 1:** Potentiometric response characteristics of TPP sensor as a function of the membrane composition.

№	Membrane Composition, % wt.			Linear Range, mol/L	Lower Detection Limit, mol/L	Slope, mV/Decade
	Membrane Active Compound	Bis(1-butylpentyl)adipate	PVC			
1	0.5	70.5	29.0	≈10^−7^–10^−2^	≈1 × 10^−8^	≈58 ± 2
2	1.0	70.0	29.0	1 × 10^−7^–10^−2^	3 × 10^−8^	57.2 ± 0.3
3	1.5	69.5	29.0	5 × 10^−7^–10^−2^	8 × 10^−8^	55.8 ± 0.2
4	2.0	69.0	29.0	1 × 10^−6^–10^−2^	3 × 10^−7^	53.2 ± 0.1
5	2.5	68.5	29.0	5 × 10^−6^–10^−2^	7 × 10^−7^	51.2 ± 0.2
6	3.0	68.0	29.0	1 × 10^−6^–10^−2^	9 × 10^−7^	50.1 ± 0.2

**Table 2:** Potentiometric selectivity coefficients obtained for TPP sensor.

Interfering Cation	Li^+^	Na^+^	K^+^	Rb^+^	Cs^+^	Mg^2+^	Ca^2+^	Sr^2+^	Ba^2+^	NH_4_^+^	Pb^2+^	H^+^	Bu_4_N^+^
pK^pot^ _TPP/Cation_	7.0	7.1	5.6	7.0	5.6	7.1	7.3	7.4	7.5	6.8	6.4	4.7	2.3

## Data Availability

Date on the compounds are available from the authors.

## Supplementary Materials

The following supporting information can be downloaded at: https://www.mdpi.com/article/10.3390/molecules31020231/s1, the supplementary information includes NMR spectra, ESI-HRMS spectra, crystal structure data files, and raw potentiometric data.
