# Supplementary material for: Nucleophilic Addition of Stabilized Phosphorus Ylides to Closo-Decaborate Nitrilium Salts: A Synthetic Route to Boron Cluster-Functionalized Iminoacyl Phosphoranes and Their Application in Potentiometric Sensing
Source: Molecules. 2026 Jan 9;31(2):231. doi: 10.3390/molecules31020231 (PMC12843826; doi:10.3390/molecules31020231)

# checkCIF/PLATON report

Structure factors have been supplied for datablock(s) Zh101

THIS REPORT IS FOR GUIDANCE ONLY. IF USED AS PART OF A REVIEW PROCEDURE FOR PUBLICATION, IT SHOULD NOT REPLACE THE EXPERTISE OF AN EXPERIENCED CRYSTALLOGRAPHIC REFEREE.

No syntax errors found. CIF dictionary Interpreting this report

# Datablock: Zh101

---

| | | | |
|---|---|---|---|
| Bond precision: | C-C = 0.0134 A | | Wavelength=0.71073 |
| Cell: | a=8.778(3) | b=17.178(4) | c=28.907(8) |
| | alpha=90 | beta=95.345(7) | gamma=90 |
| Temperature: | 100 K | | |

| | Calculated | Reported |
|---|---|---|
| Volume | 4340(2) | 4340(2) |
| Space group | P 21/n | P 21/n |
| Hall group | -P 2yn | -P 2yn |
| Moiety formula | C23 H30 B10 N2 P, C16 H36 N | ? |
| Sum formula | C39 H66 B10 N3 P | C39 H66 B10 N3 P |
| Mr | 716.02 | 716.01 |
| Dx,g cm-3 | 1.096 | 1.096 |
| Z | 4 | 4 |
| Mu (mm-1) | 0.094 | 0.094 |
| F000 | 1544.0 | 1544.0 |
| F000' | 1544.79 | |
| h,k,lmax | 10,20,34 | 10,20,34 |
| Nref | 7653 | 7648 |
| Tmin,Tmax | 0.980,0.997 | |
| Tmin' | 0.980 | |

Correction method= Not given

Data completeness= 0.999 Theta(max)= 24.999

R(reflections)= 0.1328( 2763) wR2(reflections)= 0.3706( 7648)

S = 1.025 Npar= 479

---

The following ALERTS were generated. Each ALERT has the format
**test-name_ALERT_alert-type_alert-level**.
Click on the hyperlinks for more details of the test.

---

## Alert level B

```
RINTA01_ALERT_3_B  The value of Rint is greater than 0.18
            Rint given   0.247
PLAT020_ALERT_3_B The Value of Rint is Greater Than 0.12 .........      0.247 Report
PLAT026_ALERT_3_B Ratio Observed / Unique Reflections (too) Low ..        36% Check
PLAT084_ALERT_3_B High wR2 Value (i.e. > 0.25) ..................       0.37 Report
PLAT340_ALERT_3_B Low Bond Precision on  C-C Bonds ..............    0.01341 Ang.
PLAT910_ALERT_3_B Missing FCF Reflection(s) Below Theta(Min)[Deg]=       4.14 Note
                1  1  0,   0  2  0,   1  2  0,  -1  0  1,   1  0  1,  -1  1  1,
                0  1  1,   1  1  1,  -1  2  1,   0  2  1,   1  2  1,   0  3  1,
                0  0  2,  -1  1  2,   0  1  2,   1  1  2,  -1  2  2,   0  2  2,
                1  2  2,   0  3  2,  -1  0  3,   1  0  3,  -1  1  3,   0  1  3,
                   (   11 More Missing: see the .ckf listing file)
```

---

## Alert level C

```
PLAT018_ALERT_1_C _diffrn_measured_fraction_theta_max .NE. *_full          ! Check
PLAT029_ALERT_3_C _diffrn_measured_fraction_theta_full value Low .      0.973 Why?
PLAT082_ALERT_2_C High R1 Value ..................................       0.13 Report
PLAT094_ALERT_2_C Ratio of Maximum / Minimum Residual Density ....       2.10 Report
PLAT213_ALERT_2_C Atom N2              has ADP max/min Ratio .....        3.2 prolat
PLAT213_ALERT_2_C Atom C11             has ADP max/min Ratio .....        3.2 prolat
PLAT220_ALERT_2_C NonSolvent   Resd 1  C   Ueq(max)/Ueq(min) Range        4.5 Ratio
PLAT234_ALERT_4_C Large Hirshfeld Difference C53      --C54      .       0.18 Ang.
PLAT411_ALERT_2_C Short Inter H...H Contact  H3       ..H41B     .       2.12 Ang.
                                              -x,1-y,1-z  =     3_566 Check
PLAT411_ALERT_2_C Short Inter H...H Contact  H5       ..H41A     .       2.02 Ang.
                                             1-x,1-y,1-z  =     3_666 Check
PLAT420_ALERT_2_C D-H Bond Without Acceptor  N1       --H1A      .     Please Check
PLAT906_ALERT_3_C Large K Value in the Analysis of Variance ......      6.534 Check
PLAT906_ALERT_3_C Large K Value in the Analysis of Variance ......      2.896 Check
PLAT906_ALERT_3_C Large K Value in the Analysis of Variance ......      2.226 Check
PLAT911_ALERT_3_C Missing FCF Refl Between Thmin & STh/L=    0.595         23 Report
                0 20  0,   0 19  1,   0 20  1,   1 20  1,   1 19  2,   0 20  2,
                1 20  2,   1 19  3,   0 20  3,   1 20  3,   1 19  4,   1 20  4,
                1 20  5, -10  0  6, -10  0  8,   2  5  9, -10  0 10, -10  1 11,
              -10  0 12, -10  1 12,  -9  0 17,  -9  1 18,  -9  1 20,
```

---

## Alert level G

```
PLAT007_ALERT_5_G Number of Unrefined Donor-H Atoms .............          1 Report
              H1A
PLAT083_ALERT_2_G SHELXL Second Parameter in WGHT  Unusually Large       8.24 Why ?
```

```
PLAT870_ALERT_4_G ALERTS Related to Twinning Effects Suppressed ..          ! Info
PLAT883_ALERT_1_G Absent Datum for _atom_sites_solution_primary ..     Please Do !
PLAT930_ALERT_2_G FCF-based   Twin Law ( 0 0 1)         Est.d BASF       0.24 Check
PLAT931_ALERT_5_G CIFcalcFCF  Twin Law ( 0 0 1)         Est.d BASF       0.26 Check
PLAT941_ALERT_3_G Average HKL Measurement Multiplicity ..........         3.3 Low
PLAT965_ALERT_2_G The SHELXL WEIGHT Optimisation has not Converged     Please Check
PLAT967_ALERT_5_G Note: Two-Theta Cutoff Value in Embedded .res ..       50.0 Degree
PLAT969_ALERT_5_G The 'Henn et al.' R-Factor-gap value ..........       2.135 Note
              Predicted wR2: Based on SigI**2 17.36 or SHELX Weight 36.14
```

---

```
  0 ALERT level A = Most likely a serious problem - resolve or explain
  6 ALERT level B = A potentially serious problem, consider carefully
 15 ALERT level C = Check. Ensure it is not caused by an omission or oversight
 10 ALERT level G = General information/check it is not something unexpected

  2 ALERT type 1 CIF construction/syntax error, inconsistent or missing data
 11 ALERT type 2 Indicator that the structure model may be wrong or deficient
 12 ALERT type 3 Indicator that the structure quality may be low
  2 ALERT type 4 Improvement, methodology, query or suggestion
  4 ALERT type 5 Informative message, check
```

---

It is advisable to attempt to resolve as many as possible of the alerts in all categories. Often the minor alerts point to easily fixed oversights, errors and omissions in your CIF or refinement strategy, so attention to these fine details can be worthwhile. It is up to the individual to critically assess their own results and, if necessary, seek expert advice.

---

**PLATON version of 26/09/2025; check.def file version of 20/09/2025**

---

# duplicate check

**No duplication found**

---

**Datablock Zh101** - ellipsoid plot

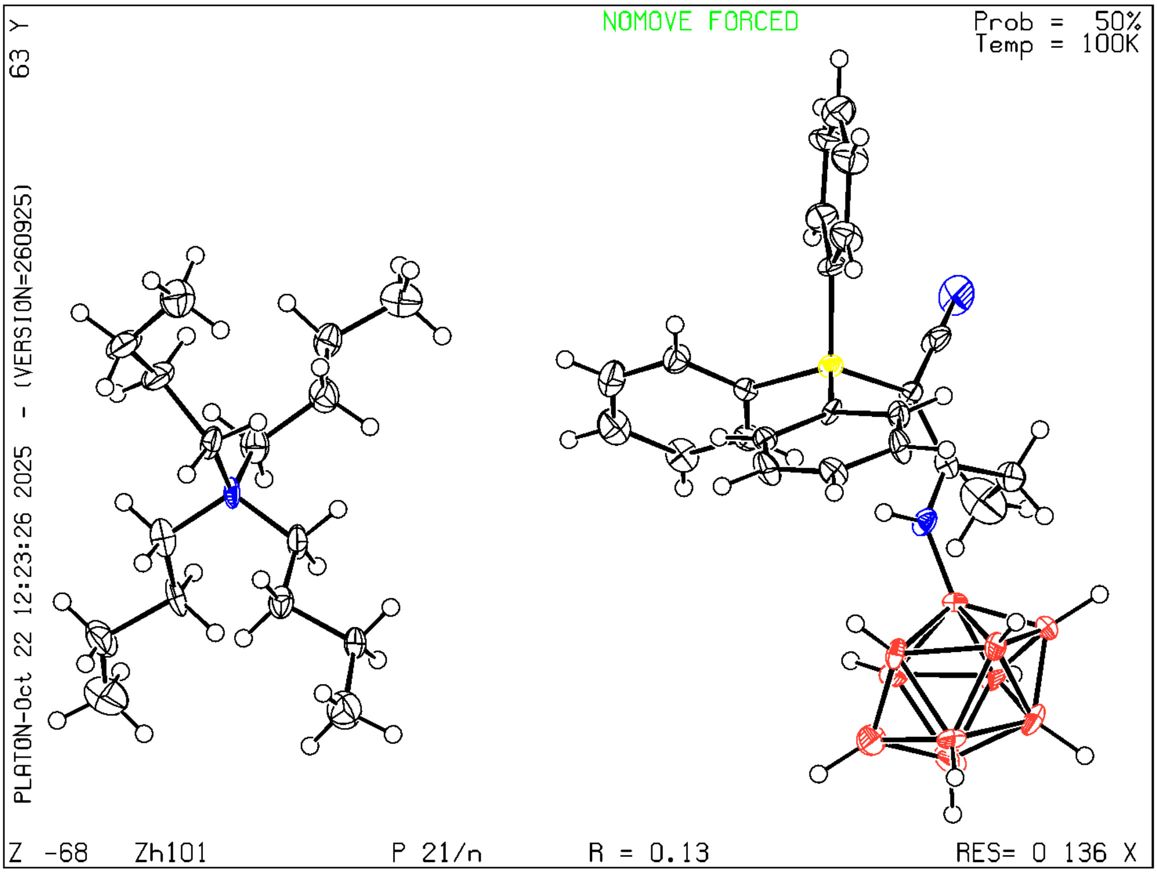

Supplement: Supplementary file 1 [file molecules-31-00231-s001.zip › checkcif_4b.pdf]
