# Supplementary material for: Nucleophilic Addition of Stabilized Phosphorus Ylides to Closo-Decaborate Nitrilium Salts: A Synthetic Route to Boron Cluster-Functionalized Iminoacyl Phosphoranes and Their Application in Potentiometric Sensing
Source: Molecules. 2026 Jan 9;31(2):231. doi: 10.3390/molecules31020231 (PMC12843826; doi:10.3390/molecules31020231)

checkCIF/PLATON report

Structure factors have been supplied for datablock(s) zh67

THIS REPORT IS FOR GUIDANCE ONLY. IF USED AS PART OF A REVIEW PROCEDURE FOR PUBLICATION, IT SHOULD NOT REPLACE THE EXPERTISE OF AN EXPERIENCED CRYSTALLOGRAPHIC REFEREE.

No syntax errors found. CIF dictionary Interpreting this report

Datablock: zh67

Bond precision: C-C = 0.0046 A Wavelength=0.71073

Cell:	a=10.6876(5)	b=13.1011(7)	c=18.1250(9)
	alpha=95.062(2)	beta=101.130(2)	gamma=110.664(2)
Temperature:	100 K		

	Calculated	Reported
Volume	2295.9(2)	2295.9(2)
Space group	P -1	P -1
Hall group	-P 1	-P 1
Moiety formula	C24 H33 B10 N O2 P, C24 H20 P	C24 H33 B10 N O2 P, C24 H20 P
Sum formula	C48 H53 B10 N O2 P2	C48 H53 B10 N O2 P2
Mr	845.95	845.95
Dx,g cm-3	1.224	1.224
Z	2	2
Mu (mm-1)	0.136	0.136
F000	888.0	888.0
F000'	888.68	
h,k,lmax	13,17,23	13,17,23
Nref	10521	10298
Tmin,Tmax	0.971,0.995	
Tmin'	0.950	

Correction method= Not given

Data completeness= 0.979   Theta(max)= 27.498

R(reflections)= 0.0627(5380)   wR2(reflections)= 0.1289(10298)

S = 0.950   Npar= 613

The following ALERTS were generated. Each ALERT has the format
test-name_ALERT_alert-type_alert-level.
Click on the hyperlinks for more details of the test.

Alert level B

```
PLAT910_ALERT_3_B Missing FCF Reflection(s) Below Theta(Min)[Deg]=       4.08 Note
                 1  0  0,  -2  1  0,  -1  1  0,   0  1  0,   1  1  0,  -1  2  0,
                 0  2  0,   0 -2  1,   1 -2  1,  -1 -1  1,   0 -1  1,   1 -1  1,
                -1  0  1,   0  0  1,   1  0  1,  -2  1  1,  -1  1  1,   0  1  1,
                 1  1  1,  -1  2  1,   0  2  1,   0 -2  2,   1 -2  2,  -1 -1  2,
                    (   13 More Missing: see the .ckf listing file)
```

Alert level C

```
PLAT052_ALERT_1_C Info on Absorption Correction Method   Not Given     Please Do !
PLAT241_ALERT_2_C High   'MainMol' Ueq as Compared to Neighbors of       C29 Check
PLAT340_ALERT_3_C Low Bond Precision on  C-C Bonds ..............    0.00456 Ang.
PLAT911_ALERT_3_C Missing FCF Refl Between Thmin & STh/L=    0.600        100 Report
                10  0  0,  11  0  0, -11  1  0, -10  1  0,  10  1  0,  11  1  0,
               -12  2  0, -11  2  0,  10  2  0,  -1  3  0,  10  3  0,  10  4  0,
                 9  5  0,   8  6  0,   8  7  0,   7  8  0, -11 -2  1,  11 -2  1,
                12 -2  1,  10 -1  1,  11 -1  1,   9  0  1,  10  0  1,  11  0  1,
               -12  1  1,   9  1  1,  10  1  1,  11  1  1, -12  2  1,   9  2  1,
                    (   70 More Missing: see the .ckf listing file)
```

Alert level G

```
PLAT002_ALERT_2_G Number of Distance or Angle Restraints on AtSite          3 Note
PLAT007_ALERT_5_G Number of Unrefined Donor-H Atoms .............           1 Report
              H1
PLAT154_ALERT_1_G The s.u.'s on the Cell Angles are Equal ..(Note)      0.002 Degree
PLAT171_ALERT_4_G The CIF-Embedded .res File Contains EADP Records          2 Report
PLAT172_ALERT_4_G The CIF-Embedded .res File Contains DFIX Records          2 Report
PLAT300_ALERT_4_G Atom Site Occupancy of C5A        Constrained at        0.8 Check
PLAT300_ALERT_4_G Atom Site Occupancy of C6A        Constrained at        0.8 Check
PLAT300_ALERT_4_G Atom Site Occupancy of C5B        Constrained at        0.2 Check
PLAT300_ALERT_4_G Atom Site Occupancy of C6B        Constrained at        0.2 Check
PLAT300_ALERT_4_G Atom Site Occupancy of H5AA       Constrained at        0.8 Check
PLAT300_ALERT_4_G Atom Site Occupancy of H5AB       Constrained at        0.8 Check
PLAT300_ALERT_4_G Atom Site Occupancy of H6AA       Constrained at        0.8 Check
PLAT300_ALERT_4_G Atom Site Occupancy of H6AB       Constrained at        0.8 Check
PLAT300_ALERT_4_G Atom Site Occupancy of H6AC       Constrained at        0.8 Check
PLAT300_ALERT_4_G Atom Site Occupancy of H5BA       Constrained at        0.2 Check
PLAT300_ALERT_4_G Atom Site Occupancy of H5BB       Constrained at        0.2 Check
PLAT300_ALERT_4_G Atom Site Occupancy of H6BA       Constrained at        0.2 Check
PLAT300_ALERT_4_G Atom Site Occupancy of H6BB       Constrained at        0.2 Check
PLAT300_ALERT_4_G Atom Site Occupancy of H6BC       Constrained at        0.2 Check
```

```
PLAT301_ALERT_3_G Main Residue  Disorder ..............(Resd    1)          5% Note
PLAT720_ALERT_4_G Number of Unusual/Non-Standard Labels ..........         10 Note
              H5AA    H5AB    H5BA    H5BB    H6AA    H6AB    H6AC    H6BA
              H6BB    H6BC
PLAT860_ALERT_3_G Number of Least-Squares Restraints .............          2 Note
PLAT883_ALERT_1_G Absent Datum for _atom_sites_solution_primary ..     Please Do !
PLAT912_ALERT_4_G Missing # of FCF Reflections Above STh/L=  0.600         86 Note
PLAT933_ALERT_2_G Number of HKL-OMIT Records in Embedded .res File          4 Note
               -3  2  2,  -2 -1  2,  -1  3  0,   1  0  3,
PLAT941_ALERT_3_G Average HKL Measurement Multiplicity ...........        2.0 Low
PLAT967_ALERT_5_G Note: Two-Theta Cutoff Value in Embedded .res ..       55.0 Degree
PLAT969_ALERT_5_G The 'Henn et al.' R-Factor-gap value ...........      1.713 Note
              Predicted wR2: Based on SigI**2  7.53 or SHELX Weight 13.58
PLAT978_ALERT_2_G Number C-C Bonds with Positive Residual Density.          0 Info
```

```
   0 ALERT level A = Most likely a serious problem - resolve or explain
   1 ALERT level B = A potentially serious problem, consider carefully
   4 ALERT level C = Check. Ensure it is not caused by an omission or oversight
  29 ALERT level G = General information/check it is not something unexpected

   3 ALERT type 1 CIF construction/syntax error, inconsistent or missing data
   4 ALERT type 2 Indicator that the structure model may be wrong or deficient
   6 ALERT type 3 Indicator that the structure quality may be low
  18 ALERT type 4 Improvement, methodology, query or suggestion
   3 ALERT type 5 Informative message, check
```

It is advisable to attempt to resolve as many as possible of the alerts in all categories. Often the minor alerts point to easily fixed oversights, errors and omissions in your CIF or refinement strategy, so attention to these fine details can be worthwhile. It is up to the individual to critically assess their own results and, if necessary, seek expert advice.

PLATON version of 26/09/2025; check.def file version of 20/09/2025

Datablock zh67 - ellipsoid plot

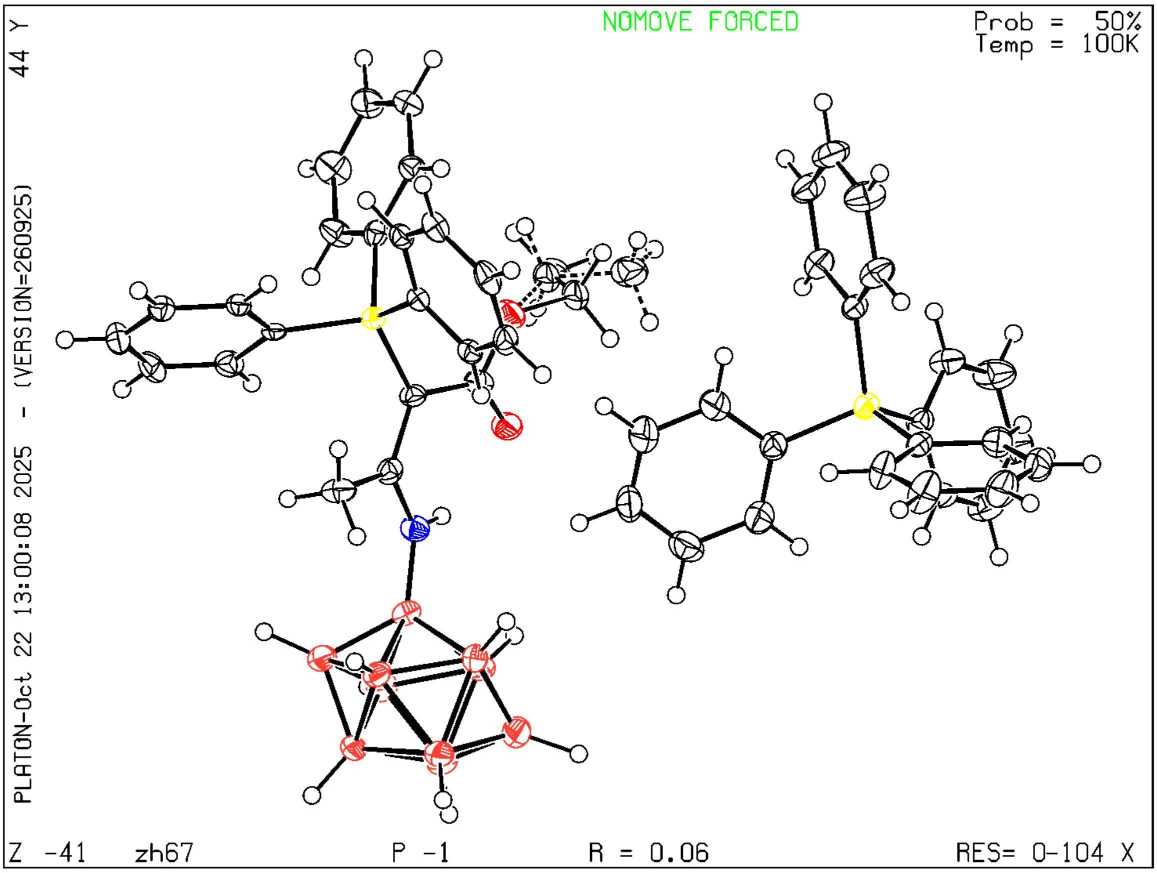

Supplement: Supplementary file 1 [file molecules-31-00231-s001.zip › checkcif_Ph4P_3a.pdf]
