# Supplementary material for: Nucleophilic Addition of Stabilized Phosphorus Ylides to Closo-Decaborate Nitrilium Salts: A Synthetic Route to Boron Cluster-Functionalized Iminoacyl Phosphoranes and Their Application in Potentiometric Sensing
Source: Molecules. 2026 Jan 9;31(2):231. doi: 10.3390/molecules31020231 (PMC12843826; doi:10.3390/molecules31020231)
Supplement: Supplementary file 1 [file molecules-31-00231-s001.zip › Supportihg Information molecules-4038715.pdf]

Supporting Information for

Nucleophilic Addition of Stabilized Phosphorus Ylides to *closo*-Decaborate Nitrilium Salts: A Synthetic Route to Boron Cluster Functionalized Iminoacyl Phosphoranes and Their Application in Potentiometric Sensing

Vera V. Voinova [1], Eugeniy S. Turyshev [2], Sergey S. Novikov [1,3], Nikita A. Selivanov [1], Alexander Yu. Bykov[1], Ilya N. Klyukin [1], Andrey P. Zhdanov [1,*], Mikhail .S. Grigoriev [4], Konstantin Yu. Zhizhin [1], Nikolay T. Kuznetsov [1]

1 N.S. Kurnakov Institute of General and Inorganic Chemistry of the Russian Academy of Sciences, Leninskii pr.31, Moscow 119991, Russia veravoinova@rx24.ru (V.V.V.); GooVee@yandex.ru (N.A.S.); bykov@igic.ras.ru (A.Y.B.);(I.N.K.); zhdanov@igic.ras.ru (A.P.Z.), zhizhin@igic.ras.ru (K.Y.Z.); ntkuz@igic.ras.ru (N.T.K.)

2 Institute for African Studies of the Russian Academy of Sciences (IAS), st. Spiridonovka 30/1, Moscow 123001, Russia, tyrishev@gmail.com (E.S.T.)

3 Lomonosov Moscow State University, Leninskie Gory 1, Moscow 119991, Russia, exsergion@gmail.com (S.S.N.)

4 Frumkin Institute of Physical Chemistry and Electrochemistry, Russian Academy of Sciences, Leninskii pr. 31, Bldg 4, 119071 Moscow, Russia; mickgrig@mail.ru (M.S.G.)

* Correspondence: zhdanov@igic.ras.ru; Tel.: +7-926-727-0139

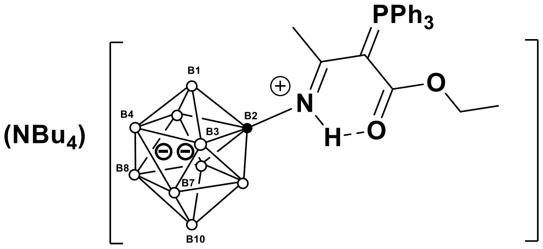

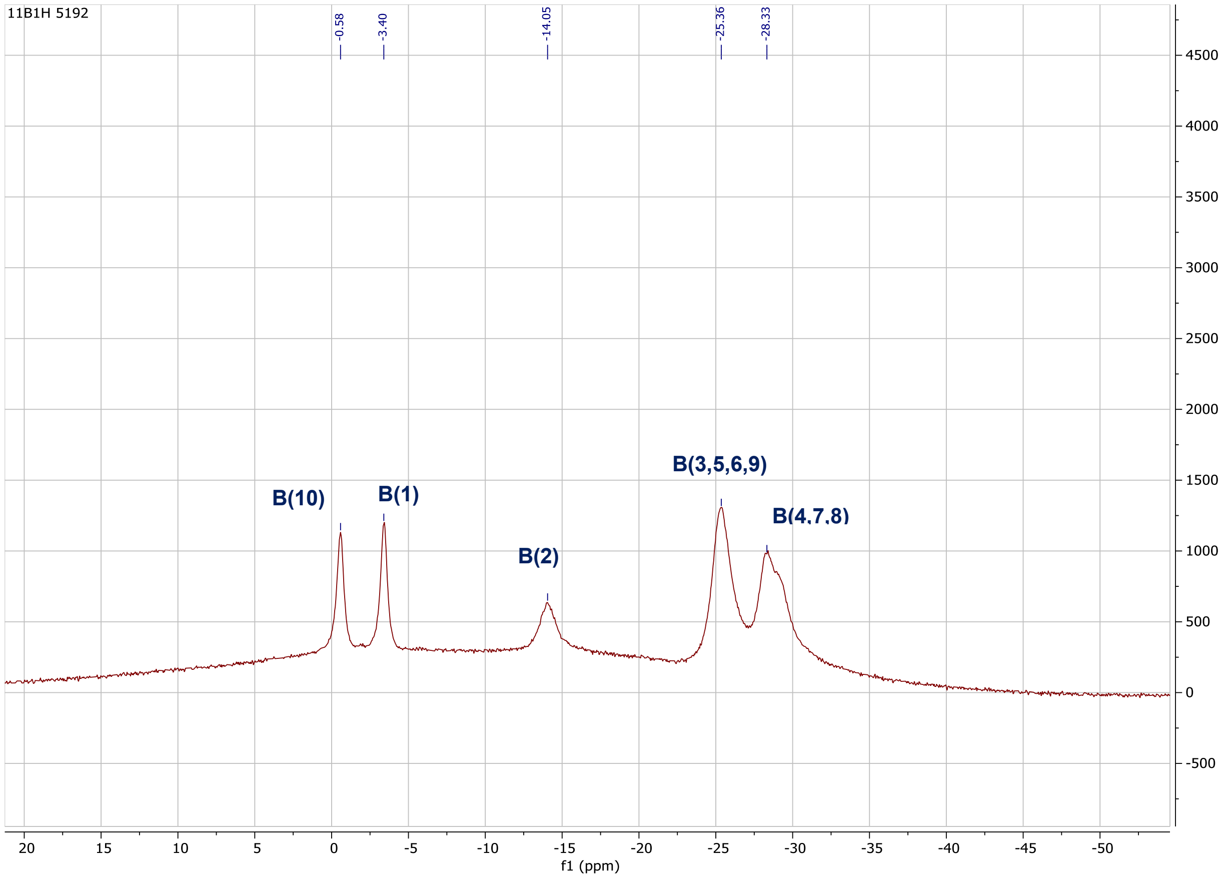

Figure S1. $^{11}B\{^{1}H\}$ **NMR spectrum of** $(Bu_4N)[2\text{-}B_{10}H_9NHC(Ph_3PCCOOEt)CH_3)]$ **(3a).**

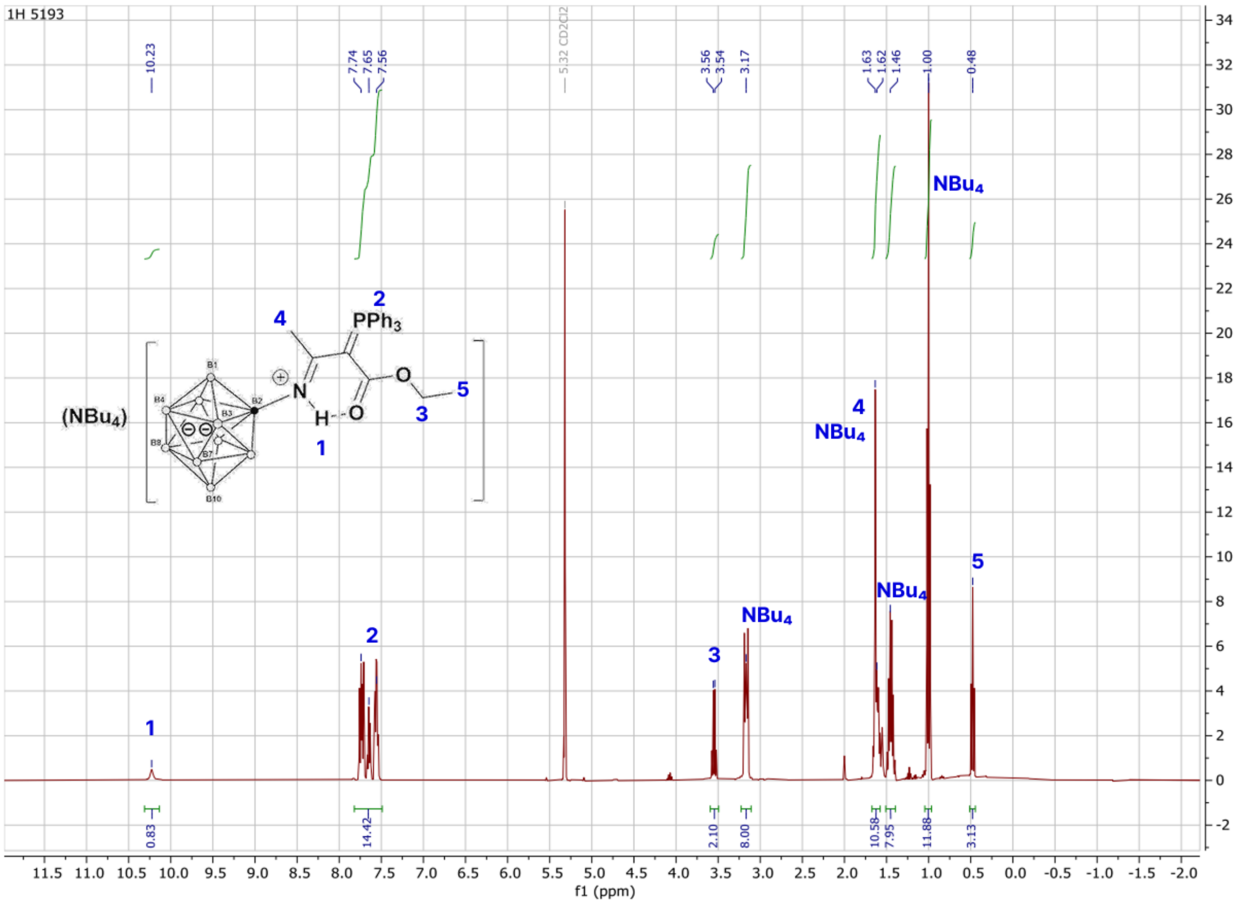

Figure S2. ^{1}H **NMR spectrum of** $(Bu_4N)[2\text{-}B_{10}H_9NHC(Ph_3PCCOOEt)CH_3)]$ **(3a).**

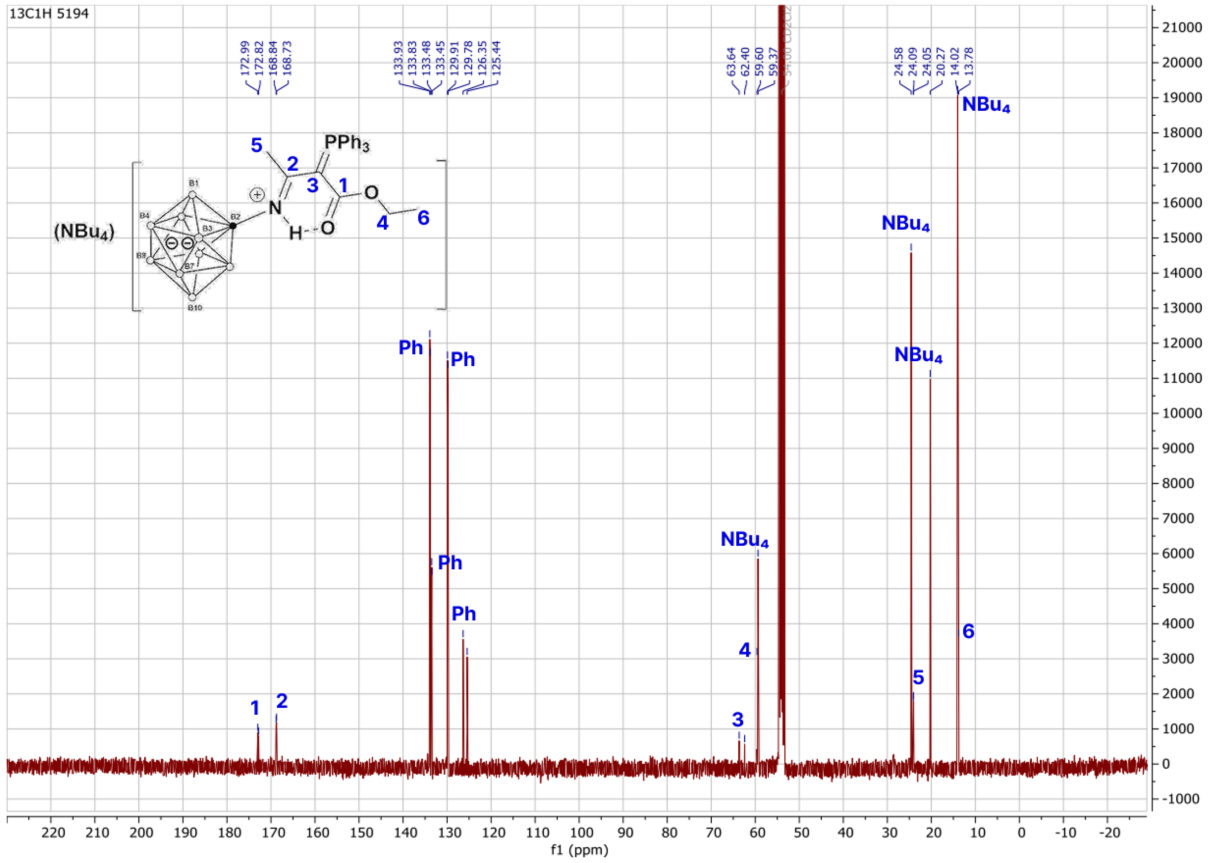

Figure S3. ^{13}C **NMR spectrum of** $(Bu_4N)[2\text{-}B_{10}H_9NHC(Ph_3PCCOOEt)CH_3)]$ **(3a).**

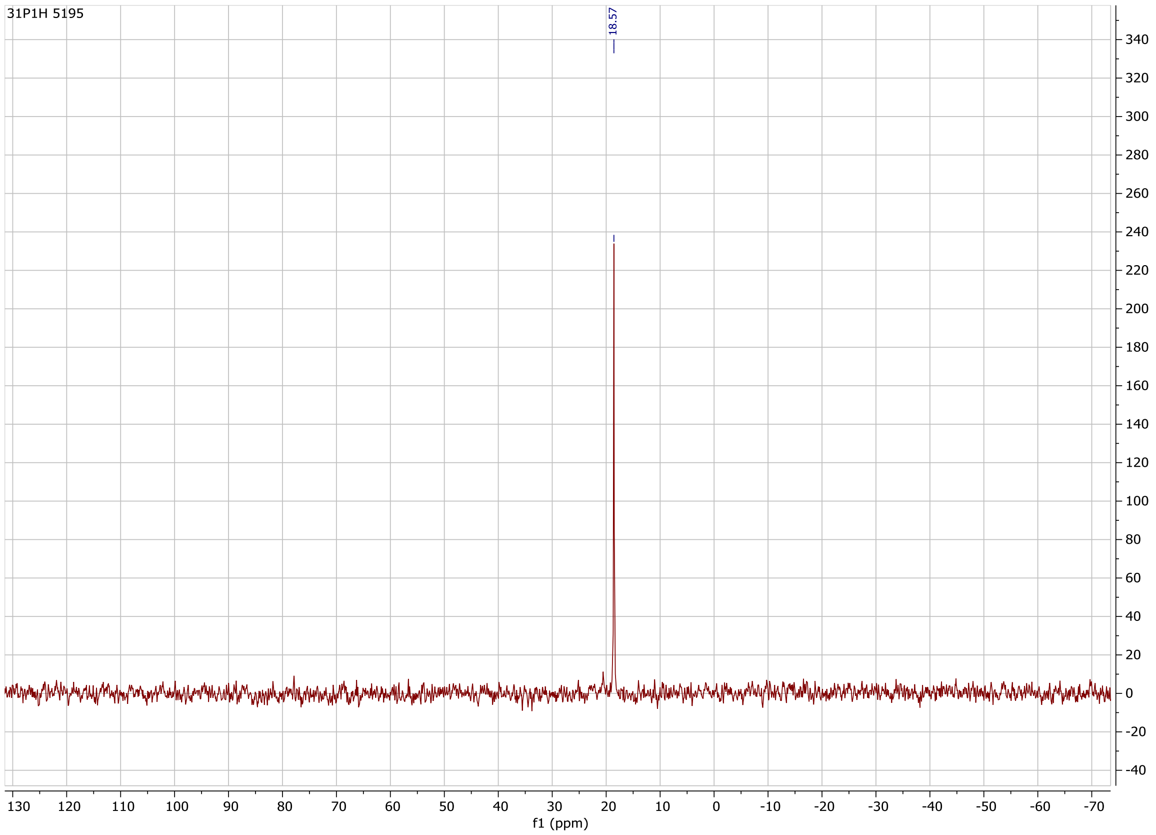

Figure S4. ^{31}P **NMR spectrum of** $(Bu_4N)[2\text{-}B_{10}H_9NHC(Ph_3PCCOOEt)CH_3)]$ **(3a).**

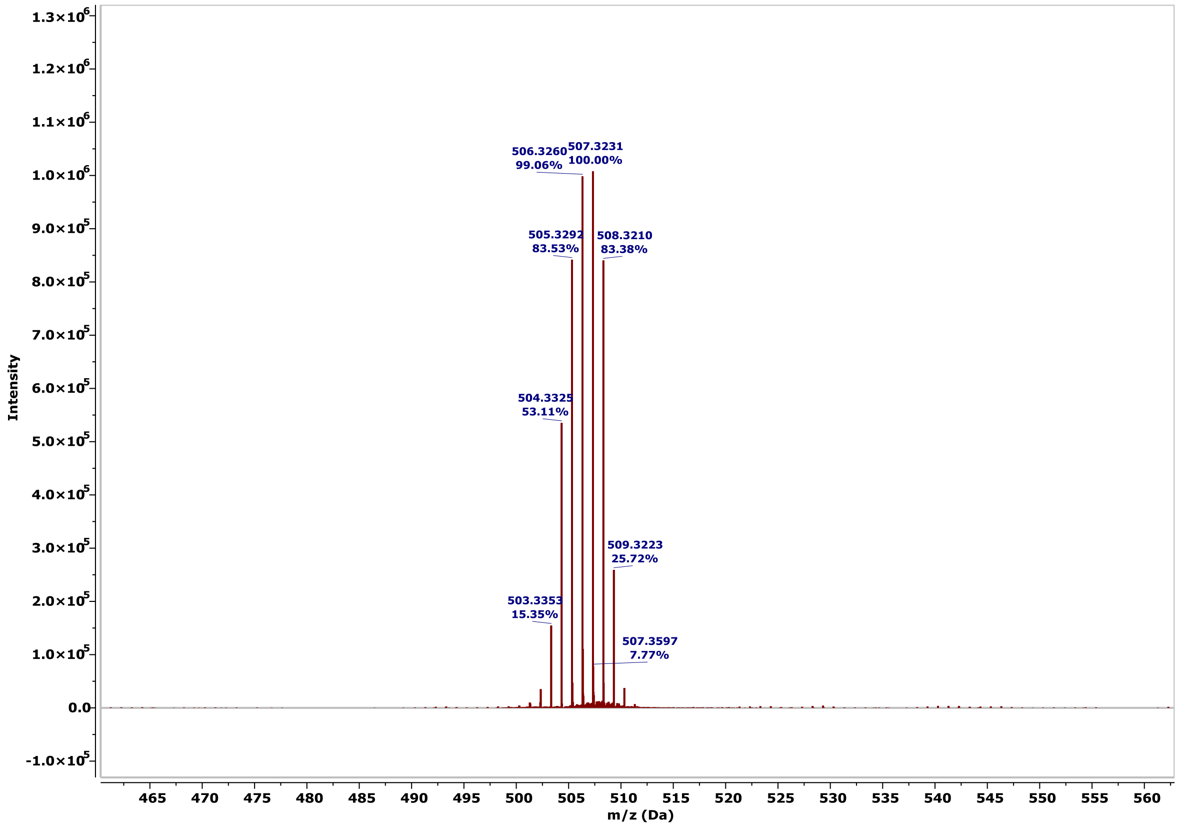

Figure S5. ESI-HRMS (negative area) spectrum of $(Bu_4N)[2\text{-}B_{10}H_9NHC(Ph_3PCCOOEt)CH_3)]$ **(3a).**

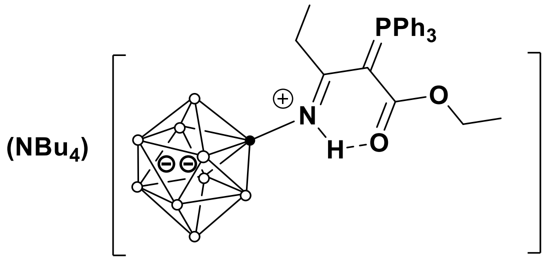

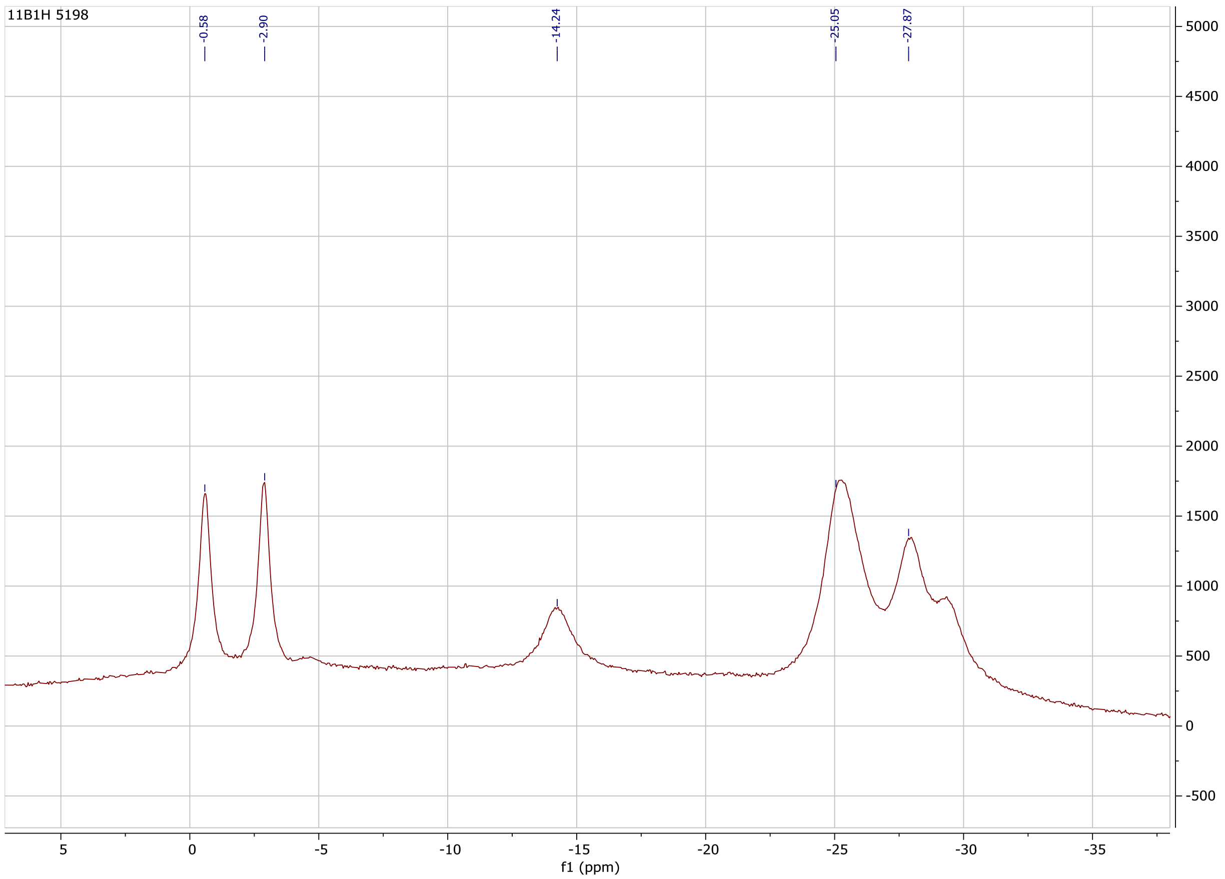

Figure S6. $^{11}B\{^{1}H\}$ **NMR spectrum of** $(Bu_4N)[2\text{-}B_{10}H_9NHC(Ph_3PCCOOEt)C_2H_5)]$ **(3b).**

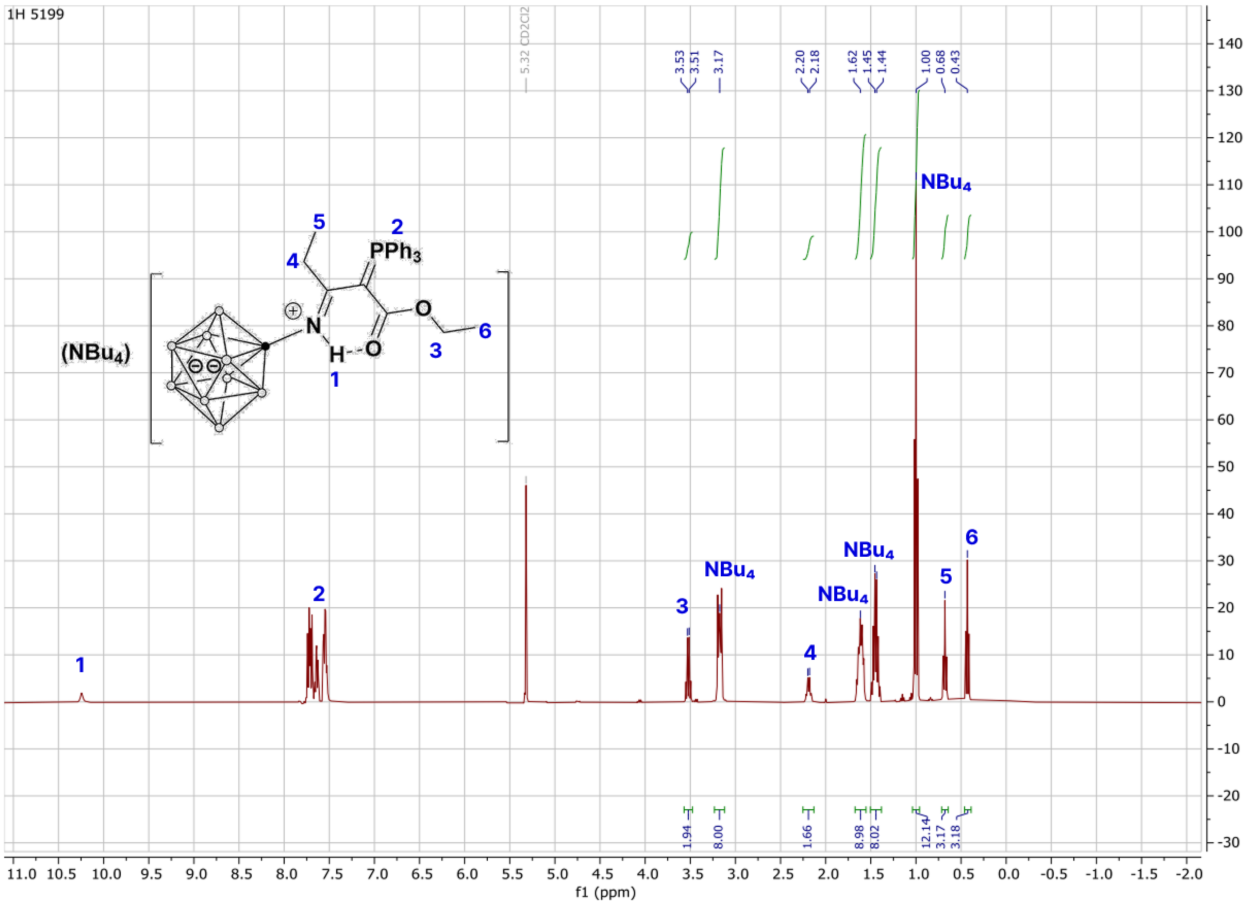

Figure S7. ^{1}H NMR spectrum of $(Bu_4N)[2\text{-}B_{10}H_9NHC(Ph_3PCCOOEt)C_2H_5)]$ (3b).

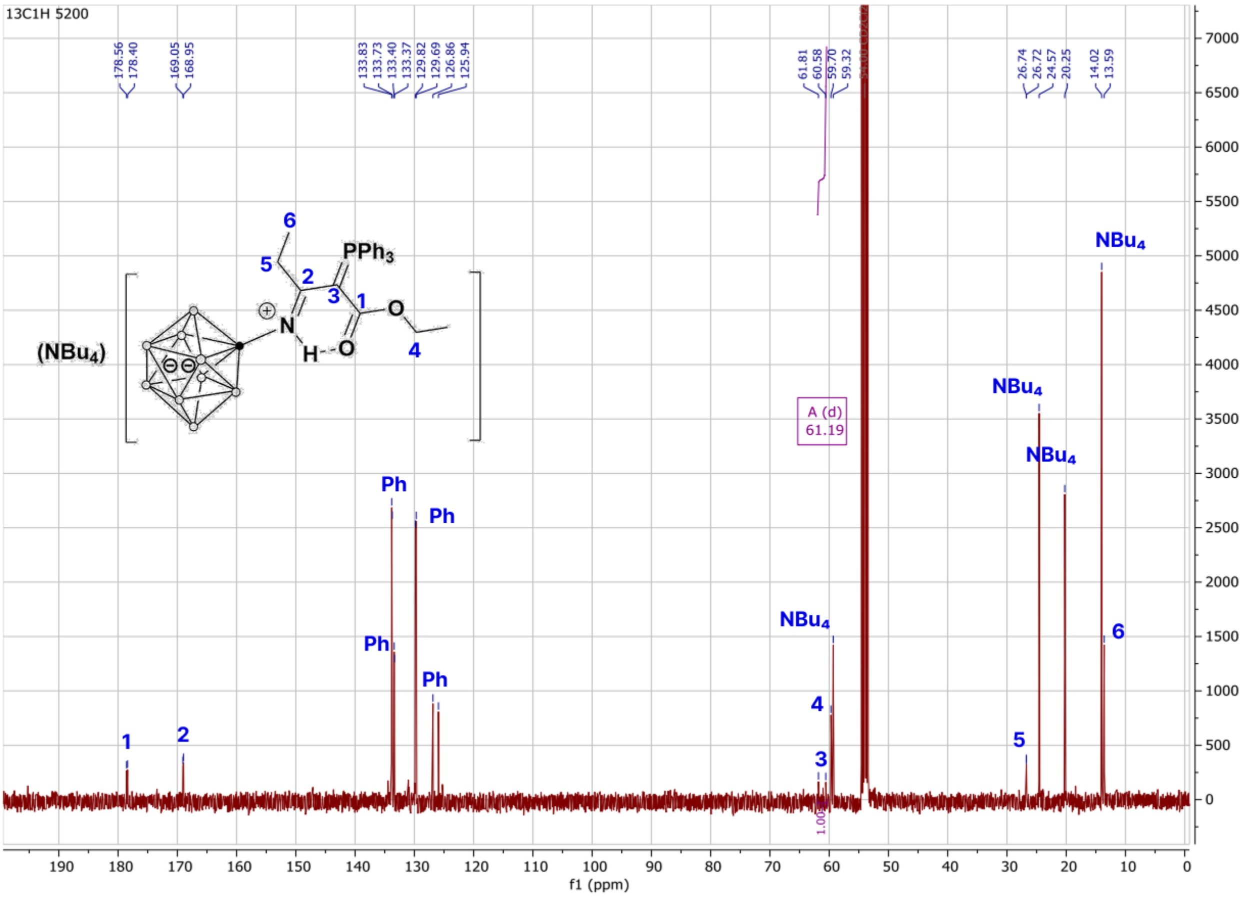

Figure S8. ^{13}C NMR spectrum of $(Bu_4N)[2\text{-}B_{10}H_9NHC(Ph_3PCCOOEt)C_2H_5)]$ (3b).

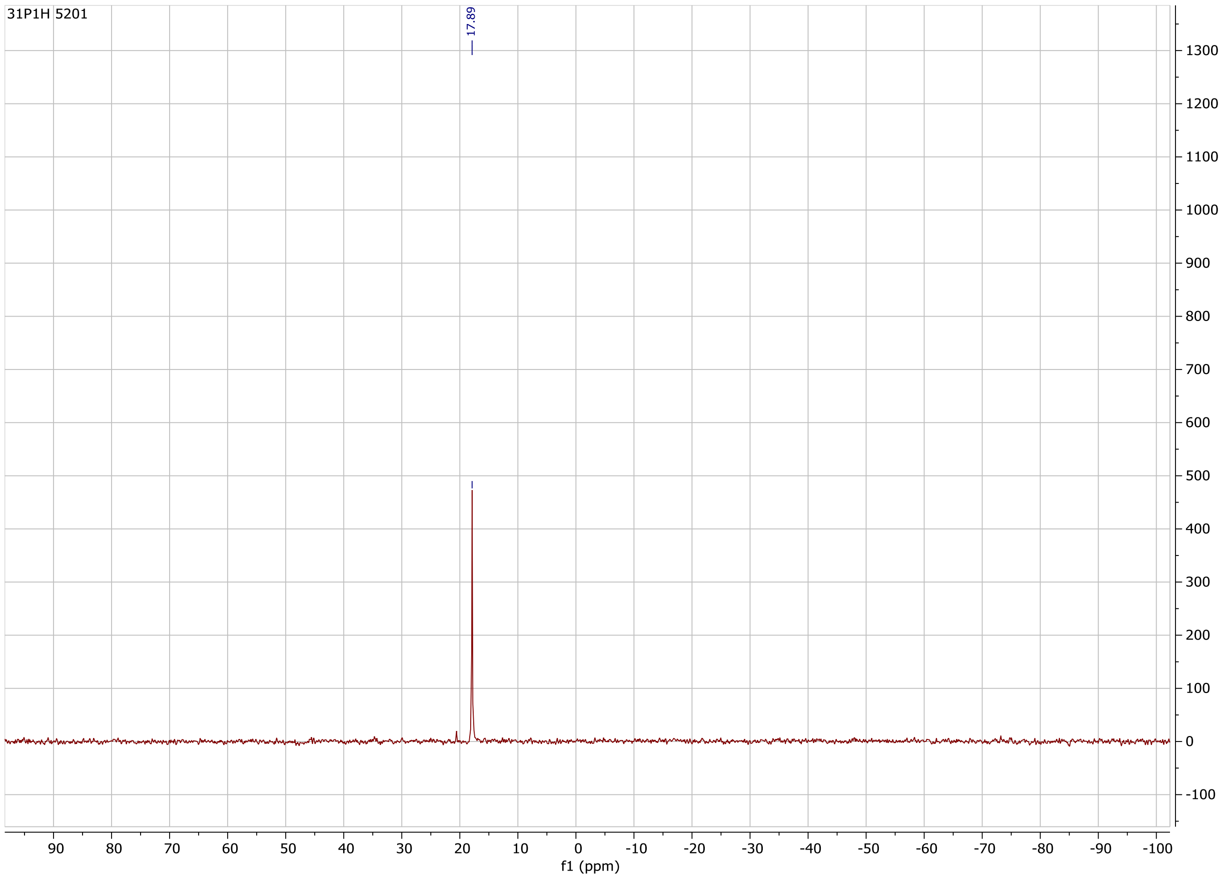

Figure S9. ^{31}**P NMR spectrum of** $(Bu_4N)[2\text{-}B_{10}H_9NHC(Ph_3PCCOOEt)C_2H_5)]$ **(3b).**

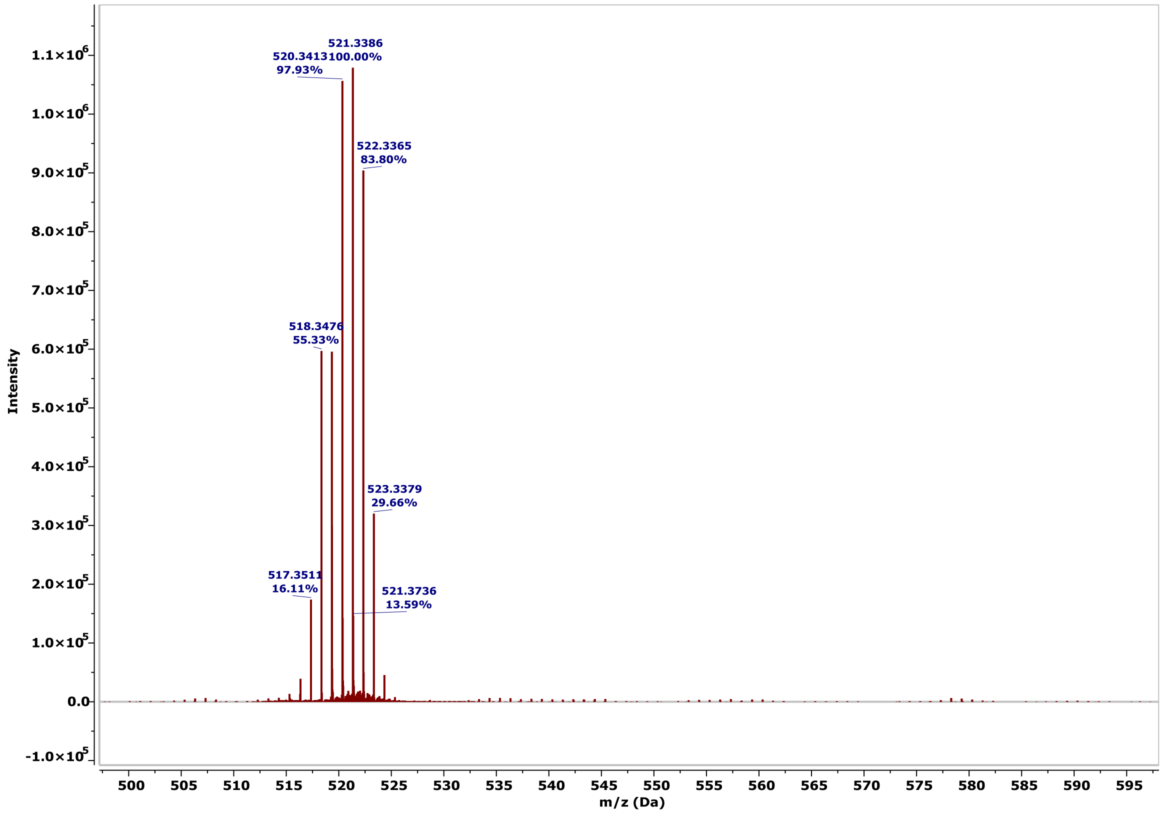

Figure S10. ESI-HRMS (negative area) spectrum of $(Bu_4N)[2\text{-}B_{10}H_9NHC(Ph_3PCCOOEt)C_2H_5)]$ **(3b).**

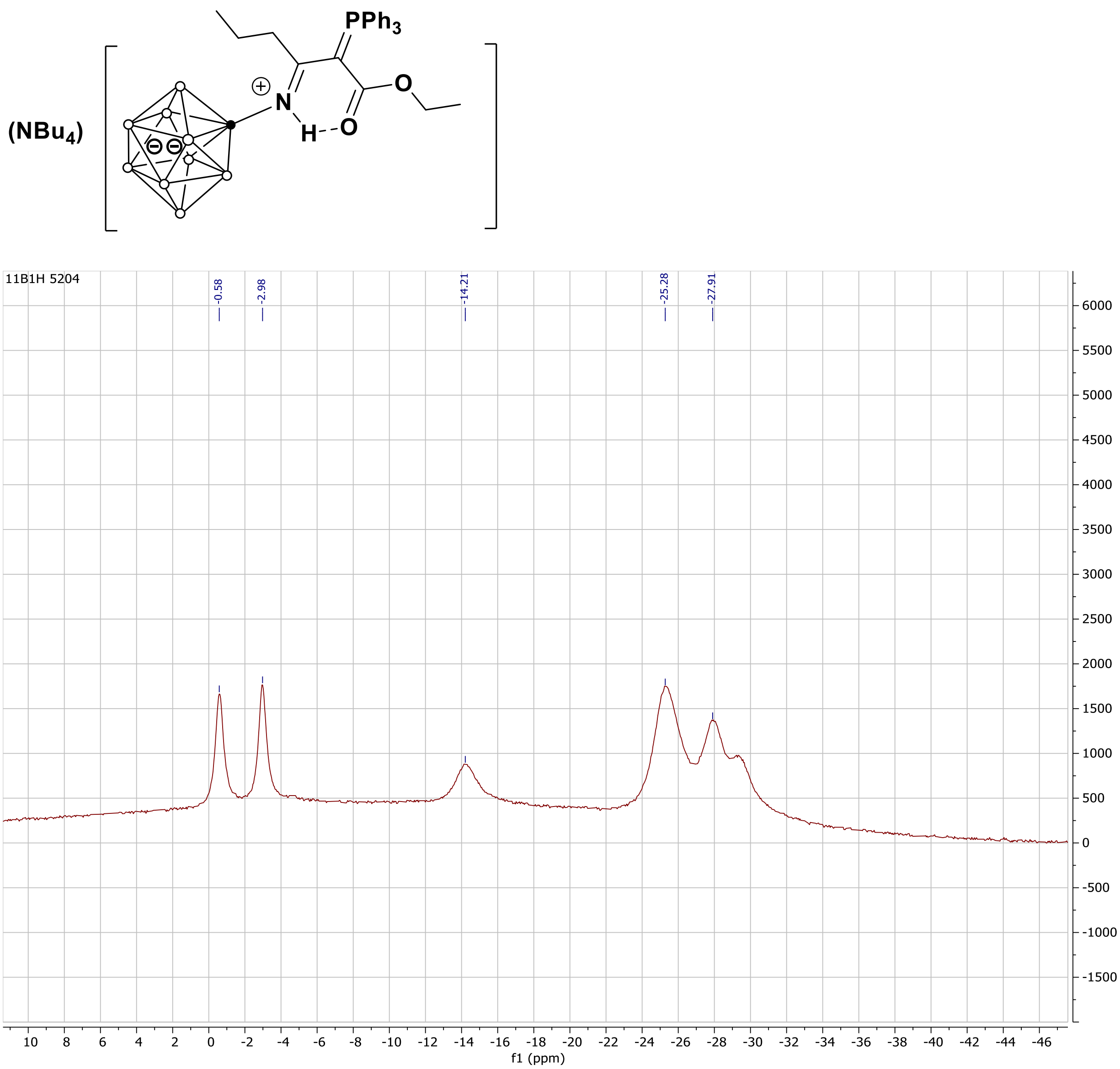

Figure S11. ^{11}**B{**^{1}**H} NMR spectrum of (Bu**$_4$**N)[2-B**$_{10}$**H**$_9$**NHC(Ph**$_3$**PCCOOEt)**^{n}**C**$_3$**H**$_7$**)] (3c).**

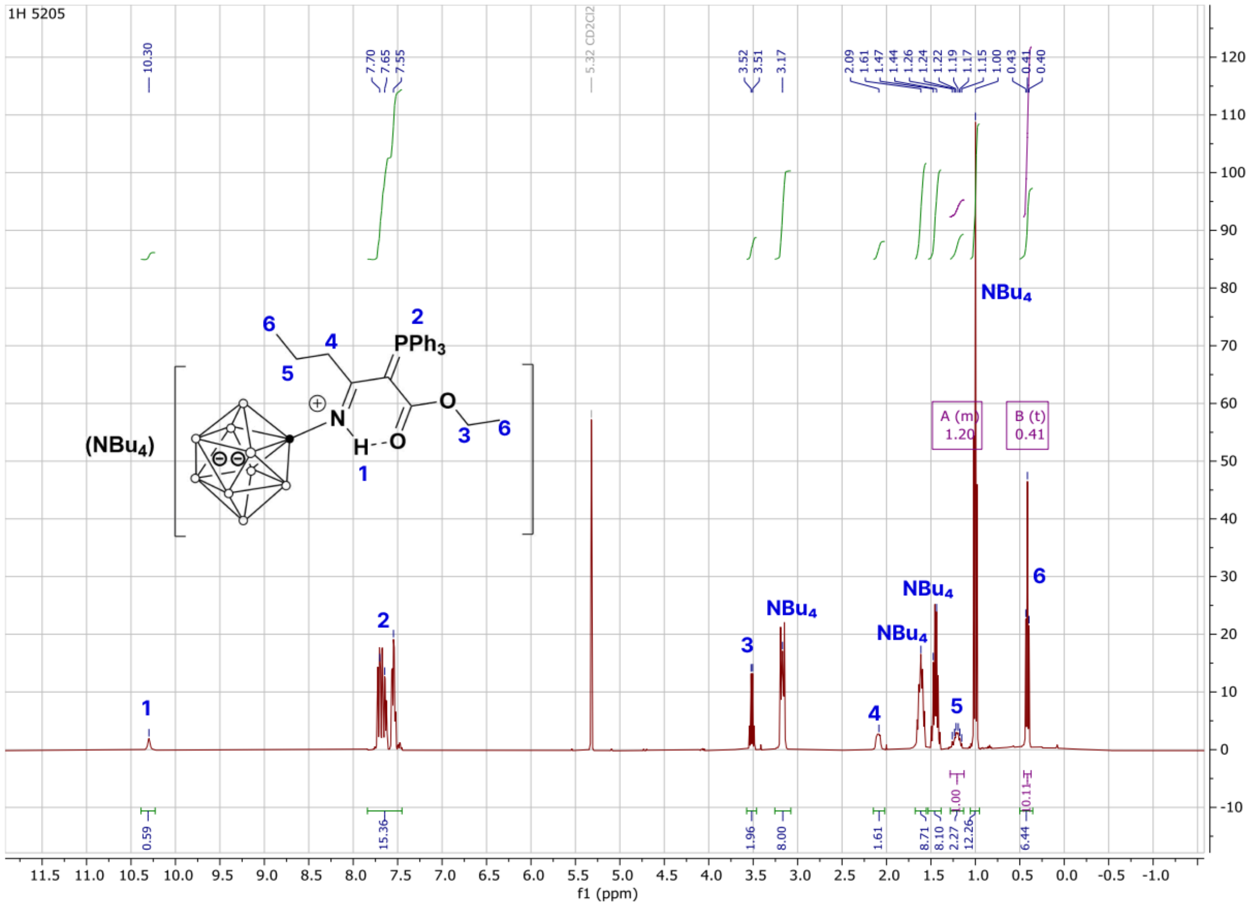

Figure S12. ^{1}H **NMR spectrum of** $(Bu_4N)[2\text{-}B_{10}H_9NHC(Ph_3PCCOOEt)^nC_3H_7)]$ **(3c).**

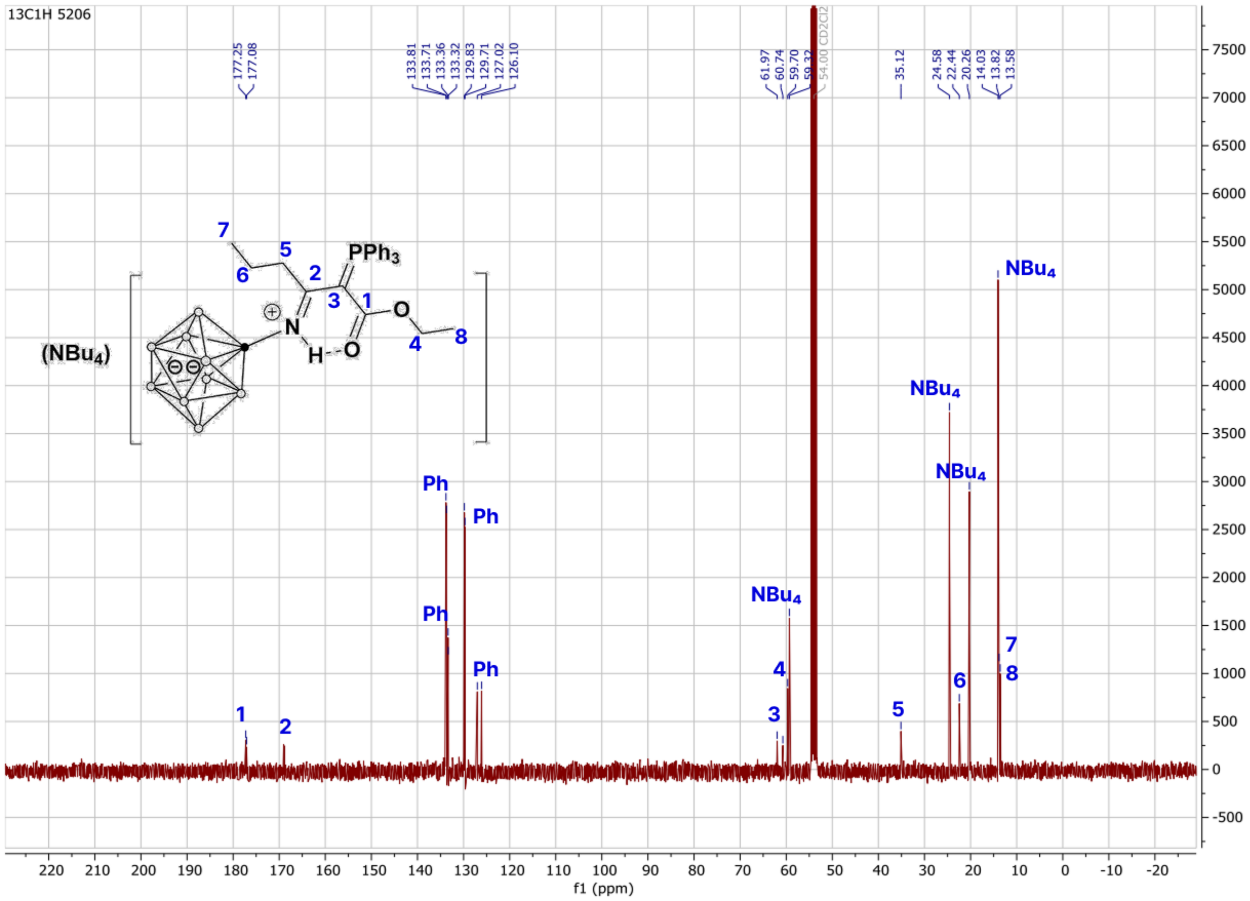

Figure S13. ^{13}C **NMR spectrum of** $(Bu_4N)[2\text{-}B_{10}H_9NHC(Ph_3PCCOOEt)^nC_3H_7)]$ **(3c).**

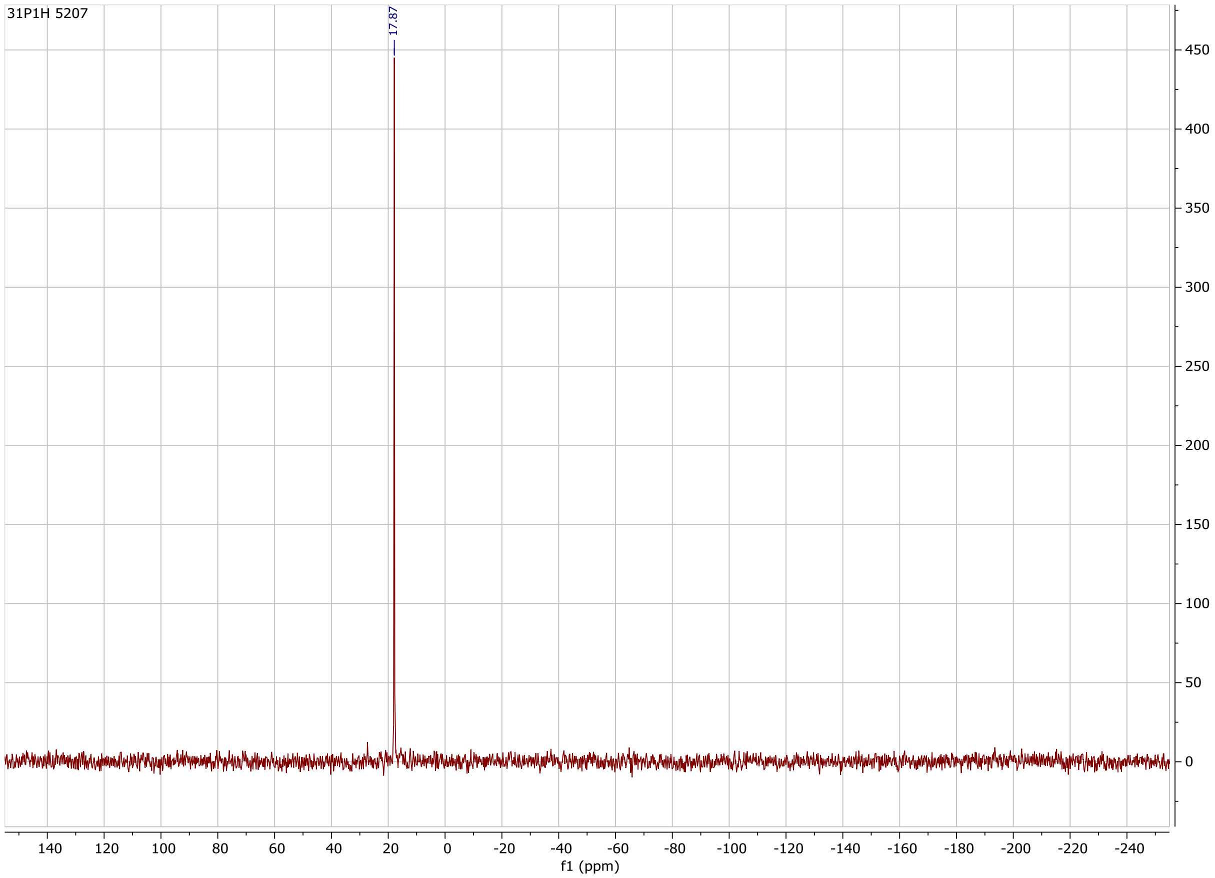

Figure S14. ^{31}**P NMR spectrum of** $(Bu_4N)[2\text{-}B_{10}H_9NHC(Ph_3PCCOOEt)^nC_3H_7)]$ **(3c).**

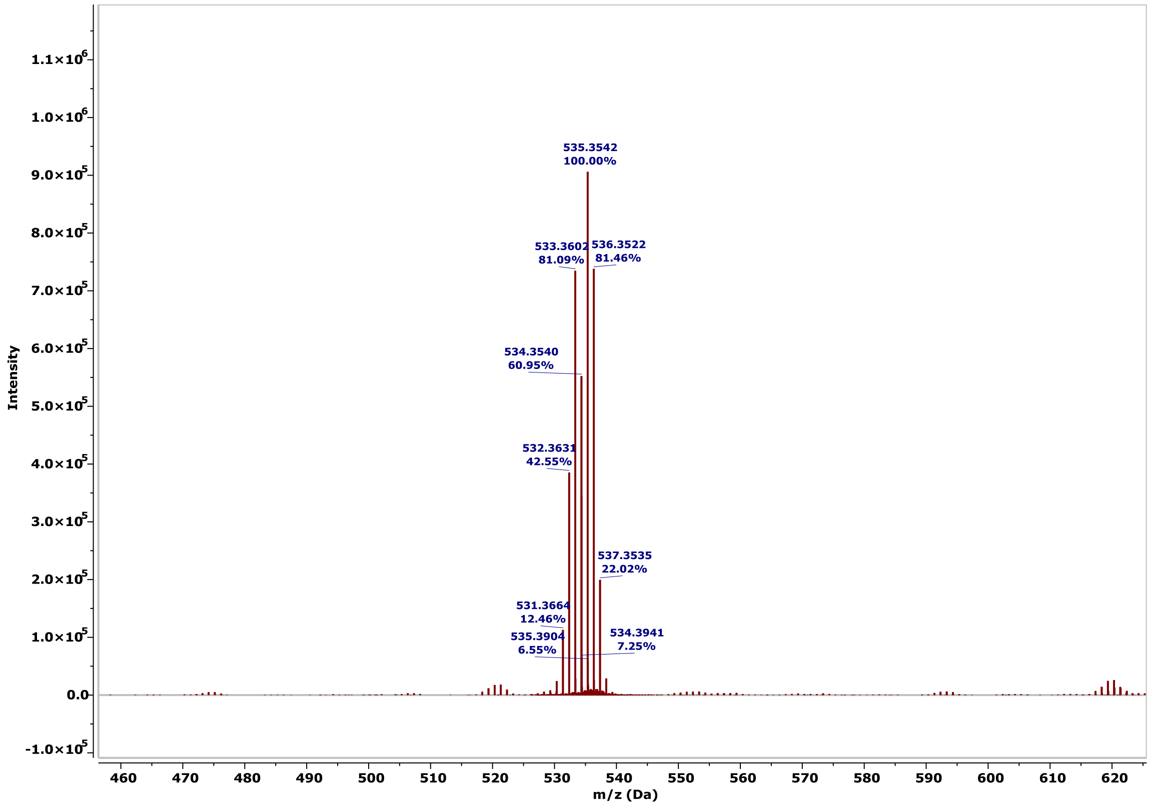

Figure S15. ESI-HRMS (negative area) spectrum of $(Bu_4N)[2\text{-}B_{10}H_9NHC(Ph_3PCCOOEt)^nC_3H_7)]$ **(3c).**

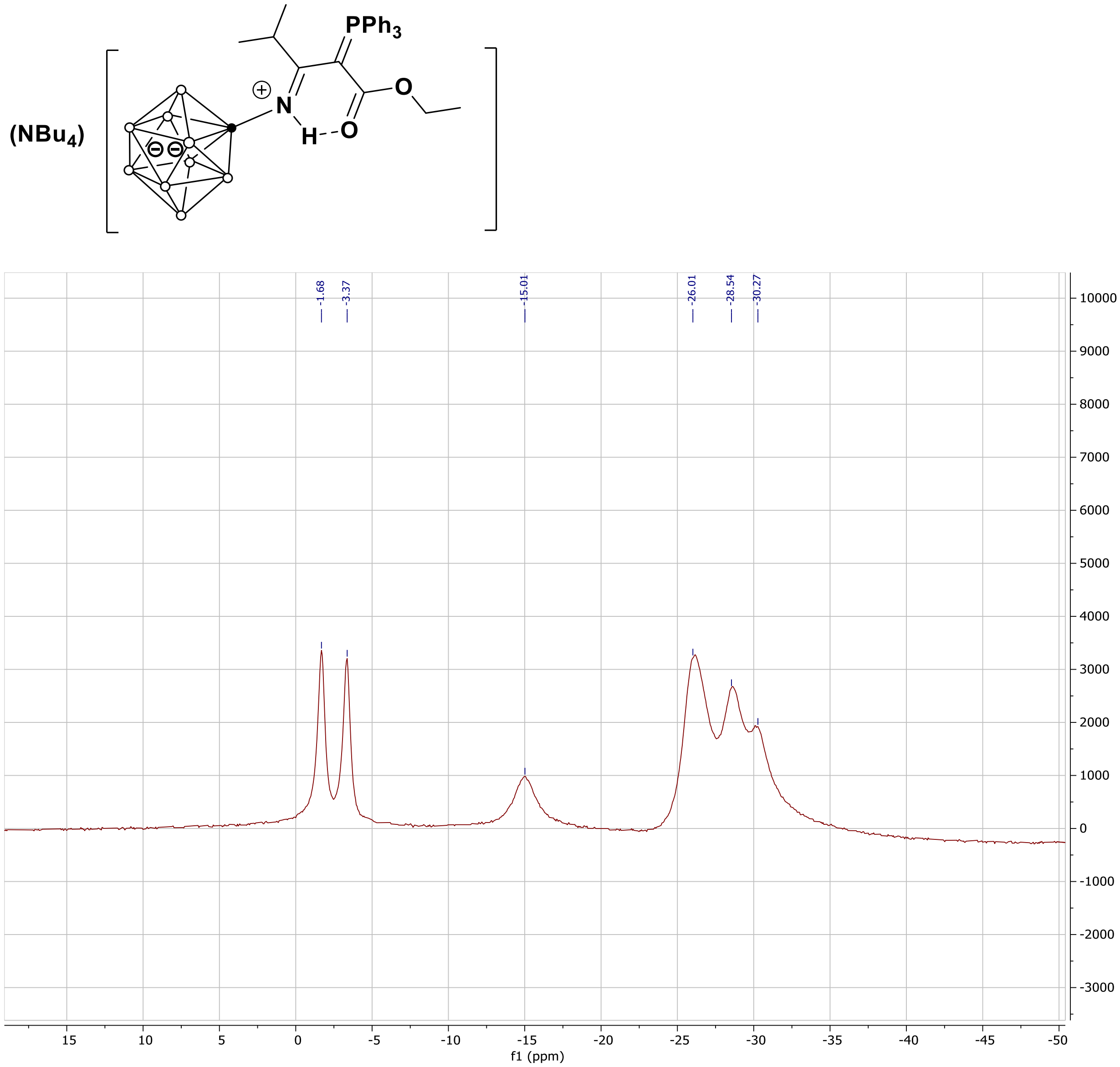

Figure S16. $^{11}B\{^{1}H\}$ **NMR spectrum of** $(Bu_4N)[2\text{-}B_{10}H_9NHC(Ph_3PCCOOEt)^{i}C_3H_7)]$ **(3d)**

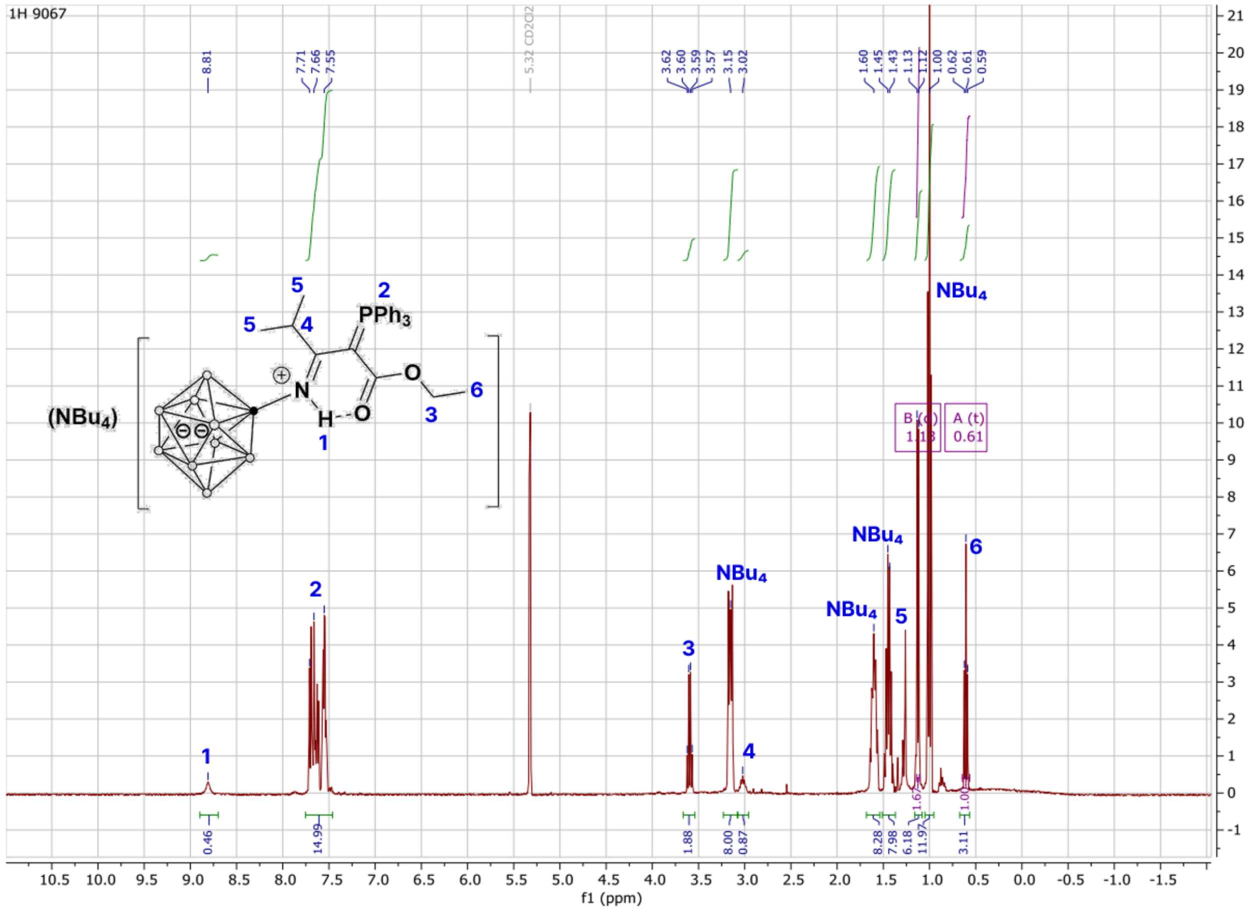

Figure S17. ^{1}H NMR spectrum of $(Bu_4N)[2\text{-}B_{10}H_9NHC(Ph_3PCCOOEt)^{i}C_3H_7)]$ (3d)

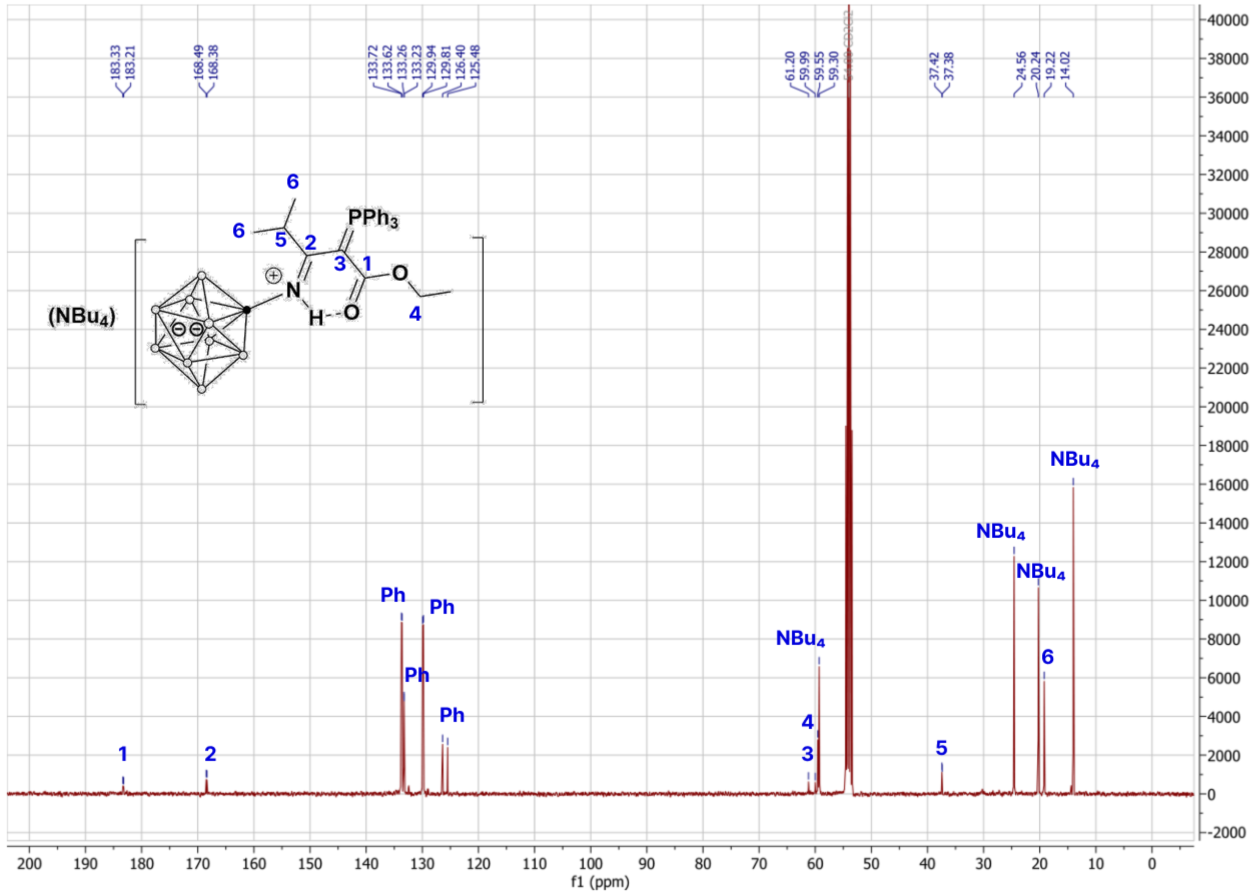

Figure S18. ^{13}C NMR spectrum of $(Bu_4N)[2\text{-}B_{10}H_9NHC(Ph_3PCCOOEt)^{i}C_3H_7)]$ (3d)

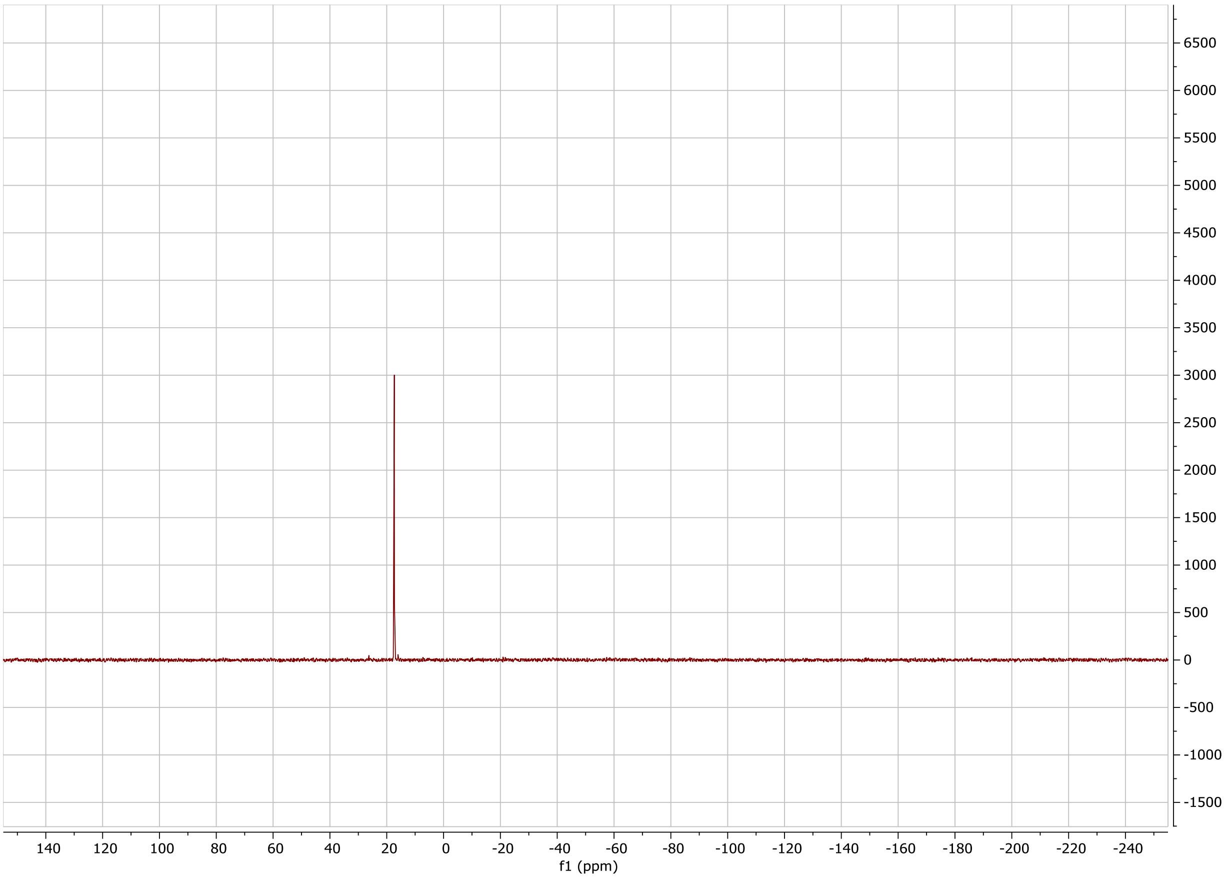

Figure S19. ^{31}**P NMR spectrum of** $(Bu_4N)[2\text{-}B_{10}H_9NHC(Ph_3PCCOOEt)^iC_3H_7)]$ **(3d)**

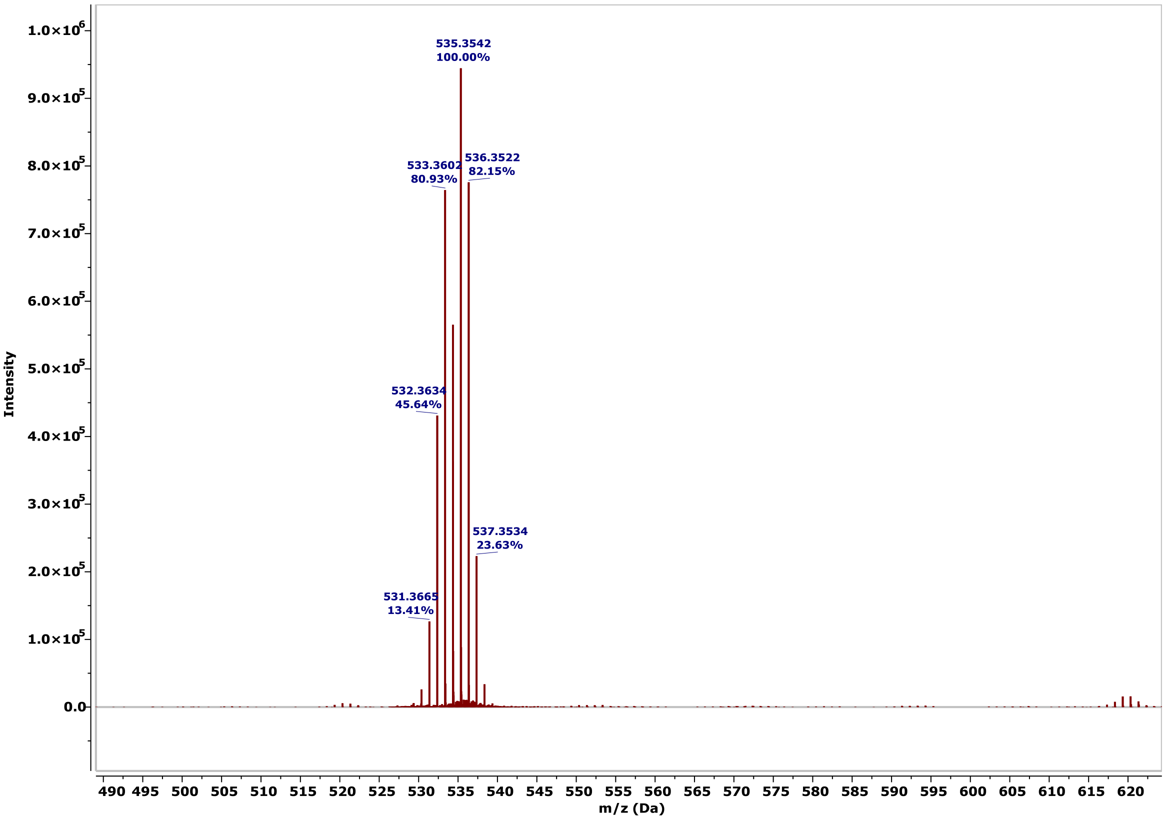

Figure S20. ESI-HRMS (negative area) spectrum of $(Bu_4N)[2\text{-}B_{10}H_9NHC(Ph_3PCCOOEt)^iC_3H_7)]$ **(3d)**

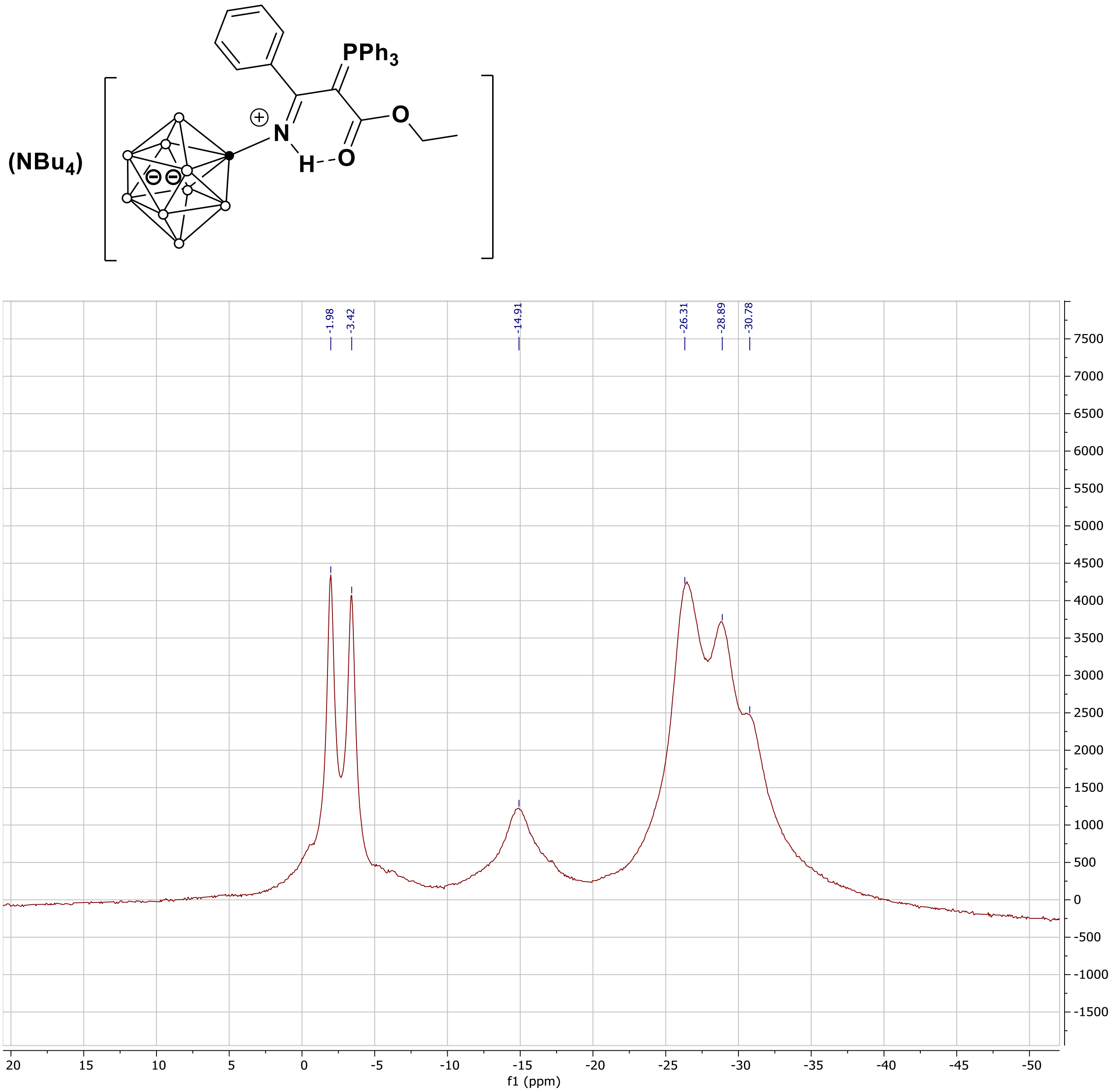

Figure S21. $^{11}B\{^{1}H\}$ **NMR spectrum of** $(Bu_4N)[2\text{-}B_{10}H_9NHC(Ph_3PCCOOEt)C_6H_5)]$ **(3e).**

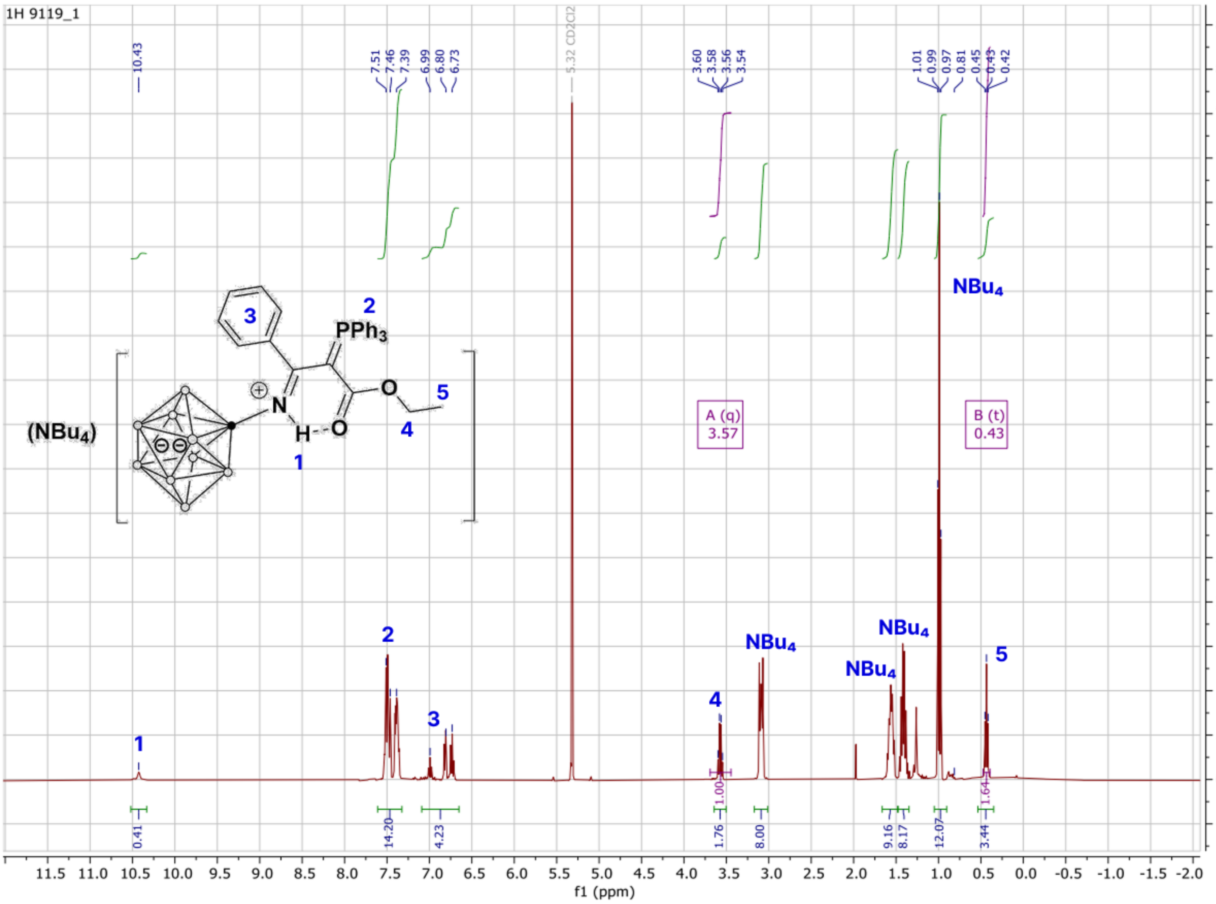

Figure S22. ^{1}H NMR spectrum of $(Bu_4N)[2\text{-}B_{10}H_9NHC(Ph_3PCCOOEt)C_6H_5)]$ (3e).

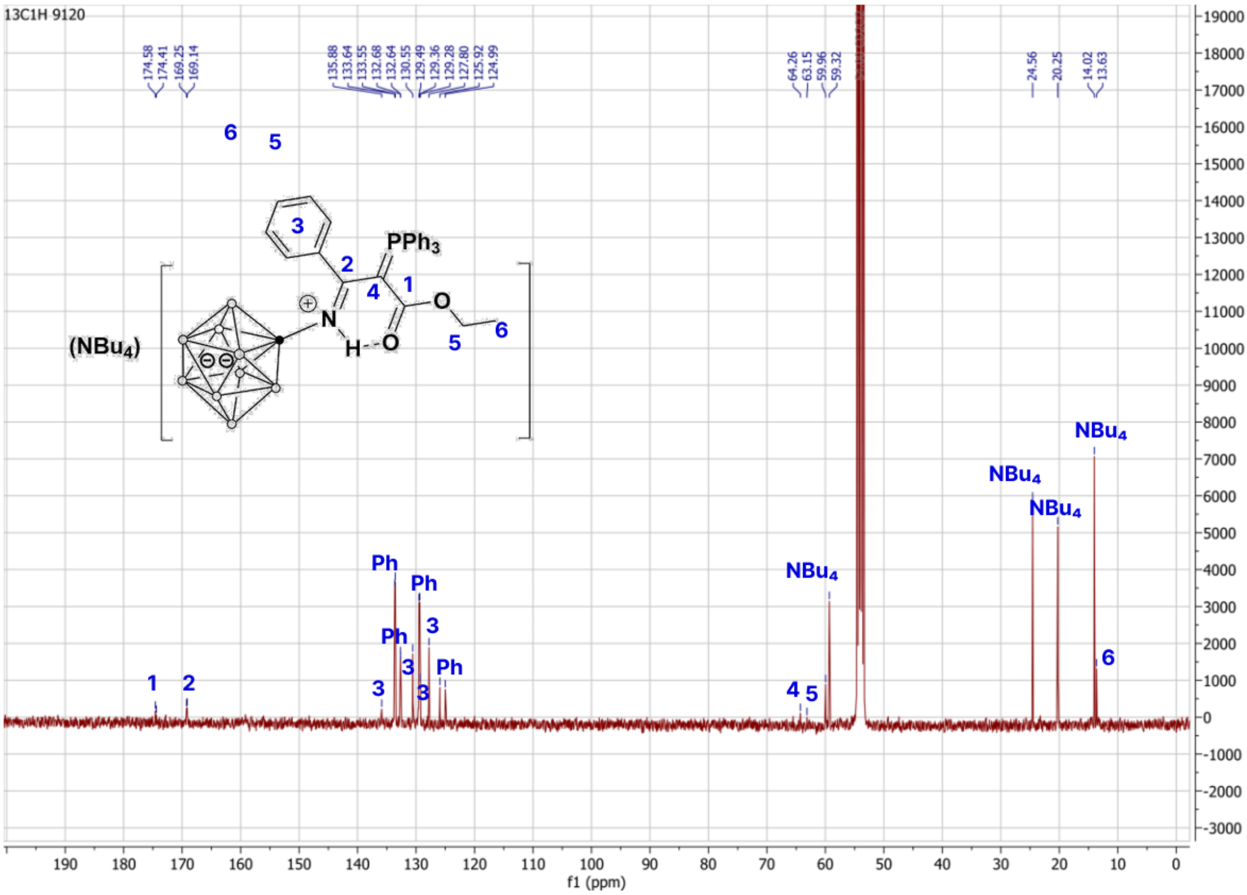

Figure S23. ^{13}C NMR spectrum of $(Bu_4N)[2\text{-}B_{10}H_9NHC(Ph_3PCCOOEt)C_6H_5)]$ (3e).

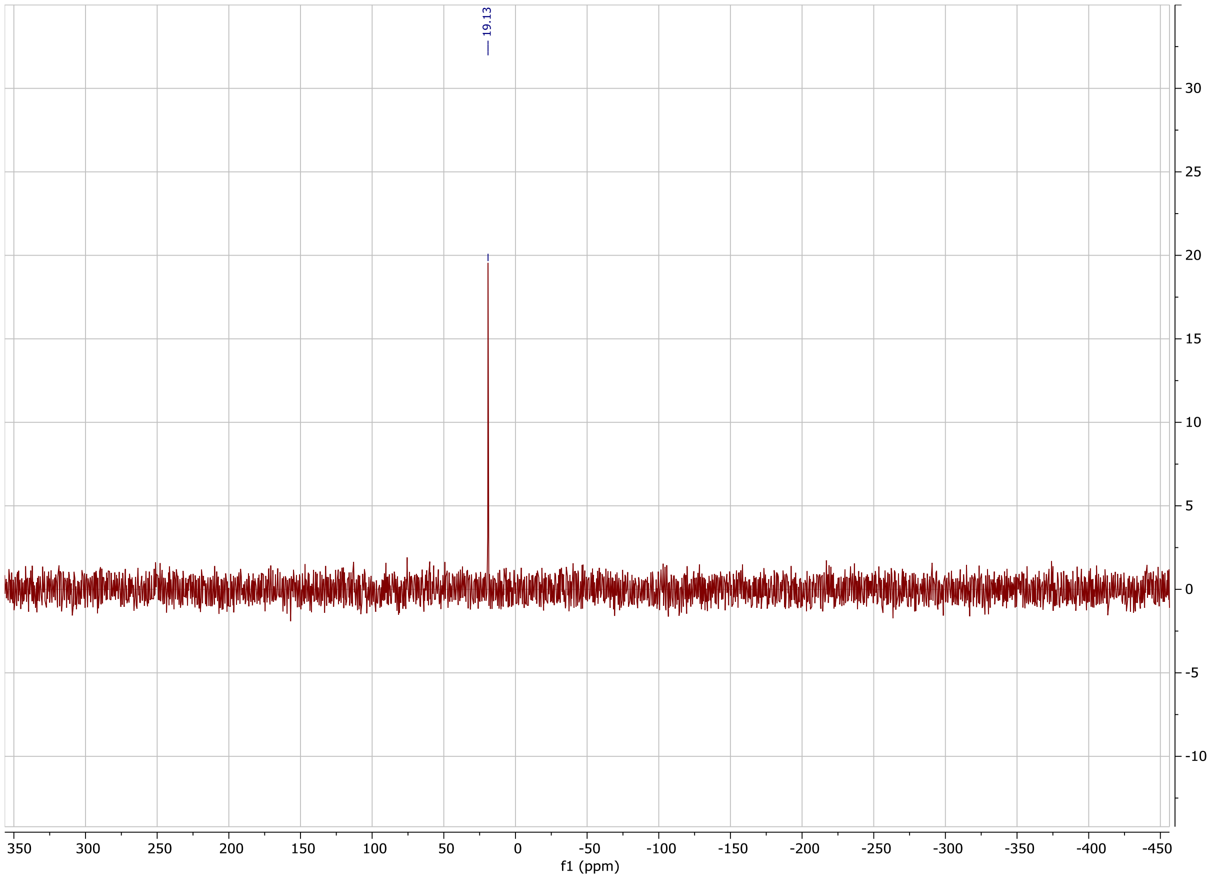

Figure S24. ^{31}**P NMR spectrum of** $(Bu_4N)[2\text{-}B_{10}H_9NHC(Ph_3PCCOOEt)C_6H_5)]$ **(3e).**

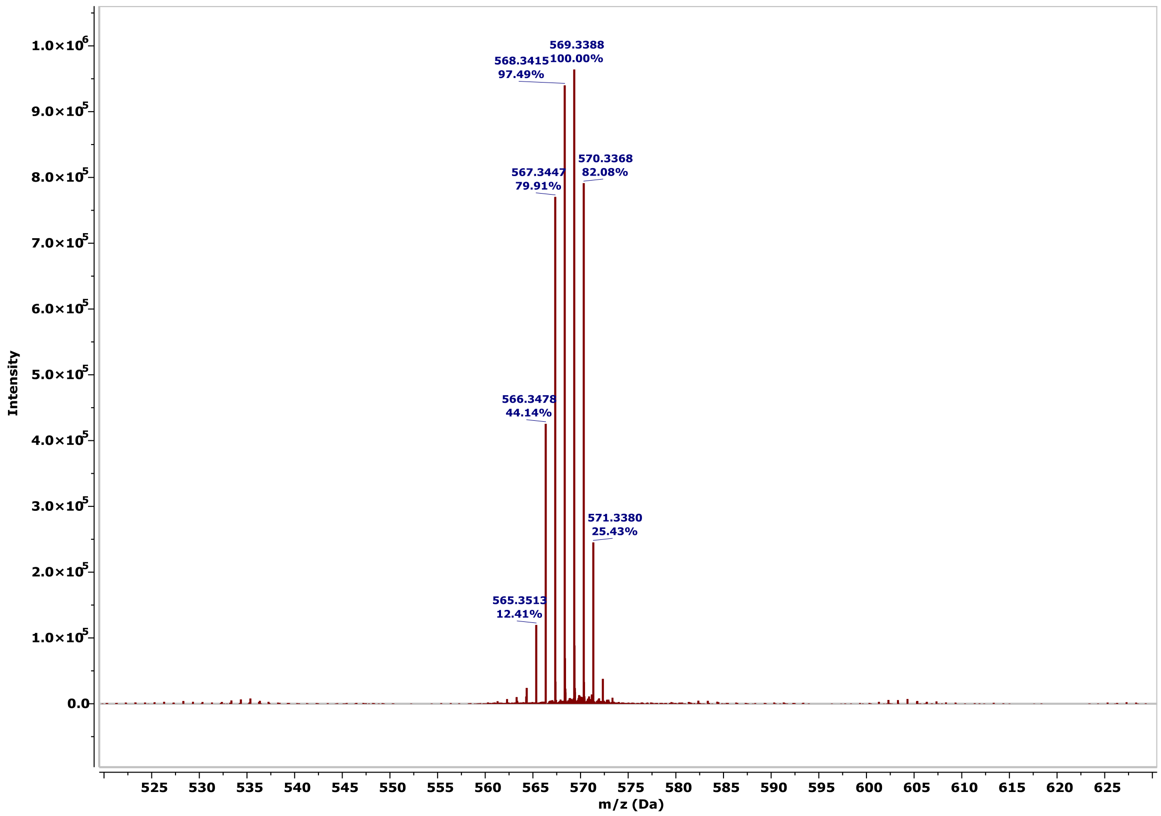

Figure S25. ESI-HRMS (negative area) spectrum of $(Bu_4N)[2\text{-}B_{10}H_9NHC(Ph_3PCCOOEt)C_6H_5)]$ **(3e).**

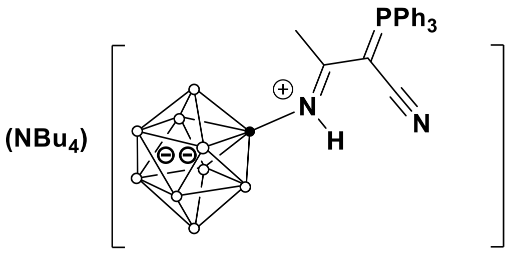

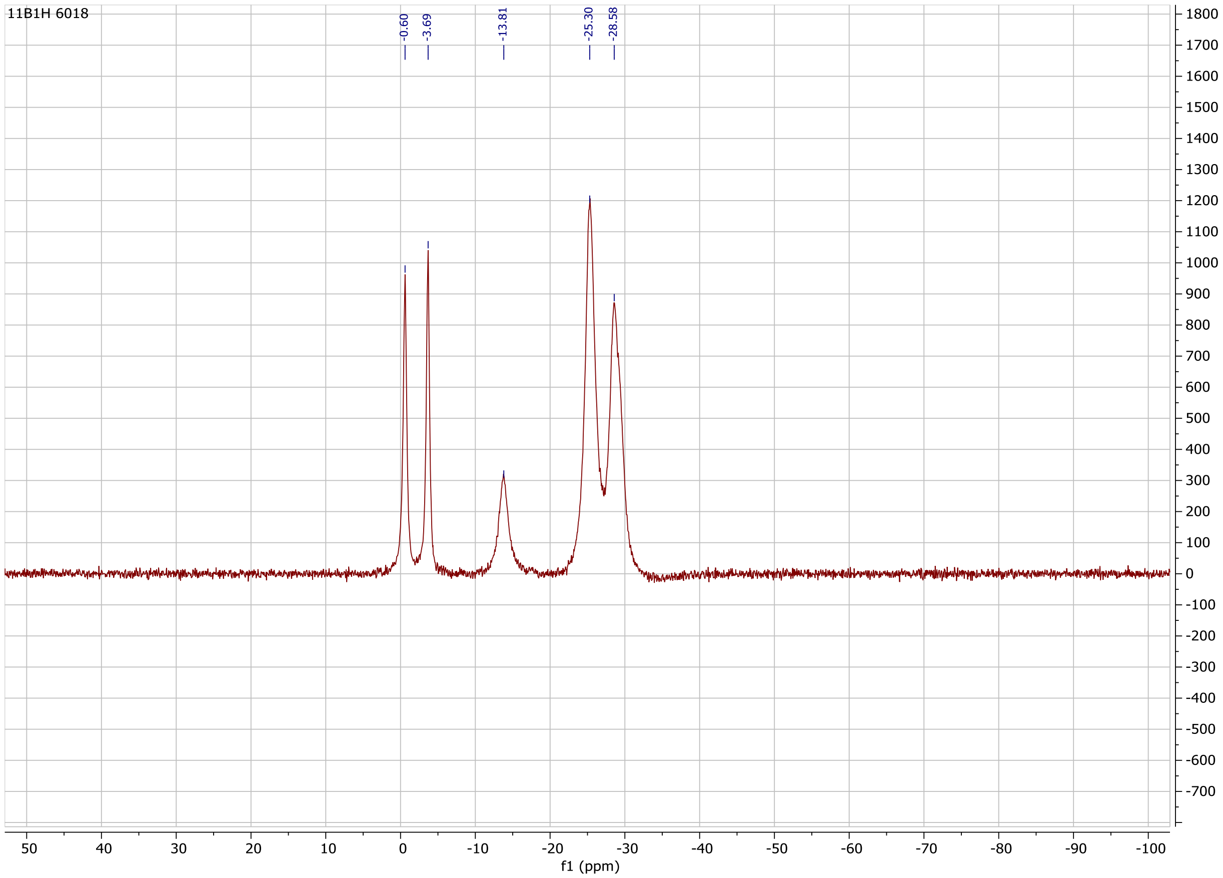

Figure S26. $^{11}B\{^1H\}$ **NMR spectrum of (Bu$_4$N)[2-B$_{10}$H$_9$NHC(Ph$_3$PCCN)CH$_3$)] (4a)**

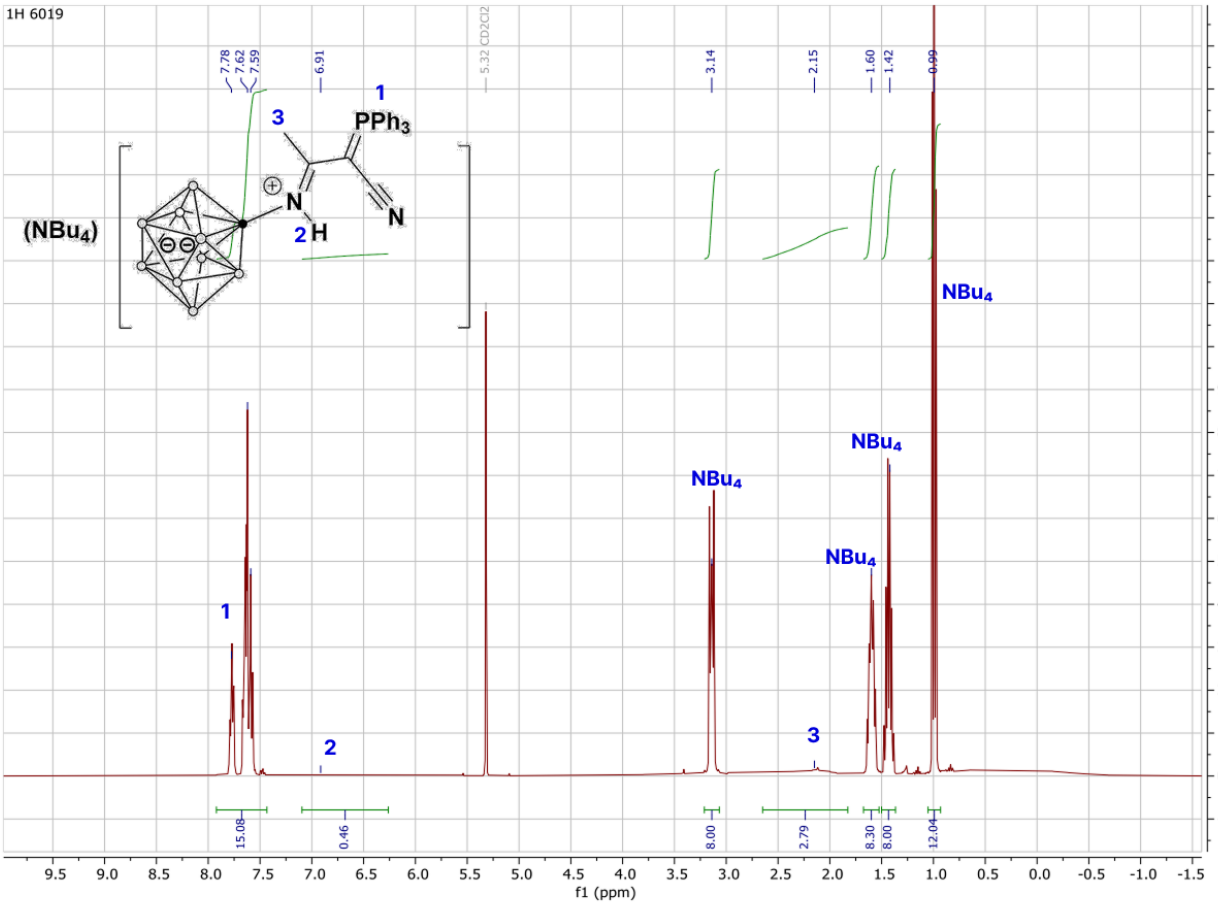

Figure S27. ^{1}H **NMR spectrum of** $(Bu_4N)[2\text{-}B_{10}H_9NHC(Ph_3PCCN)CH_3)]$ **(4a)**

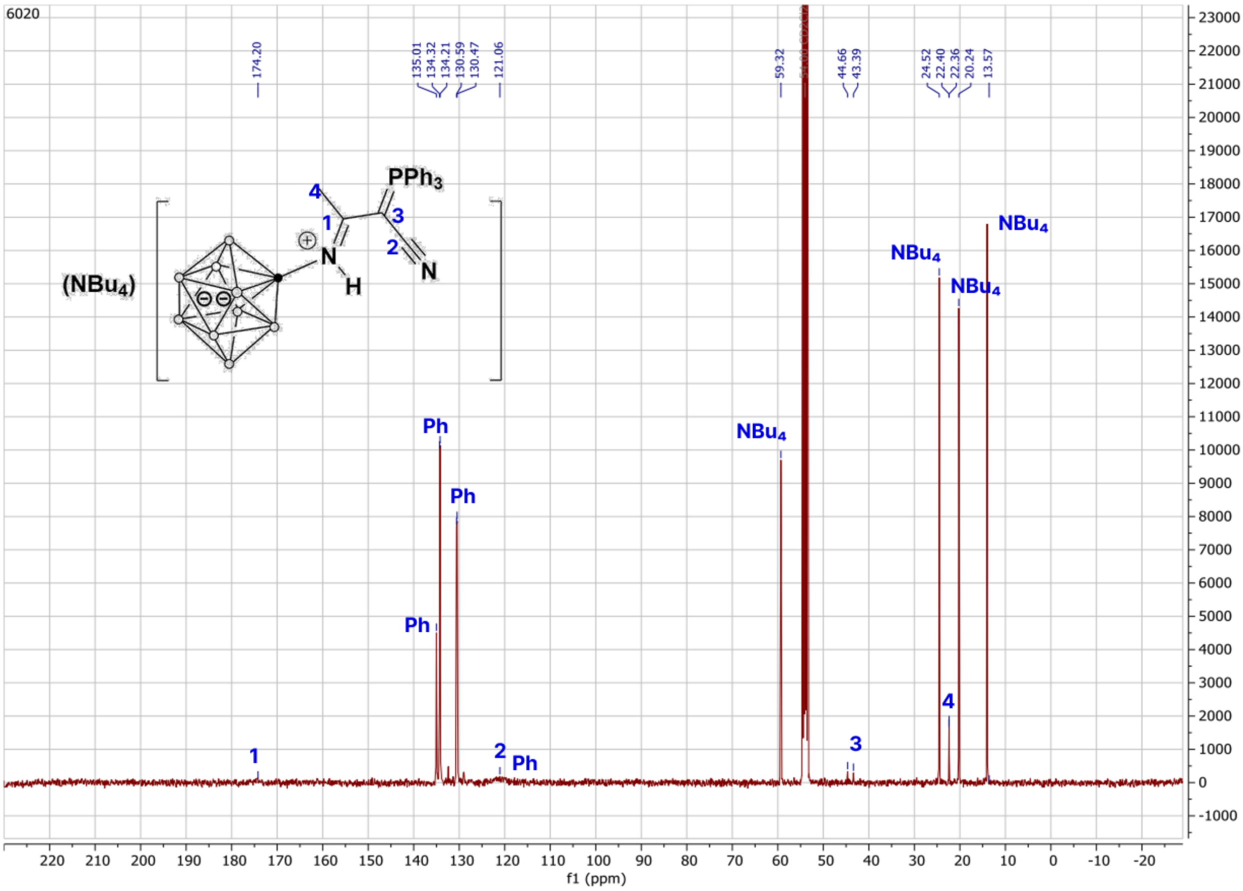

Figure S28. ^{13}C **NMR spectrum of** $(Bu_4N)[2\text{-}B_{10}H_9NHC(Ph_3PCCN)CH_3)]$ **(4a)**

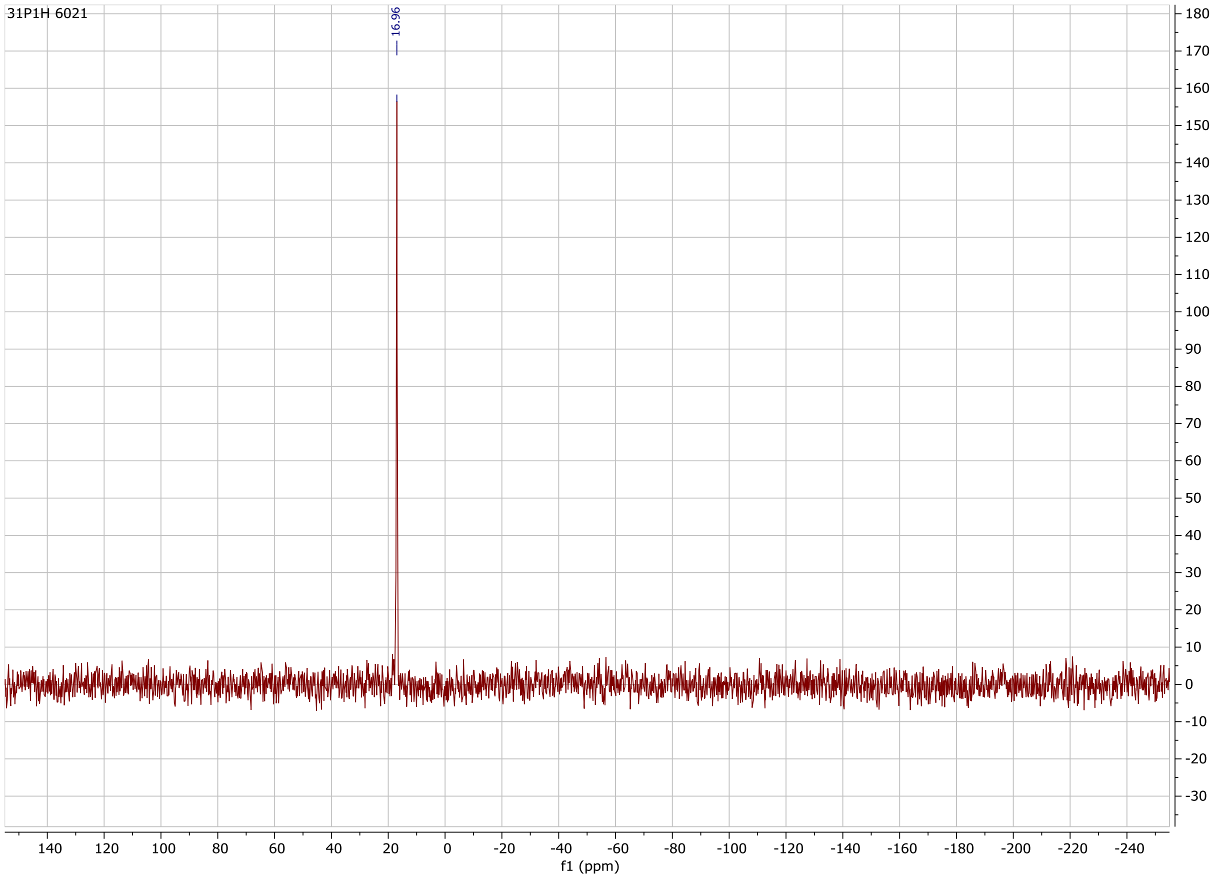

Figure S29. ^{31}P **NMR spectrum of** $(Bu_4N)[2-B_{10}H_9NHC(Ph_3PCCN)CH_3)]$ **(4a)**

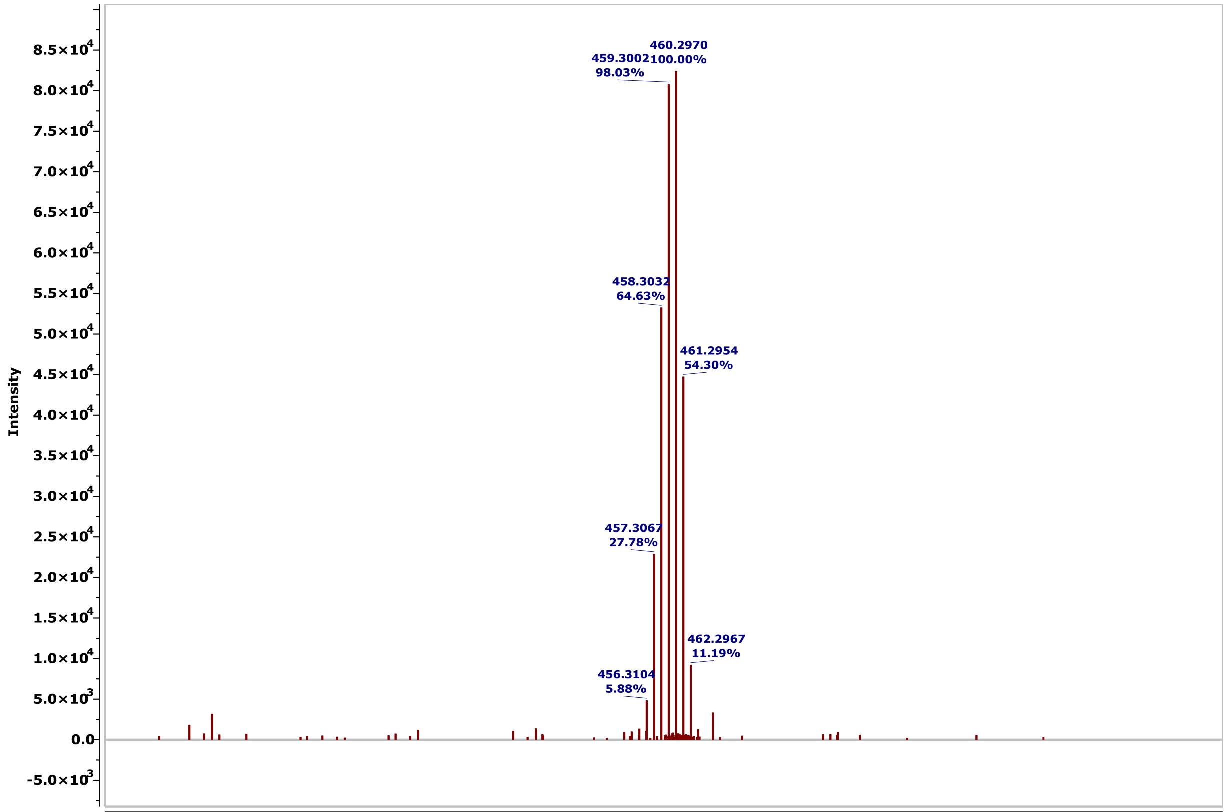

Figure S30. ESI-HRMS (negative area) spectrum of $(Bu_4N)[2-B_{10}H_9NHC(Ph_3PCCN)CH_3)]$ **(4a)**

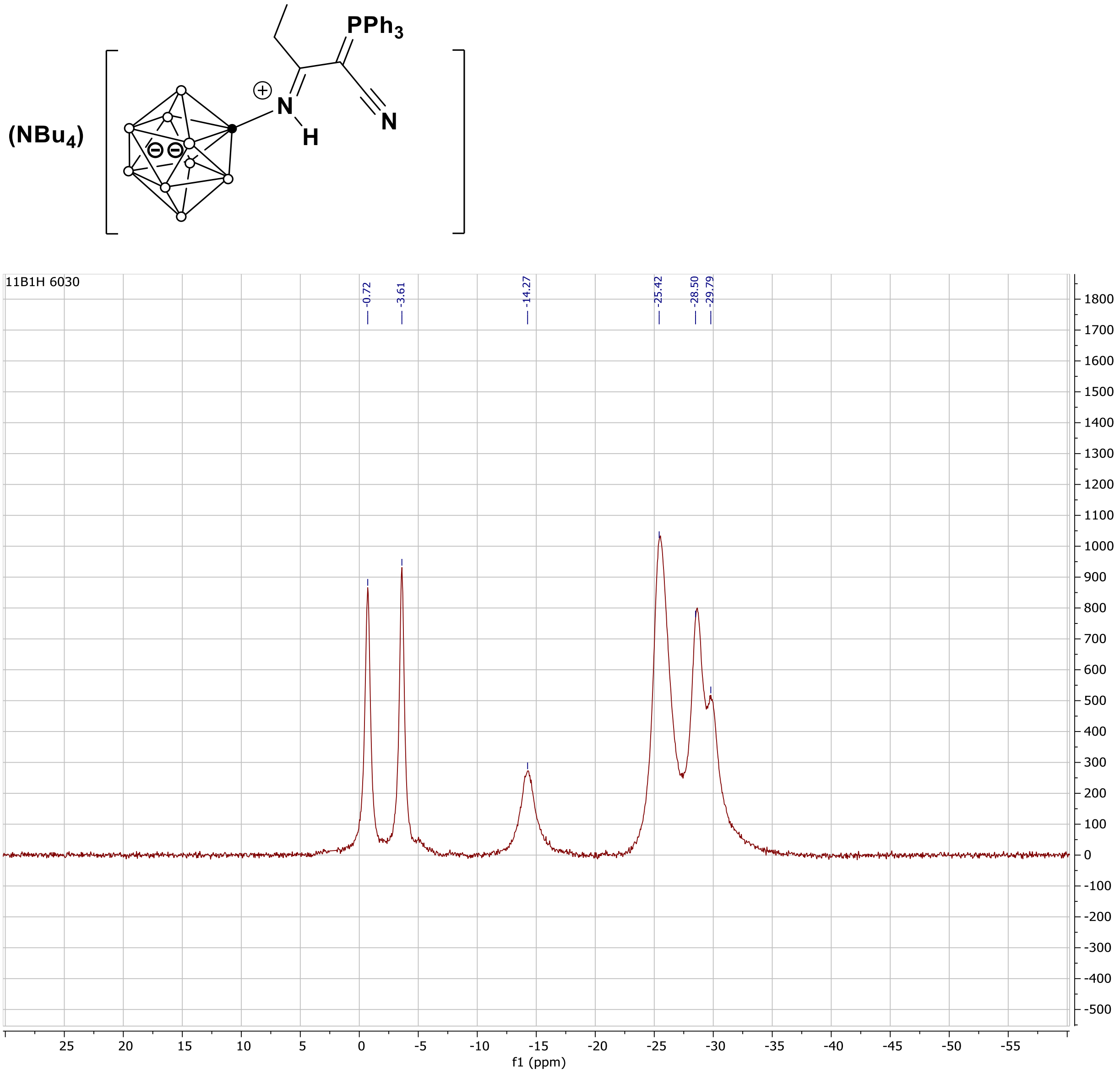

Figure S31. $^{11}B\{^{1}H\}$ **NMR spectrum of** $(Bu_4N)[2\text{-}B_{10}H_9NHC(Ph_3PCCN)C_2H_5)]$ **(4b)**

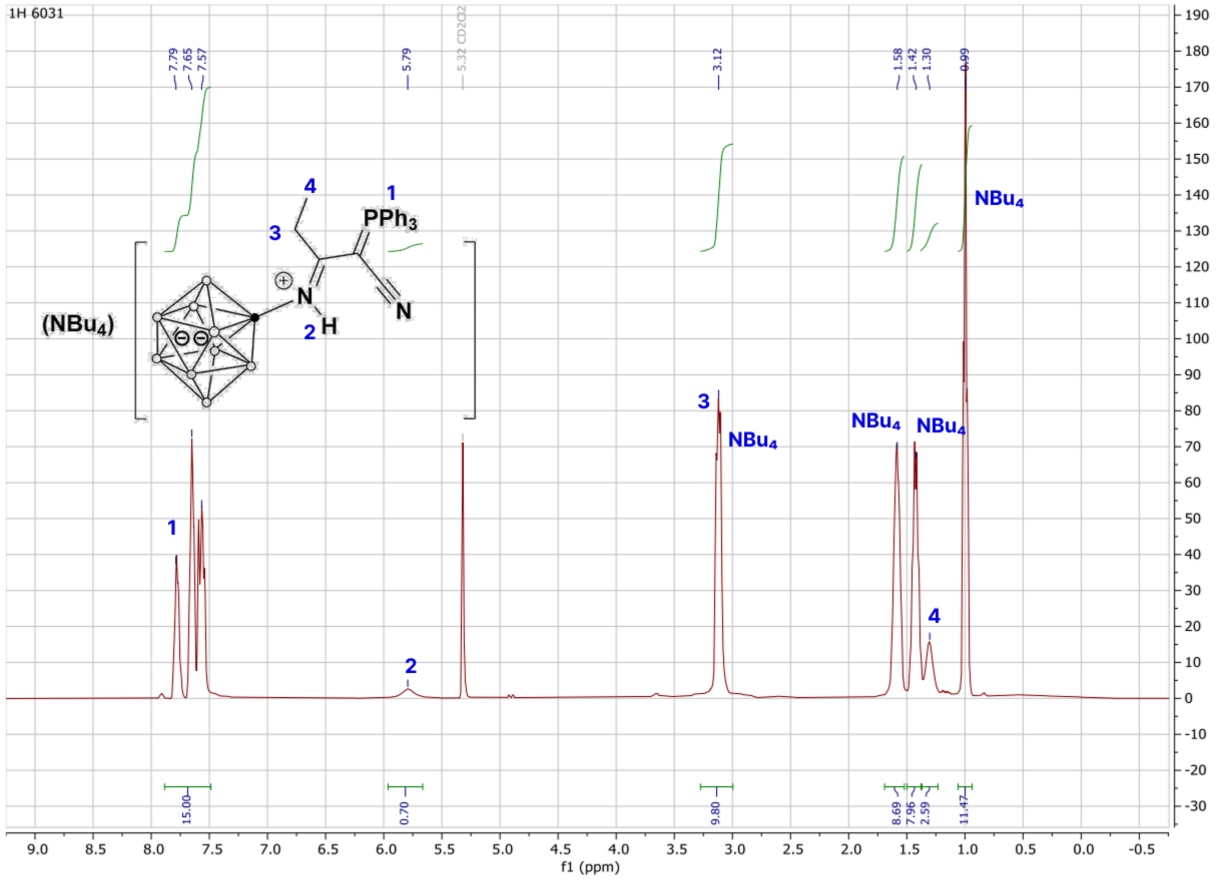

Figure S32. ^{1}H **NMR spectrum of** $(Bu_4N)[2\text{-}B_{10}H_9NHC(Ph_3PCCN)C_2H_5)]$ **(4b)**

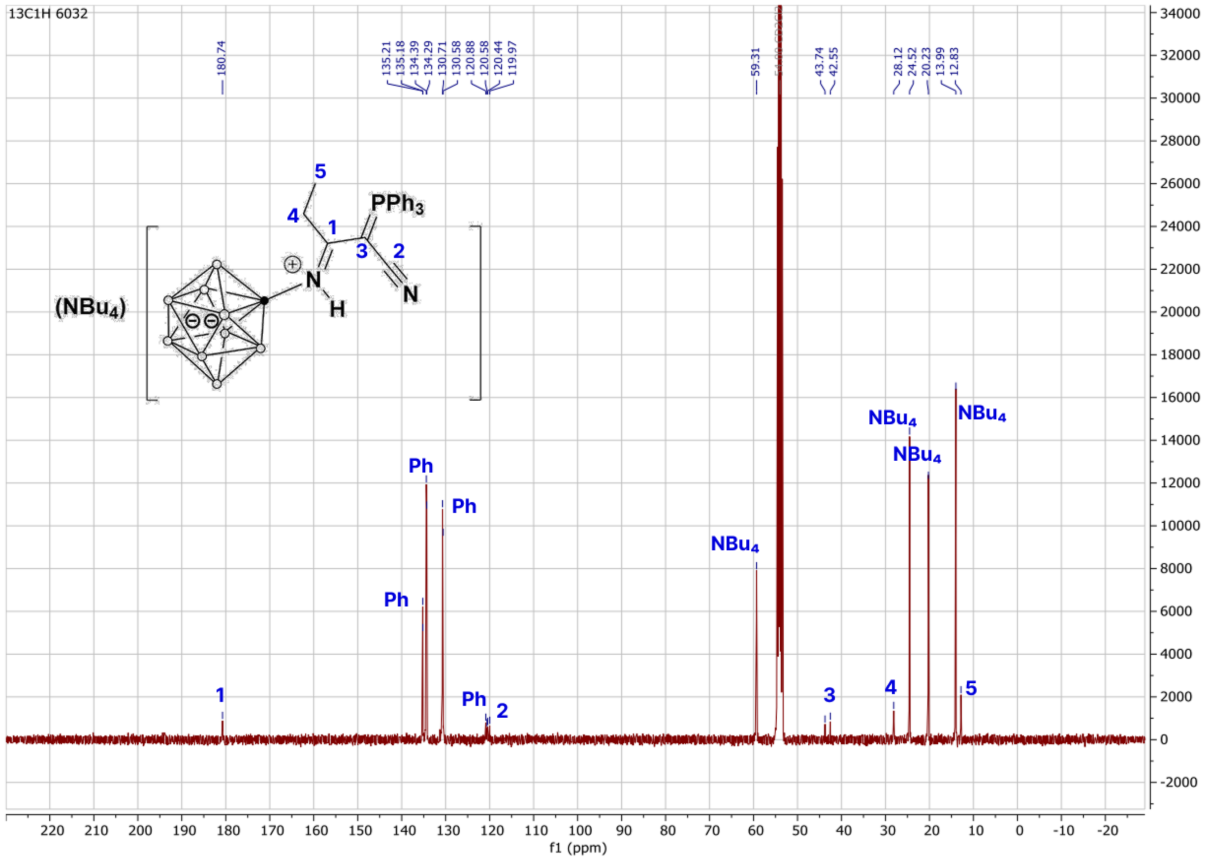

Figure S33. ^{13}C **NMR spectrum of** $(Bu_4N)[2\text{-}B_{10}H_9NHC(Ph_3PCCN)C_2H_5)]$ **(4b)**

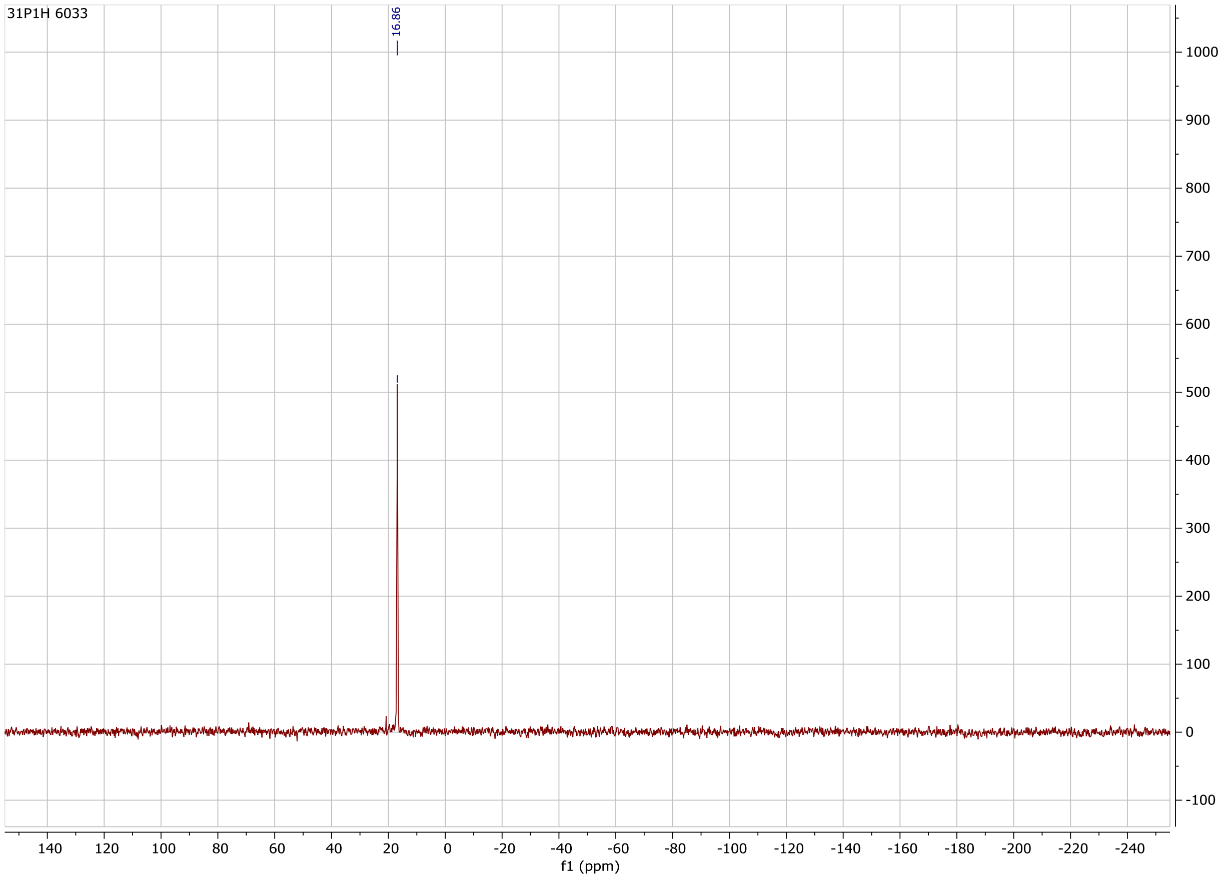

Figure S34. ^{31}**P NMR spectrum of** $(Bu_4N)[2\text{-}B_{10}H_9NHC(Ph_3PCCN)C_2H_5)]$ **(4b)**

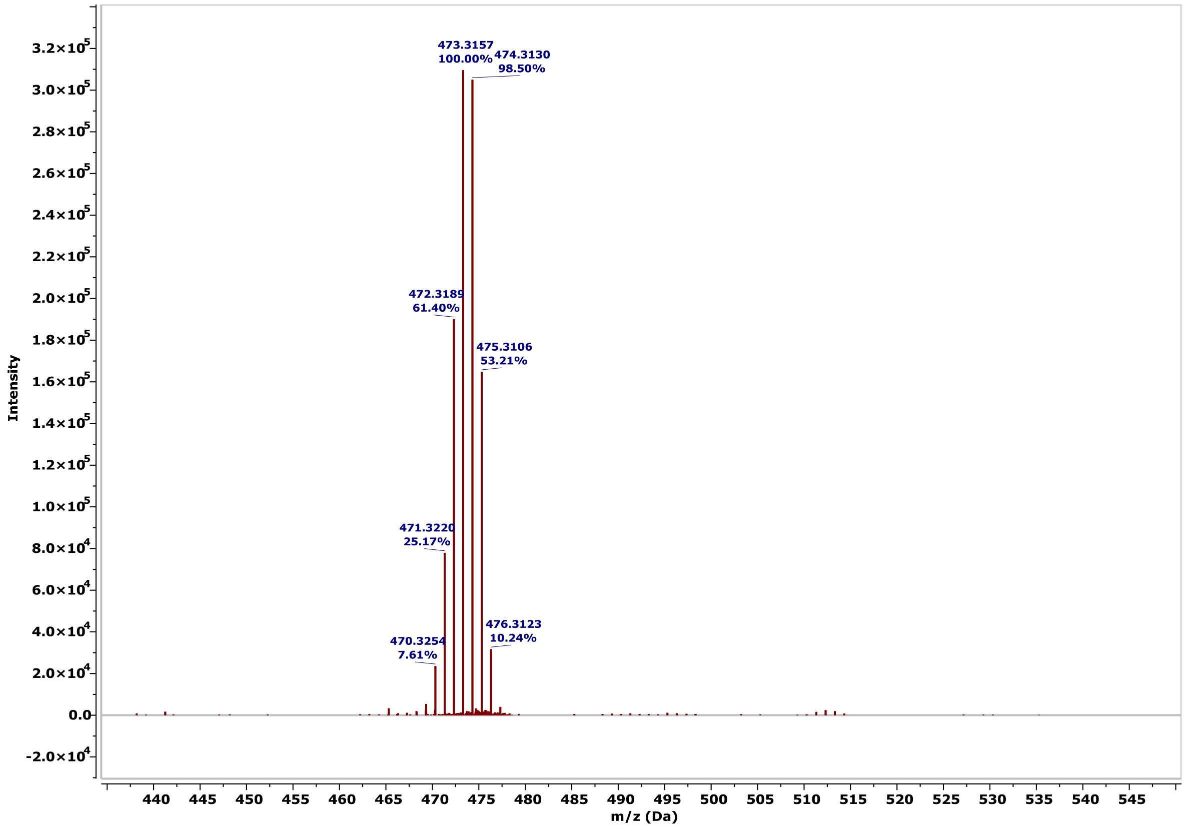

Figure S35. ESI-HRMS (negative area) spectrum of $(Bu_4N)[2\text{-}B_{10}H_9NHC(Ph_3PCCN)C_2H_5)]$ **(4b)**

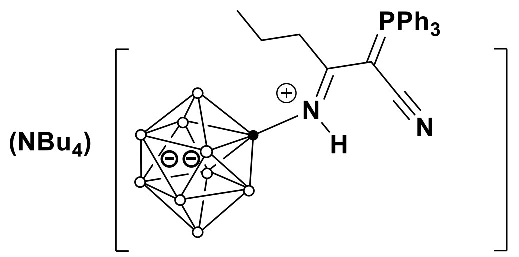

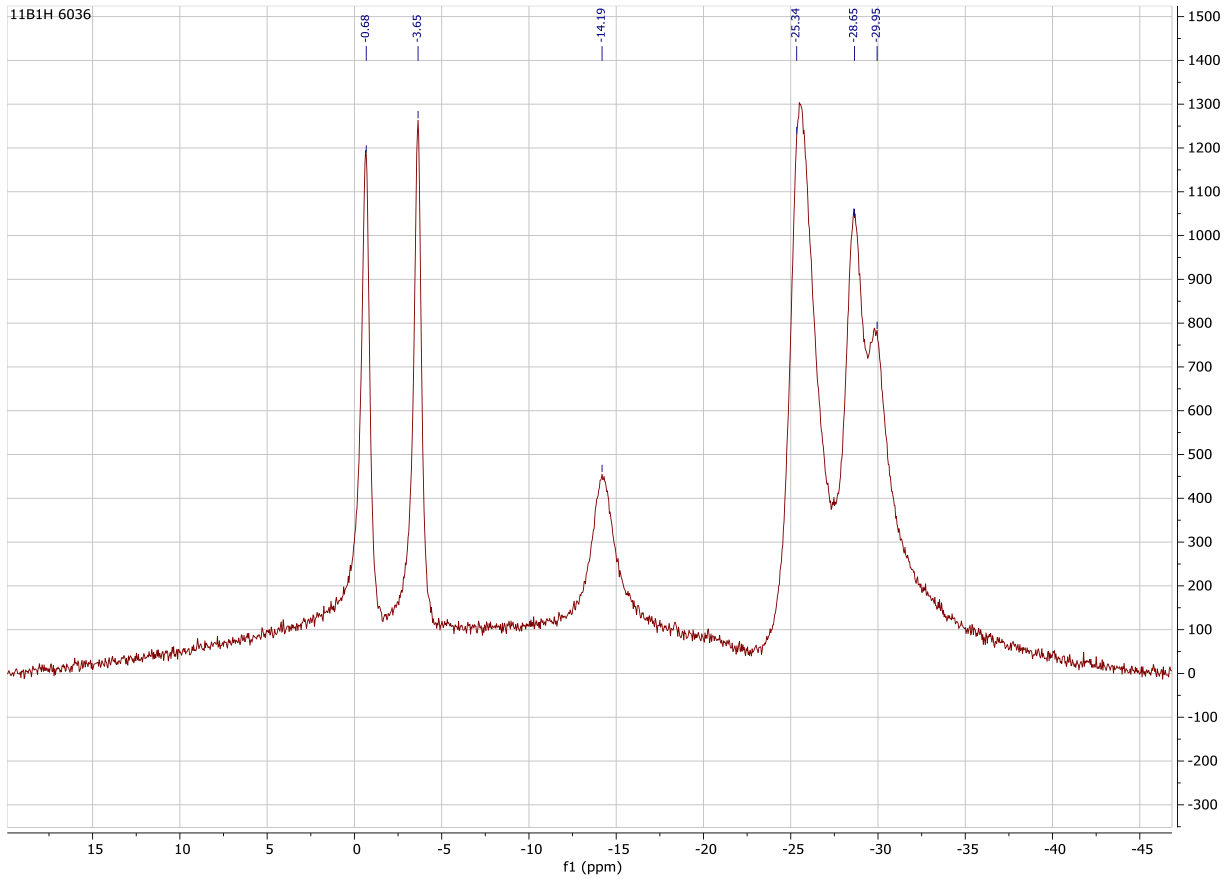

Figure S36. $^{11}B\{^{1}H\}$ **NMR spectrum of** $(Bu_4N)[2\text{-}B_{10}H_9NHC(Ph_3PCCN)^nC_3H_7)]$ **(4c)**

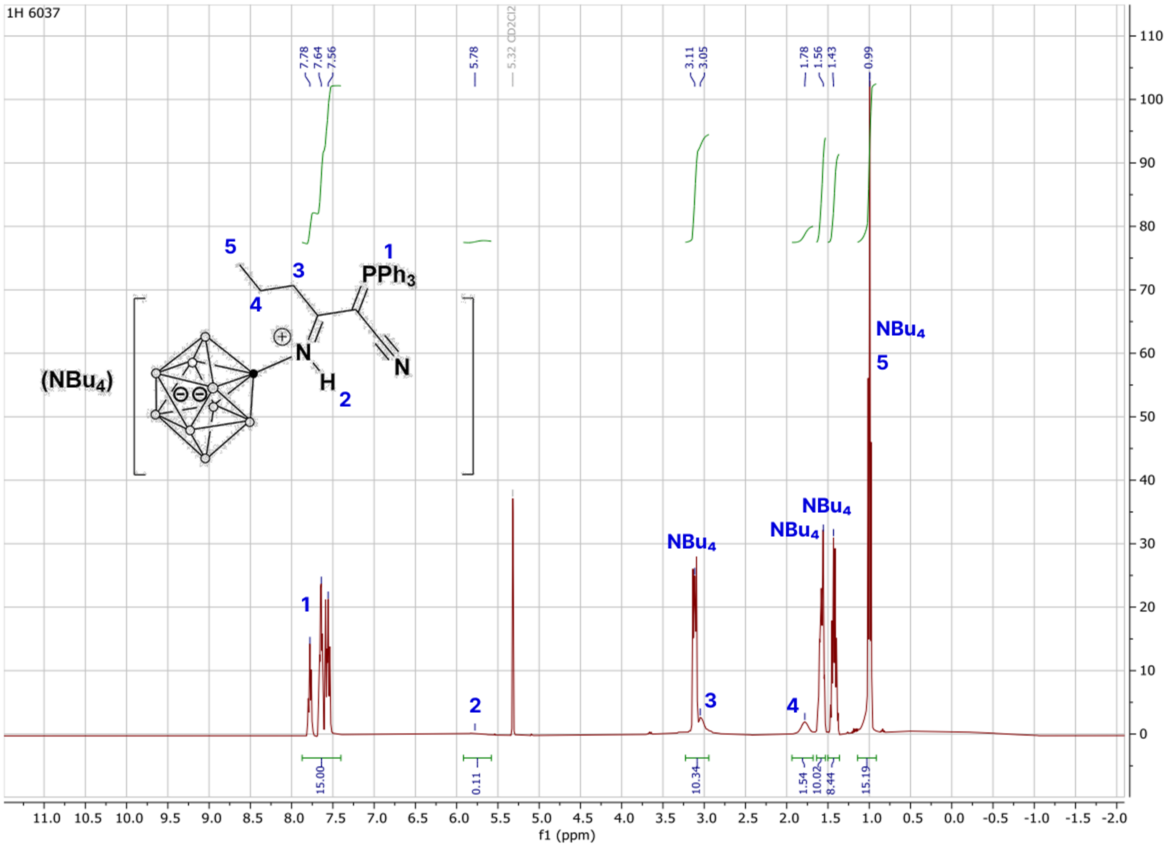

Figure S37. ^{1}H NMR spectrum of $(Bu_4N)[2\text{-}B_{10}H_9NHC(Ph_3PCCN)^nC_3H_7)]$ (4c)

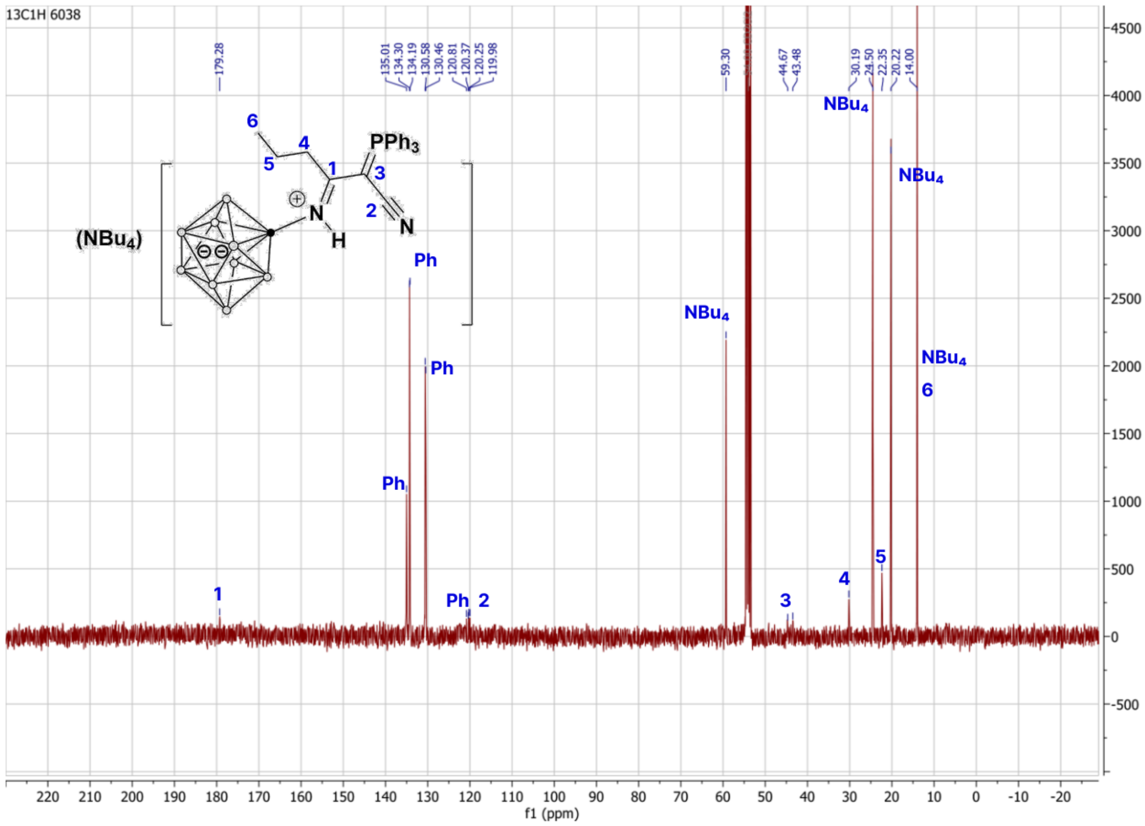

Figure S38. ^{13}C NMR spectrum of $(Bu_4N)[2\text{-}B_{10}H_9NHC(Ph_3PCCN)^nC_3H_7)]$ (4c)

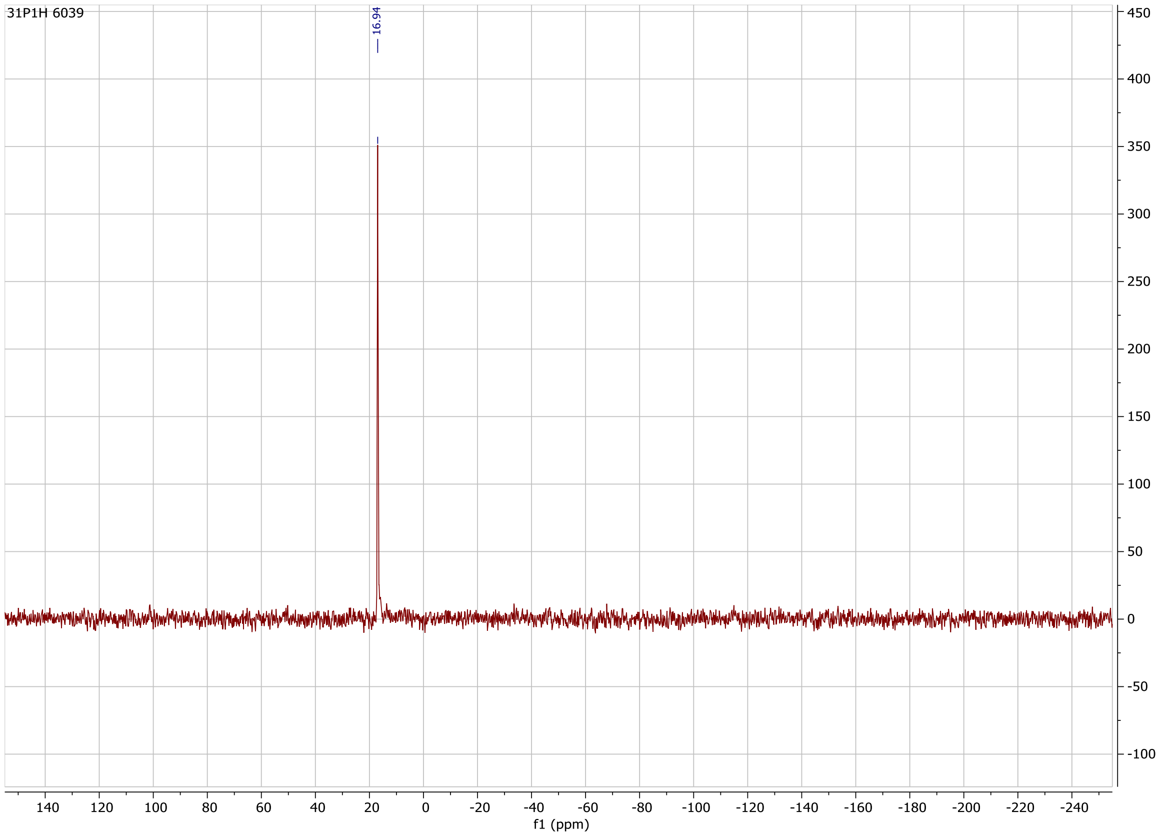

Figure S39. ^{31}P **NMR spectrum of** $(Bu_4N)[2\text{-}B_{10}H_9NHC(Ph_3PCCN)^nC_3H_7)]$ **(4c)**

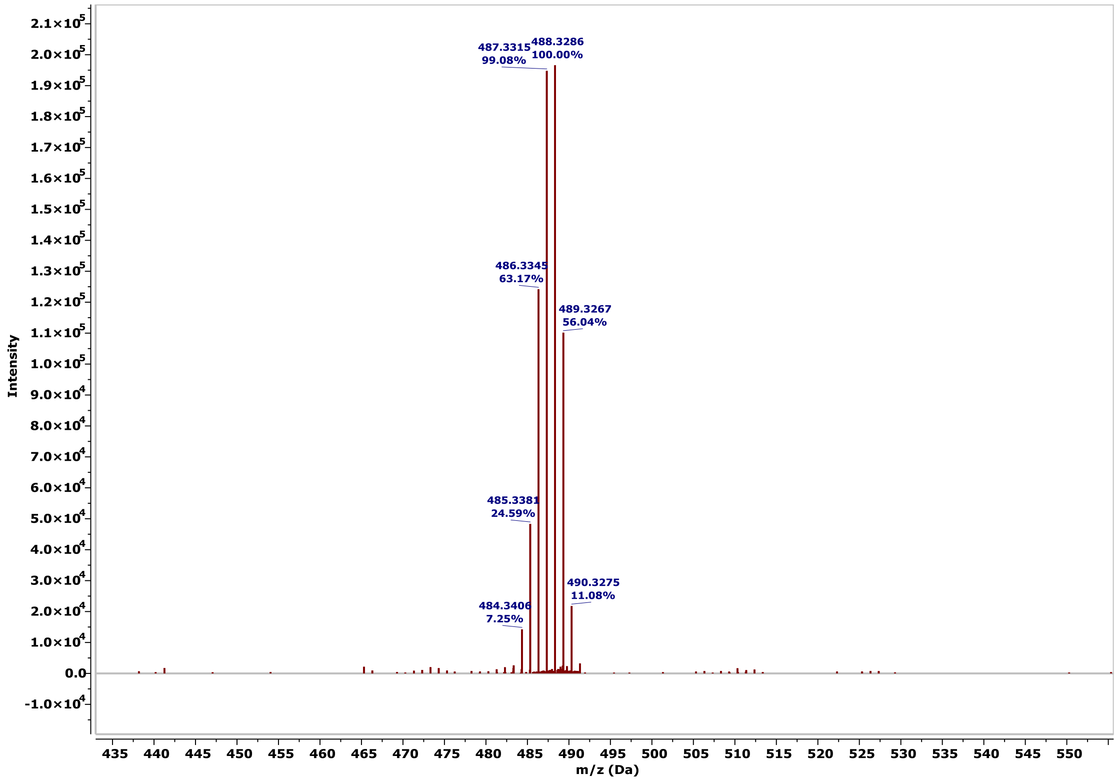

Figure S40. ESI-HRMS (negative area) spectrum of $(Bu_4N)[2\text{-}B_{10}H_9NHC(Ph_3PCCN)^nC_3H_7)]$ **(4c)**

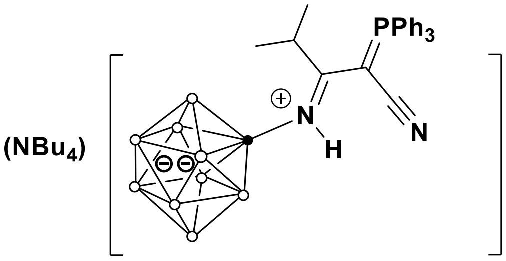

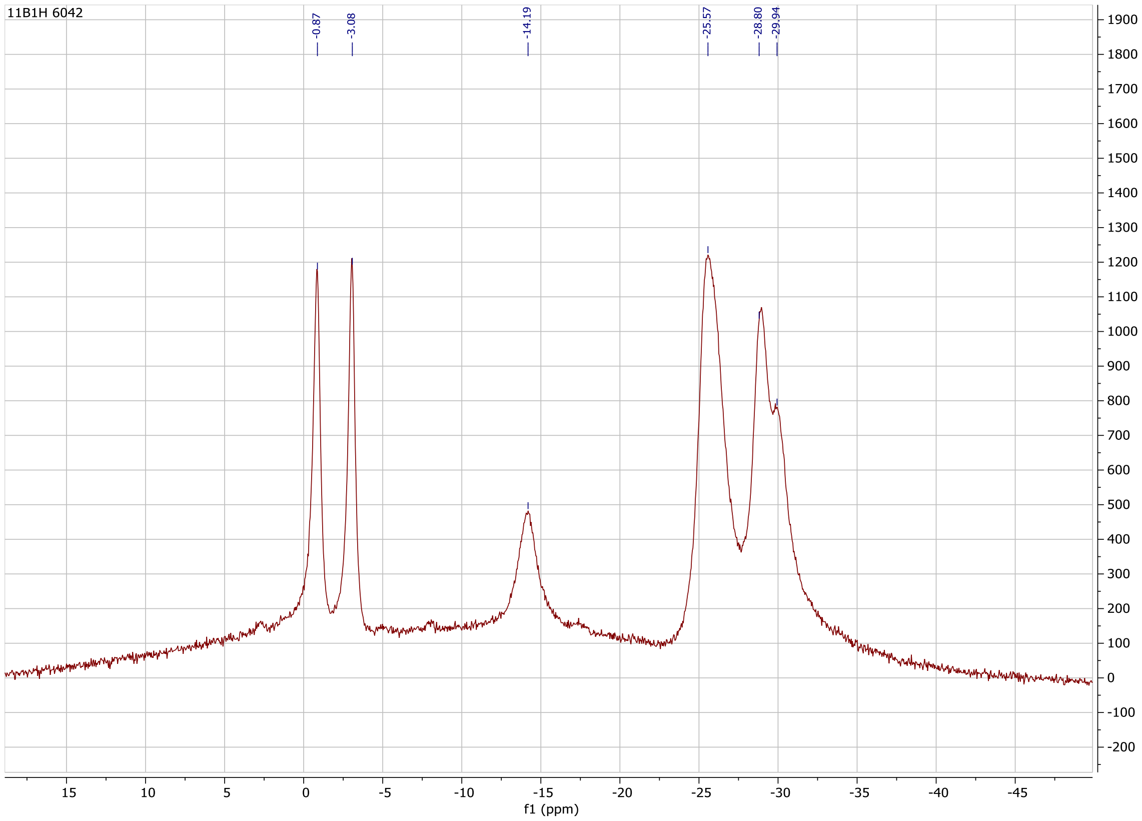

Figure S41. $^{11}B\{^1H\}$ NMR spectrum of $(Bu_4N)[2\text{-}B_{10}H_9NHC(Ph_3PCCN)^iC_3H_7)]$ (4d).

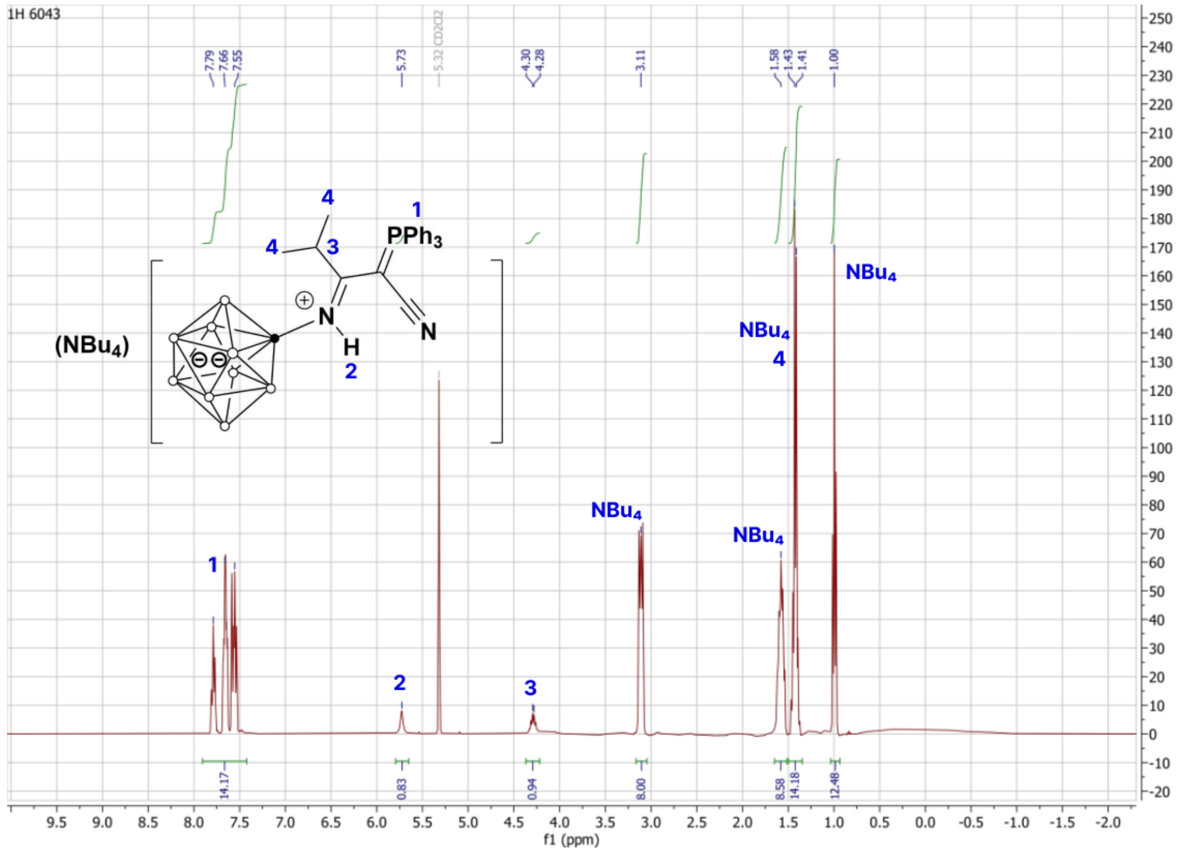

Figure S42. ^{1}**H NMR spectrum of (Bu$_4$N)[2-B$_{10}$H$_9$NHC(Ph$_3$PCCN)^{i}C$_3$H$_7$)]** (**4d**).

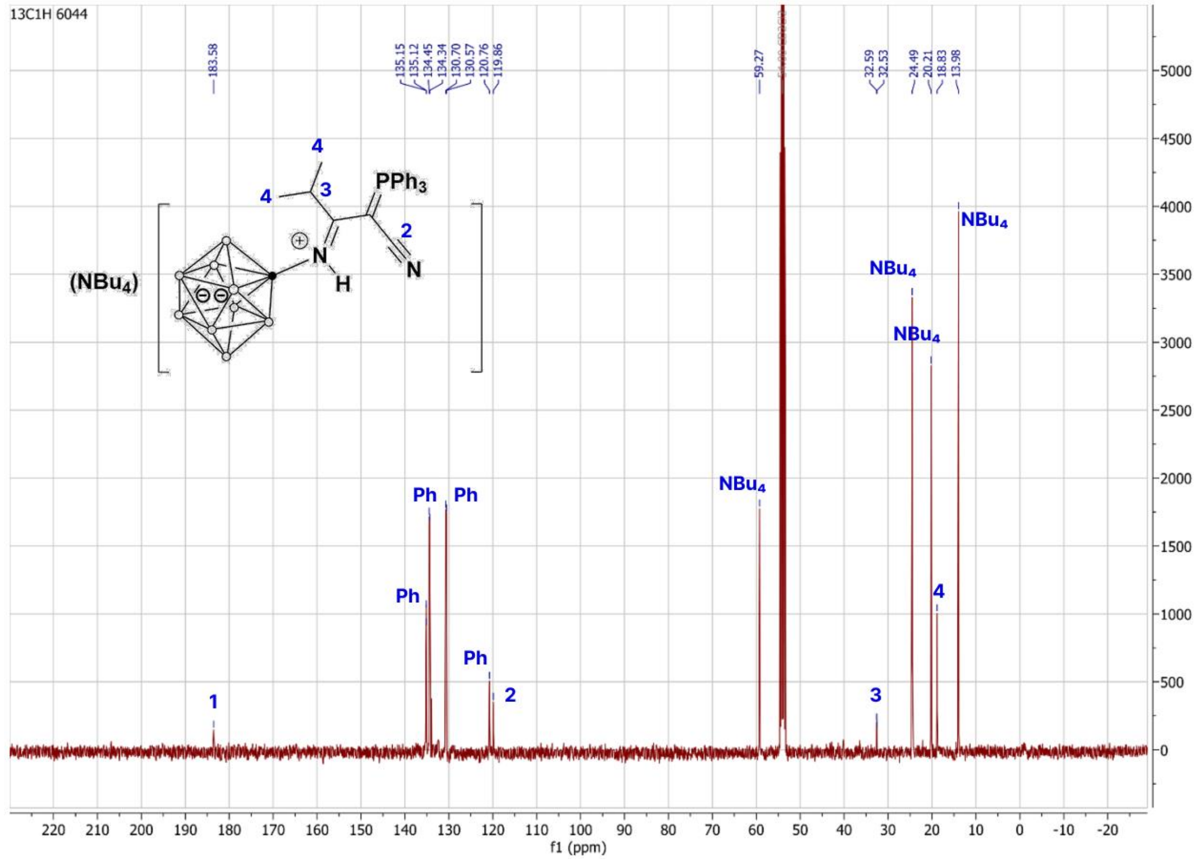

Figure S43. ^{13}**C NMR spectrum of (Bu$_4$N)[2-B$_{10}$H$_9$NHC(Ph$_3$PCCN)^{i}C$_3$H$_7$)]** (**4d**).

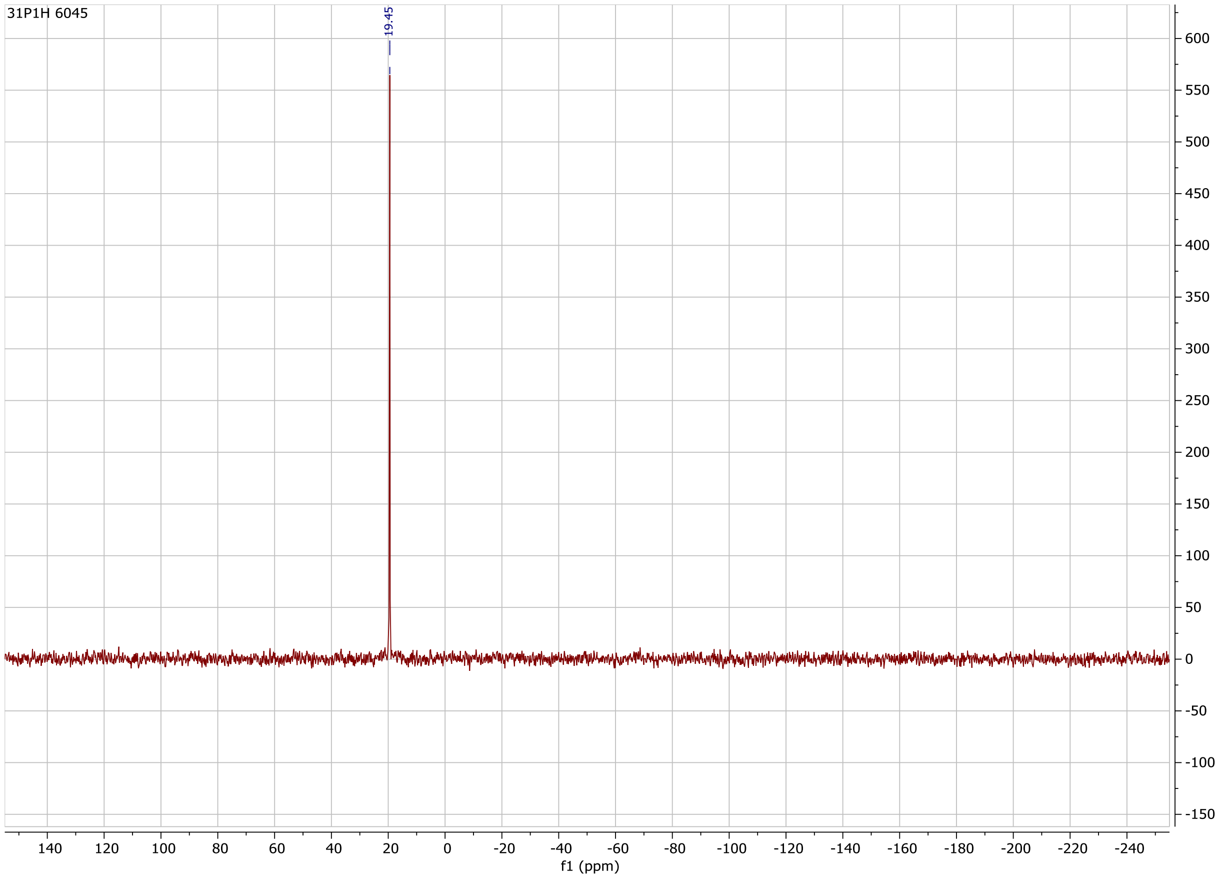

Figure S44. ^{31}**P NMR spectrum of** $(Bu_4N)[2\text{-}B_{10}H_9NHC(Ph_3PCCN)^iC_3H_7)]$ (**4d**).

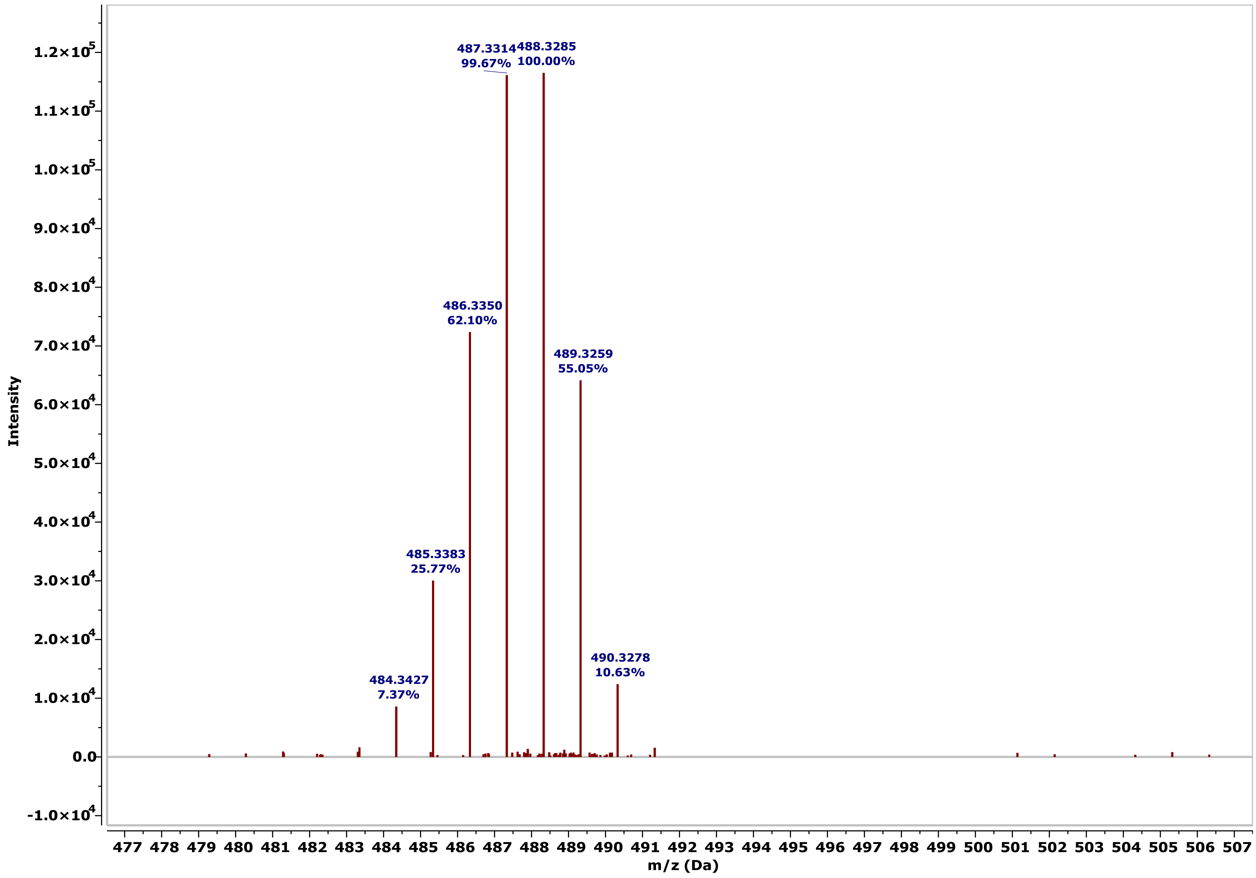

Figure S45. ESI-HRMS (negative area) spectrum of $(Bu_4N)[2\text{-}B_{10}H_9NHC(Ph_3PCCN)^iC_3H_7)]$ (4d)

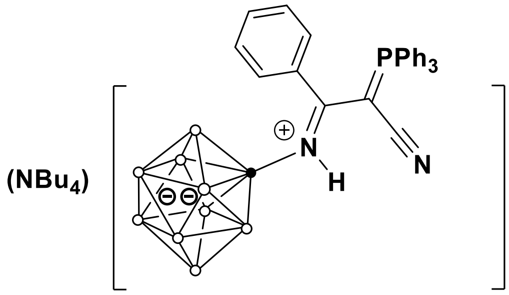

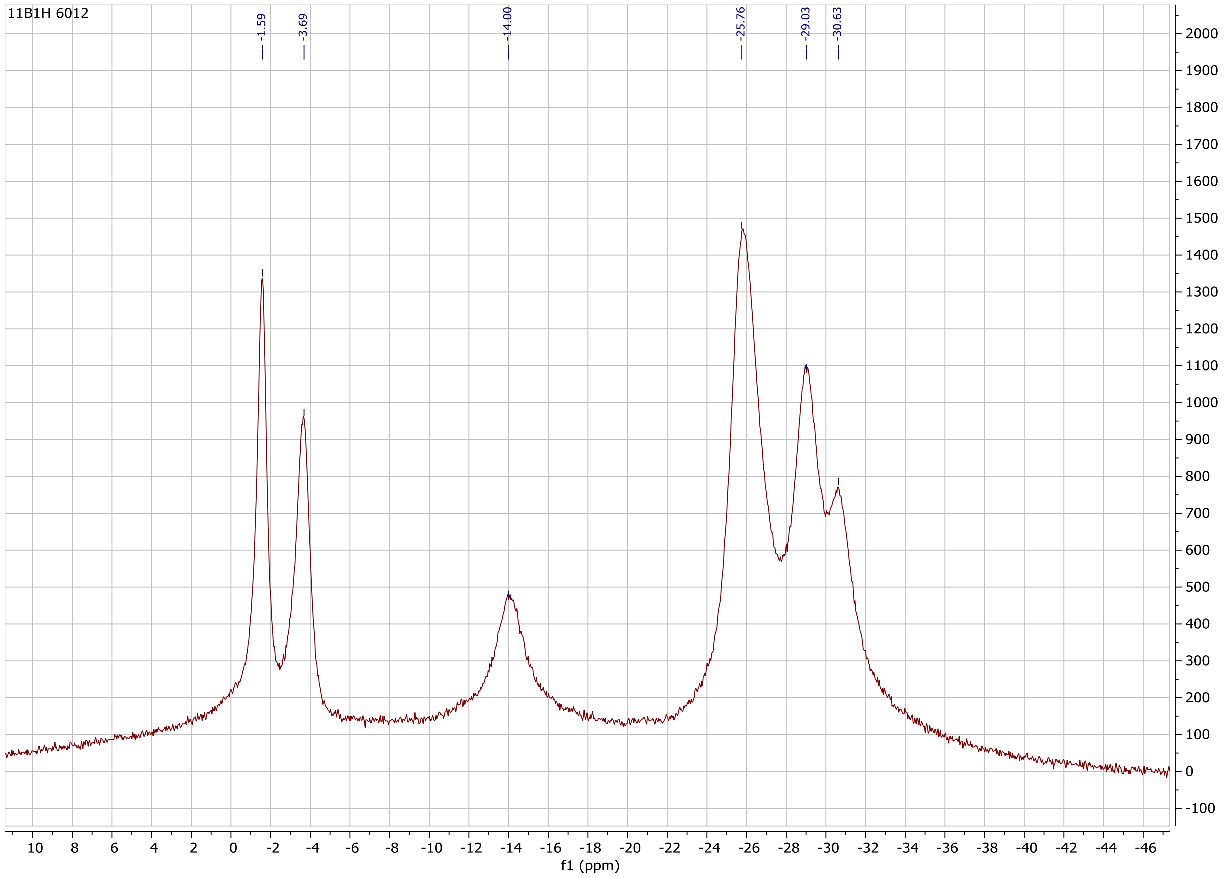

Figure S46. $^{11}B\{^1H\}$ NMR spectrum of $(Bu_4N)[2\text{-}B_{10}H_9NHC(Ph_3PCCN)C_6H_5)]$ (4e).

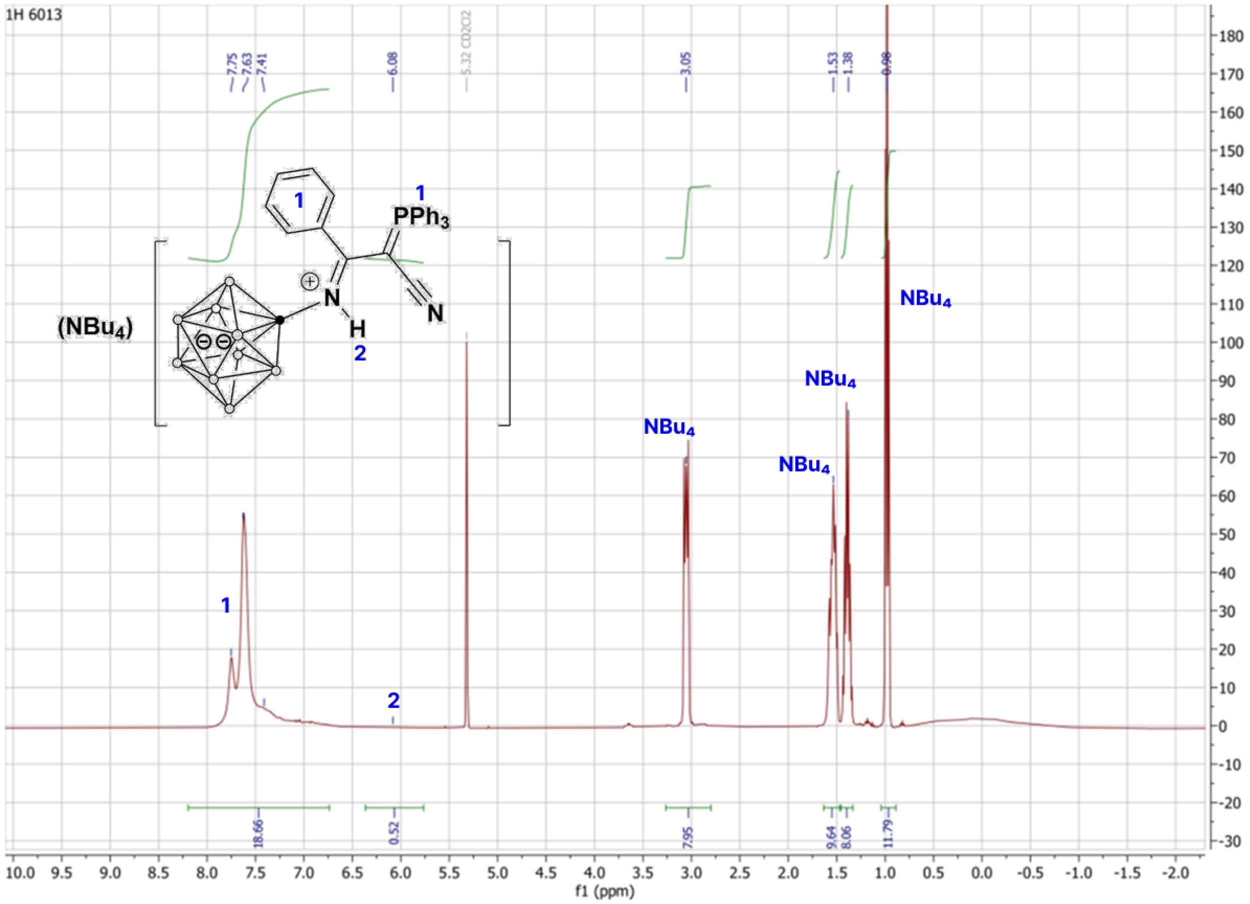

Figure S47. **^{1}H NMR spectrum of (Bu_4N)[2-$B_{10}H_9$NHC(Ph_3PCCN)C_6H_5)] (4e)**.

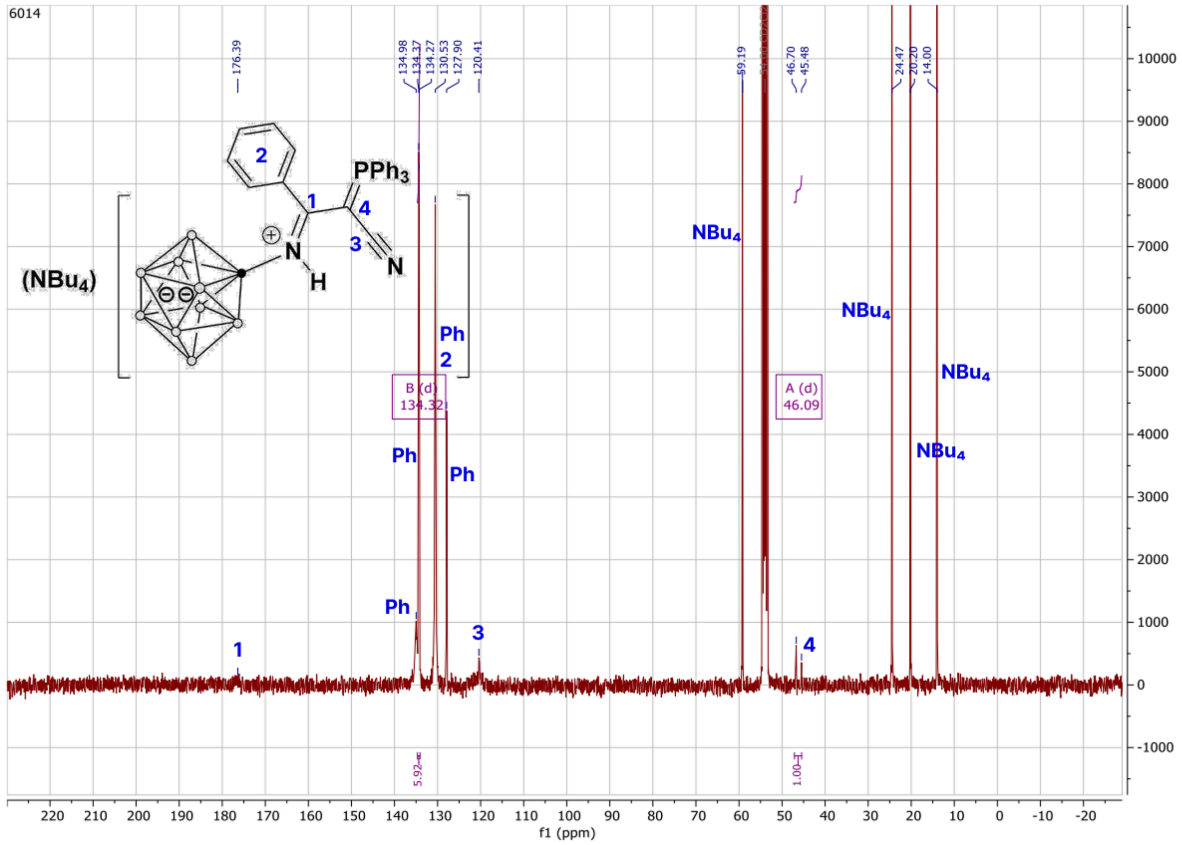

Figure S48. **^{13}C NMR spectrum of (Bu_4N)[2-$B_{10}H_9$NHC(Ph_3PCCN)C_6H_5)] (4e)**.

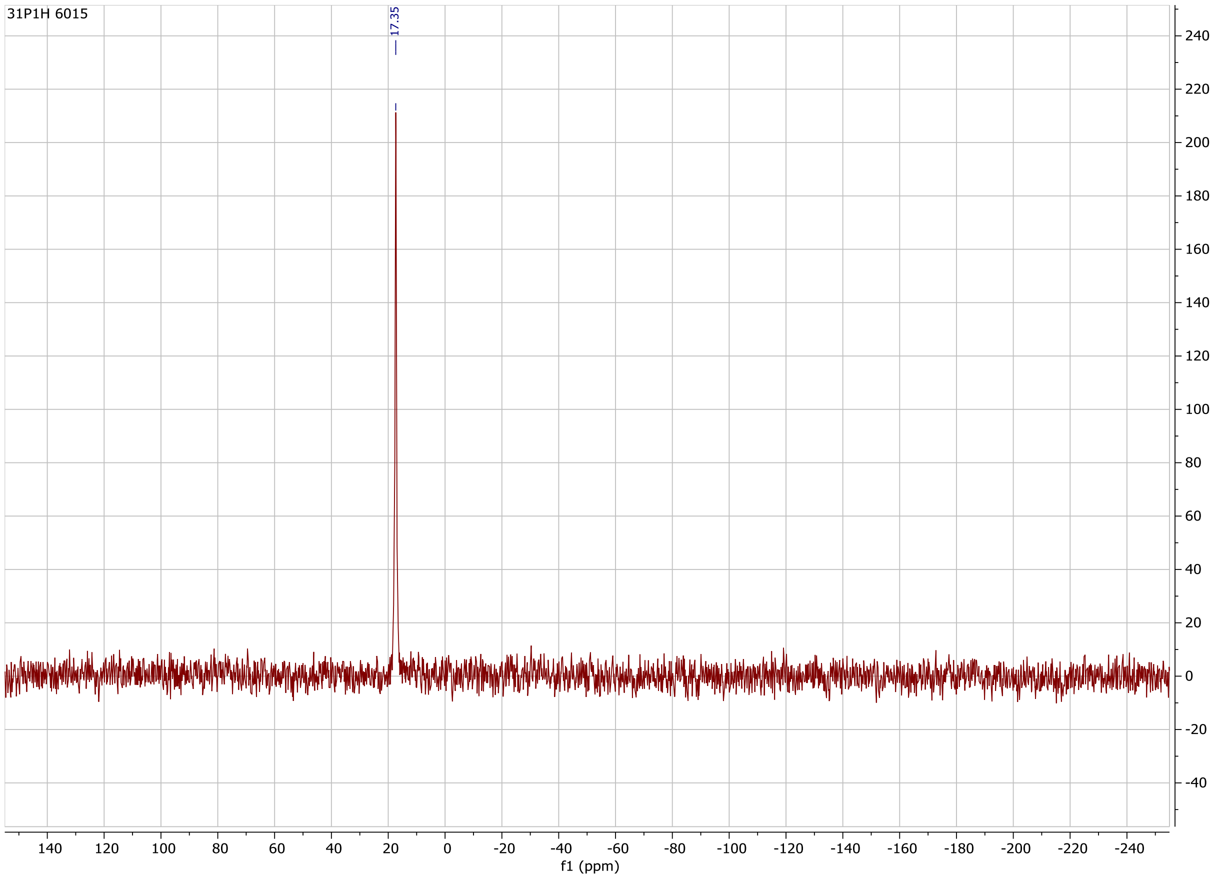

Figure S49. ^{31}**P NMR spectrum of** $(Bu_4N)[2\text{-}B_{10}H_9NHC(Ph_3PCCN)C_6H_5)]$ **(4e).**

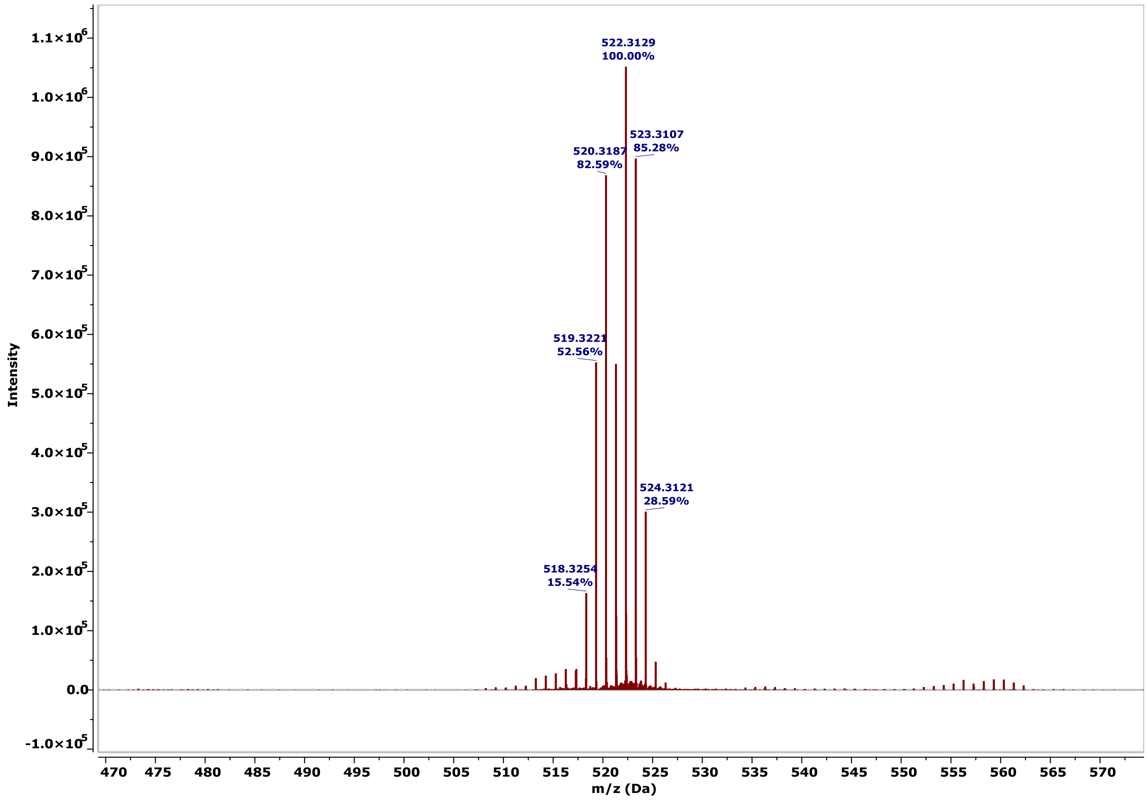

Figure S50. ESI-HRMS (negative area) spectrum of $(Bu_4N)[2\text{-}B_{10}H_9NHC(Ph_3PCCN)C_6H_5)]$ **(4e)**

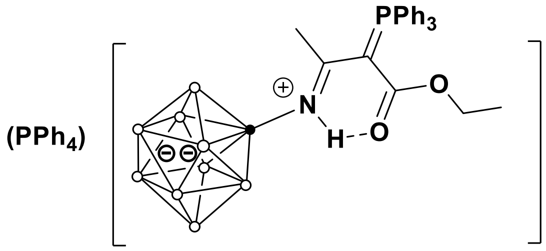

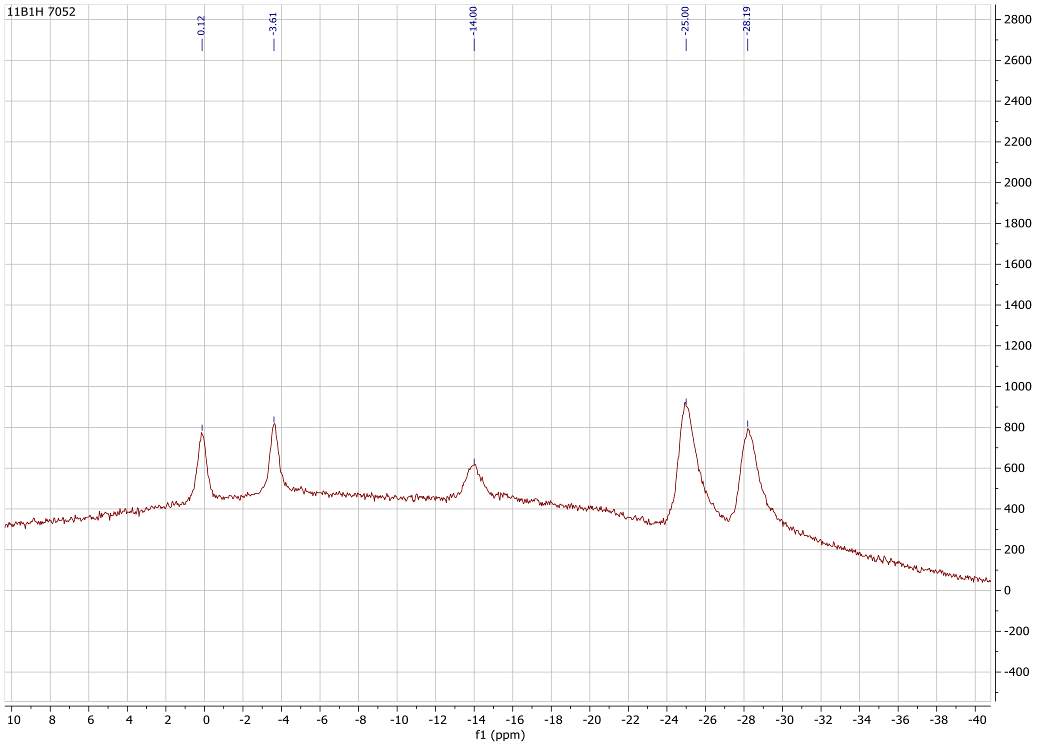

Figure S51. $^{11}B\{^{1}H\}$ NMR spectrum of $(Ph_4P)[2\text{-}B_{10}H_9NHC(Ph_3PCCOOEt)CH_3)]$ Ph_4P(3a).

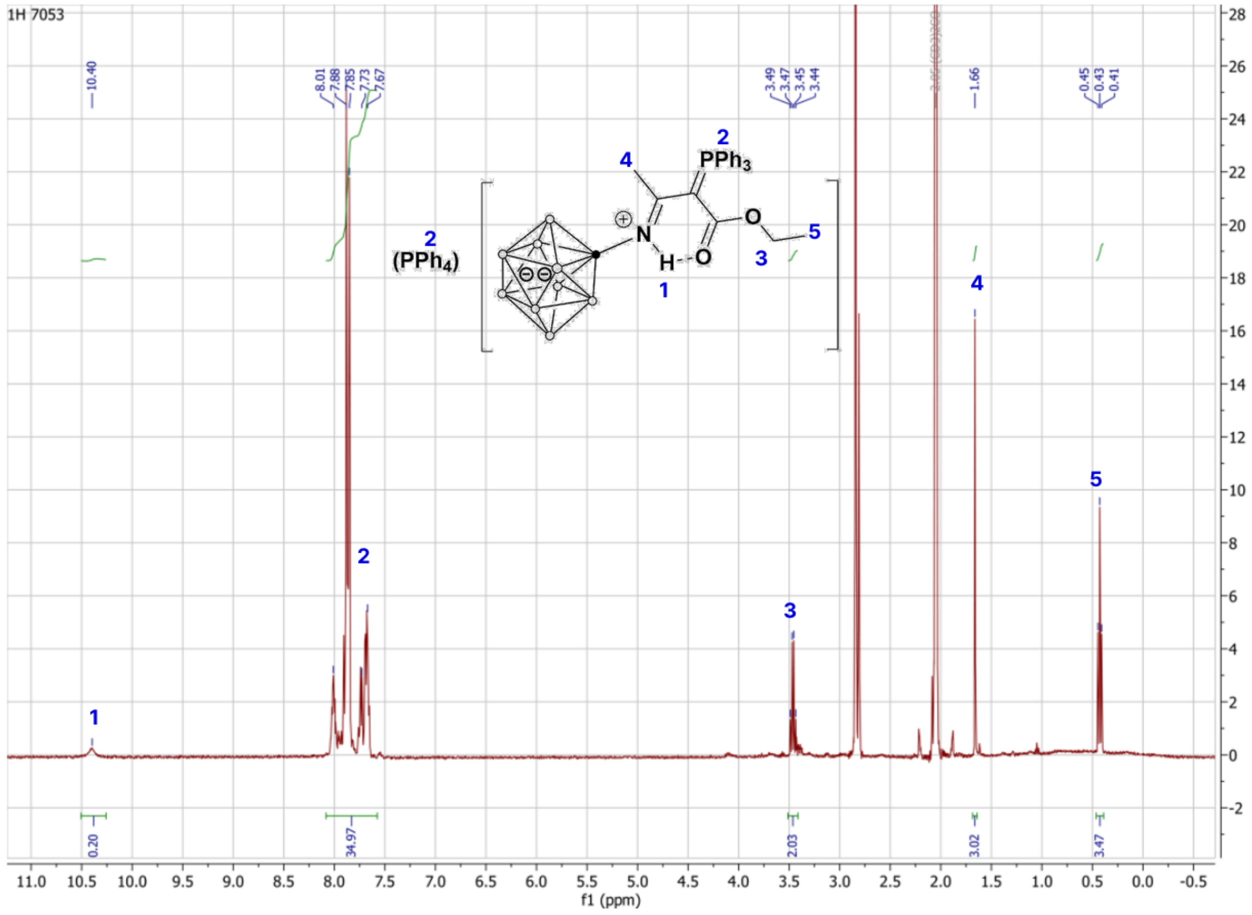

Figure S52. **^{1}H NMR spectrum of $(Ph_4P)[2\text{-}B_{10}H_9NHC(Ph_3PCCOOEt)CH_3)]$ Ph_4P(3a).**

Table 1S. Crystal data and structure refinement for Ph_4P3a and 4b.

Identification code	Ph_4P3a	4b
**Empirical formula**	$C_{48}H_{53}B_{10}NO_2P_2$	$C_{39}H_{66}B_{10}N3P$
**Formula weight**	845.95	716.01
**Temperature/K**	100	100
**Crystal system**	triclinic	monoclinic
**Space group**	P-1	$P2_1/n$
**a/Å**	10.6876 (5)	8.778 (3) Å
**b/Å**	13.1011 (7)	17.178 (4) Å
**c/Å**	18.1250 (9)	28.907 (8) Å
**α/°**	95.062 (2)	
**β/°**	101.130 (2)	95.345 (7)°
**γ/°**	110.664 (2)	
**Volume/Å^3**	2295.9 (2)	4340 (2) Å^3
**Z**	2	4
**ρ_{calc}g/cm^3**	1.224	1.096
**μ/mm^{-1}**	0.14	0.09
**F(000)**	888	1544
**Radiation**	MoKα (λ = 0.71073)	MoKα (λ = 0.71073)
**Reflections collected**	20485	25893
**Independent reflections**	10298 [R_{int} = 0.068]	7648 [R_{int} = 0.0279]
**Data/restraints/parameters**	10298/2/613	7648/0/479
**Goodness-of-fit on F^2**	0.950	1.025
**Final R indexes [I>=2σ (I)]**	R_1 = 0.063, wR_2 = 0.129	R_1 = 0.1328, wR_2 = 0.2909
**Final R indexes [all data]**	R_1 = 0.1495, wR_2 = 0.2738	R_1 = 0.2913, wR_2 = 0.3706

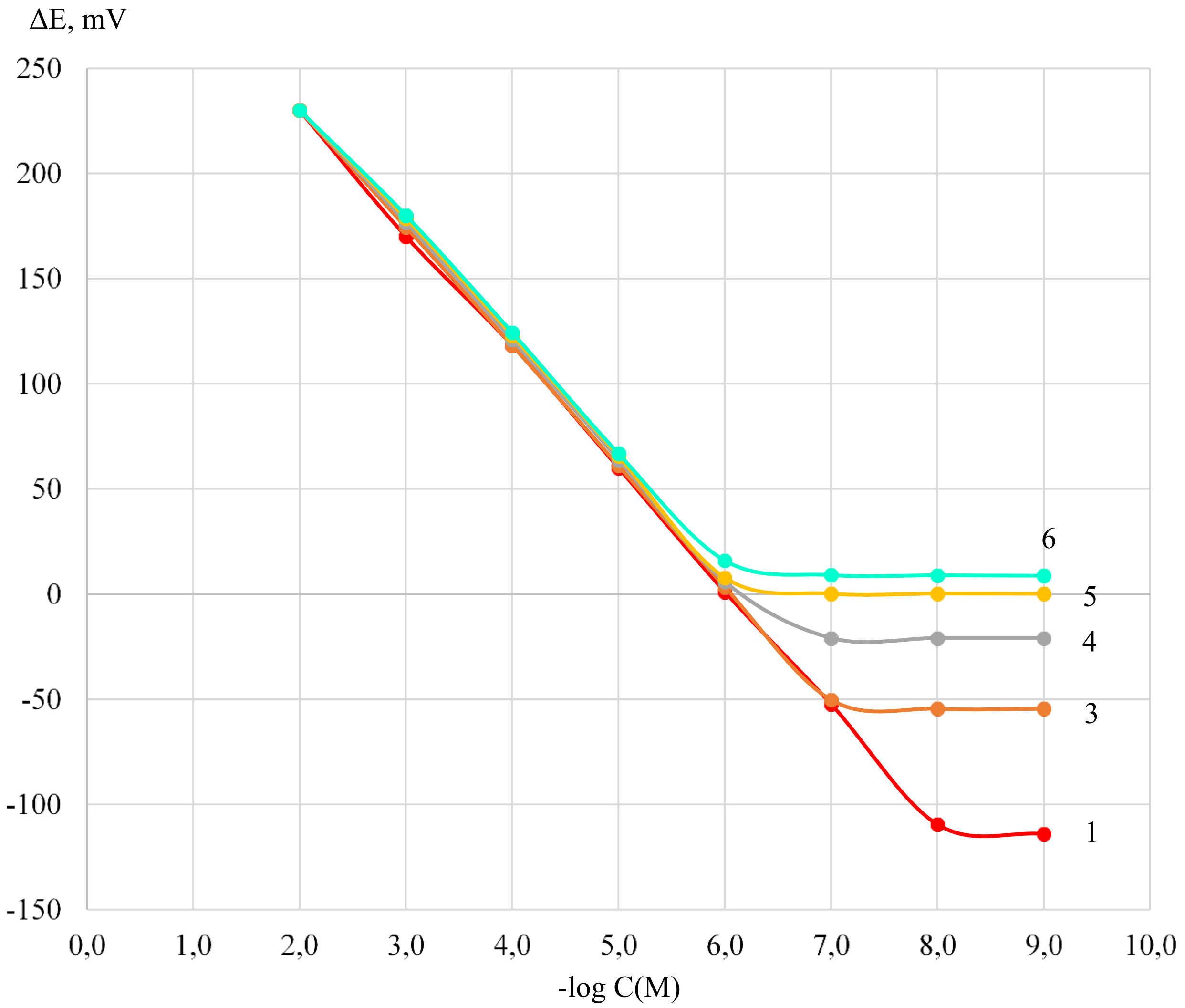

Figure S53. Averaged calibration curves for membranes 1, 3, 4, 5, 6.

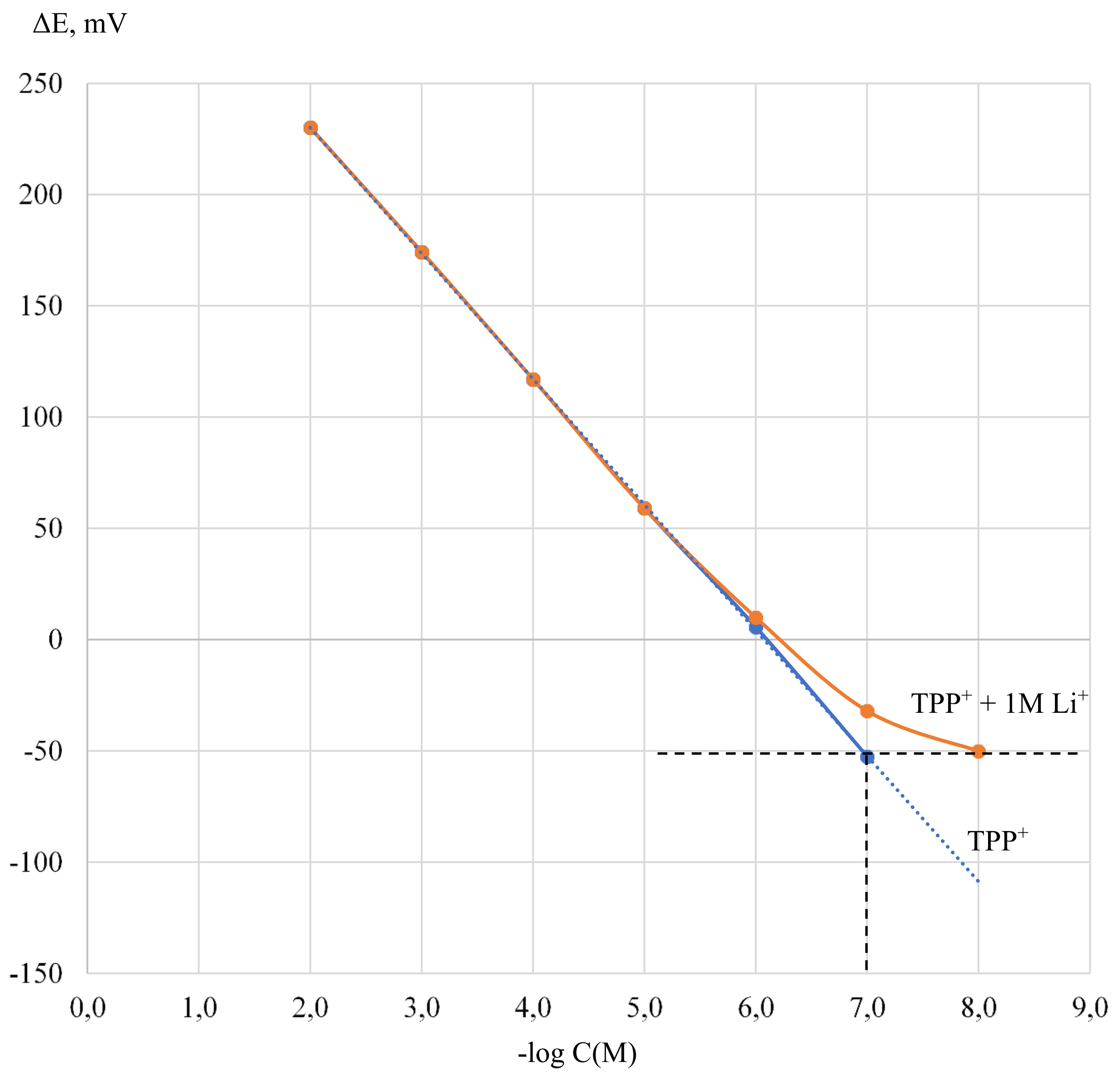

Figure S54. Graphical determination of the selectivity coefficient $pK^{pot}_{TPP^+/Li^+}$

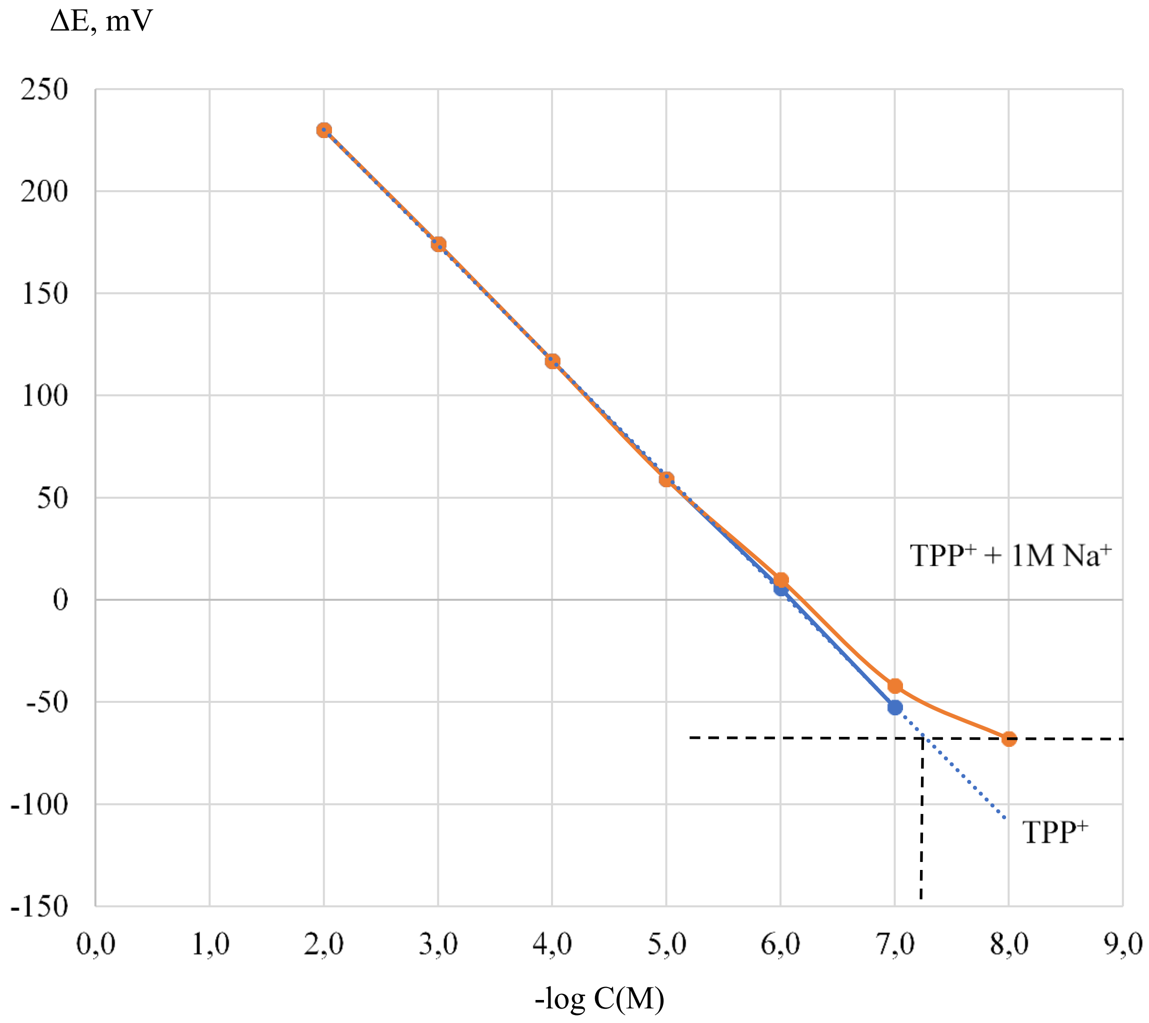

Figure S55. Graphical determination of the selectivity coefficient $pK^{pot}_{TPP^{+}/Na^{+}}$

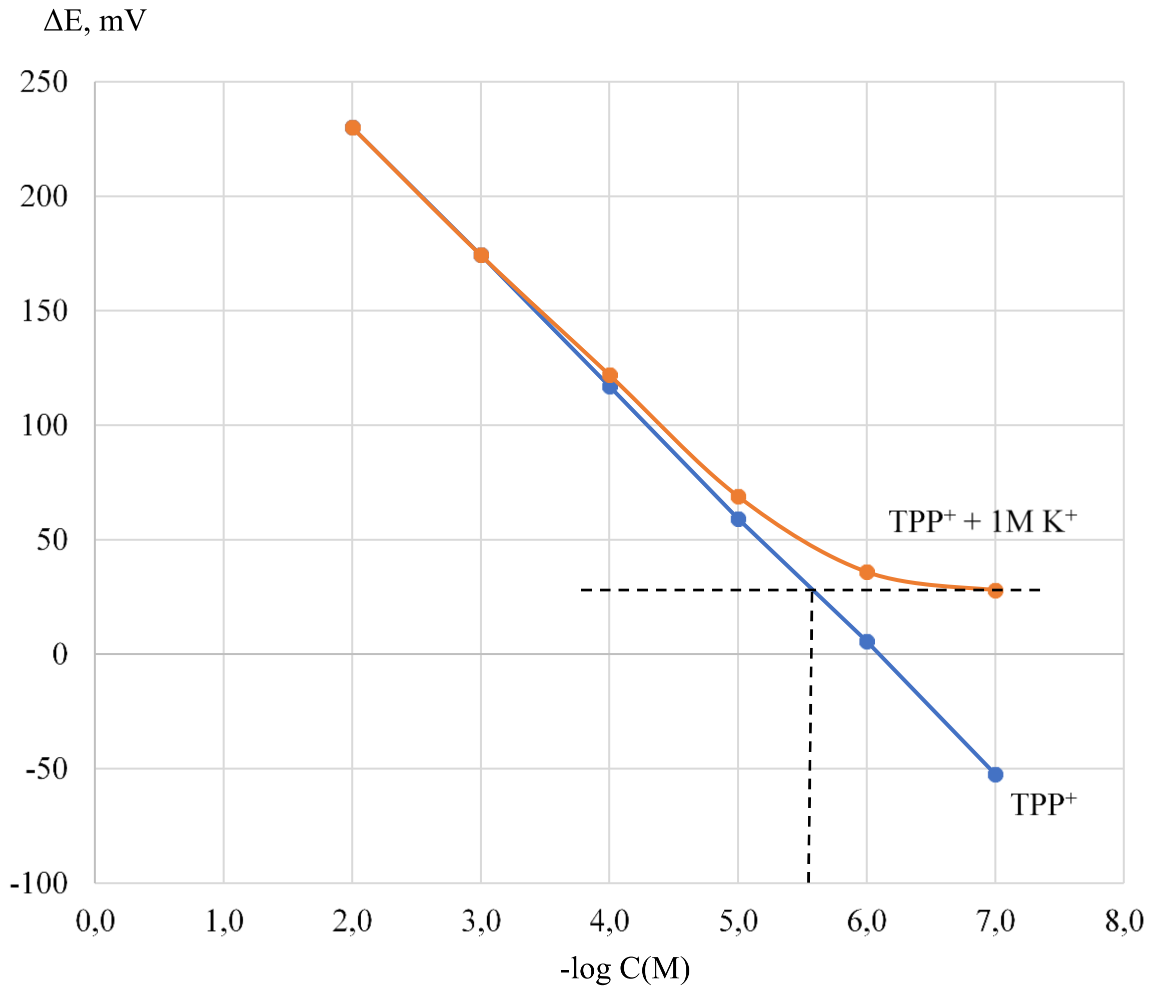

Figure S56. Graphical determination of the selectivity coefficient $pK^{pot}_{TPP^+/K^+}$

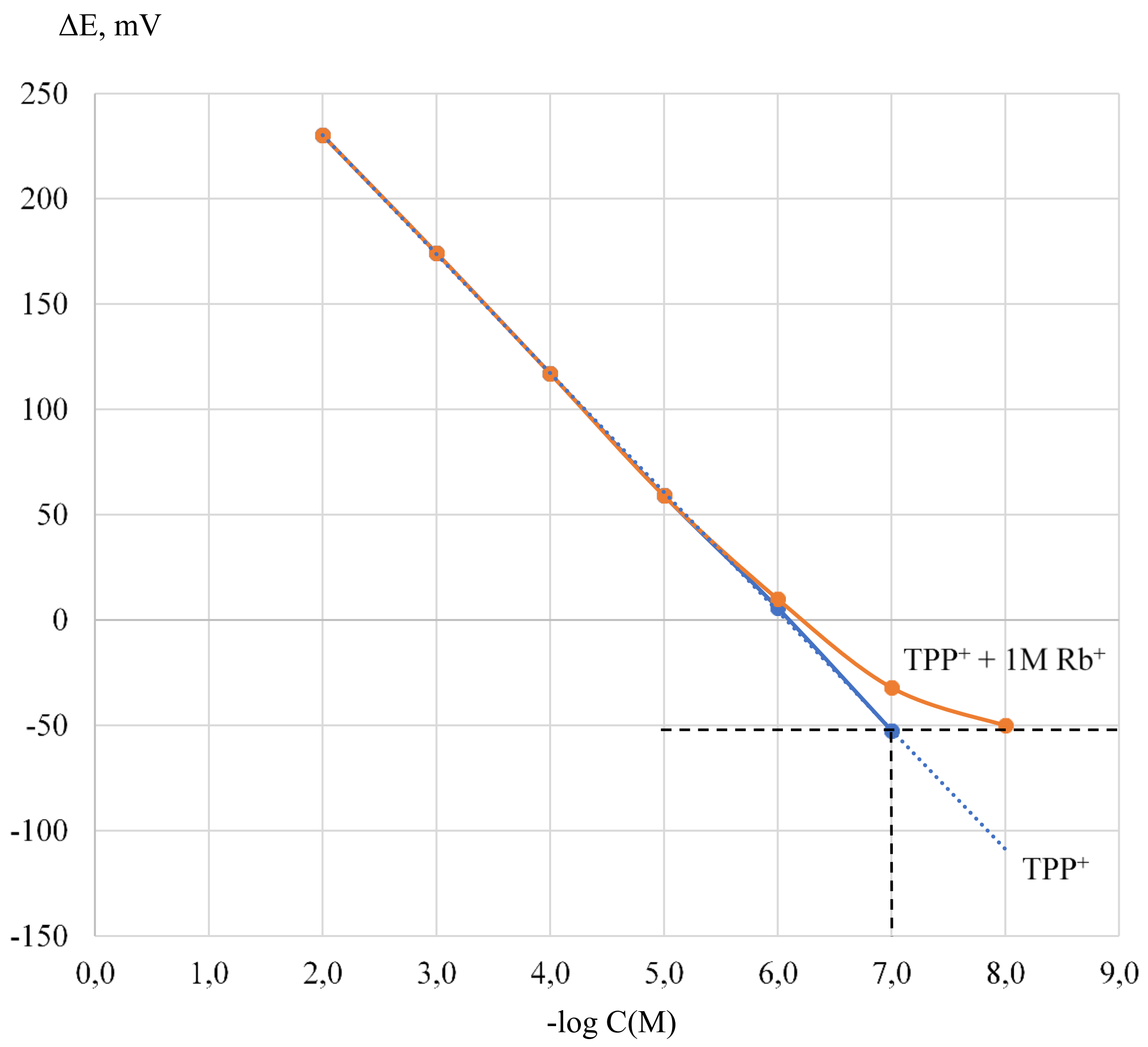

Figure S57. Graphical determination of the selectivity coefficient $pK^{pot}_{TPP^+/Rb^+}$

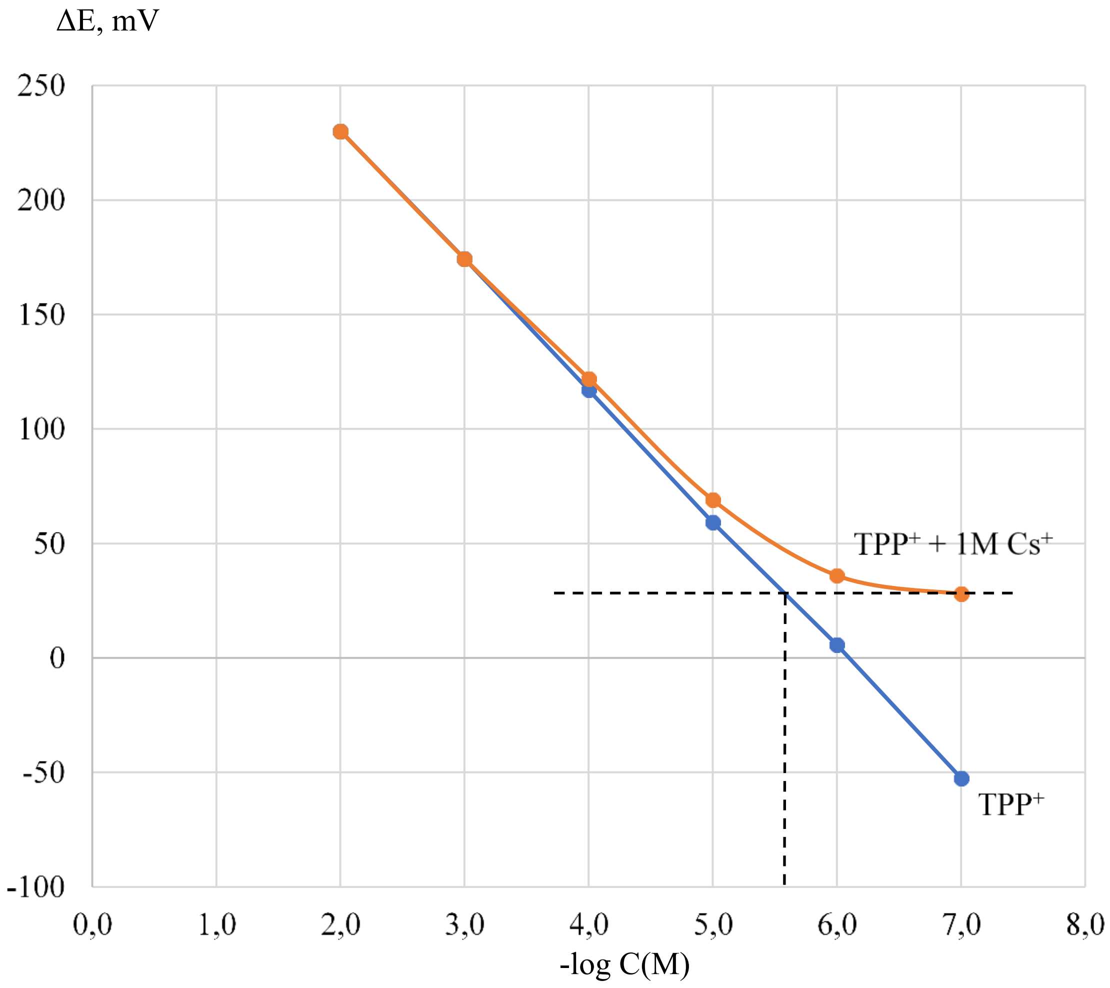

Figure S58. Graphical determination of the selectivity coefficient $pK^{pot}_{TPP^{+}/Cs^{+}}$

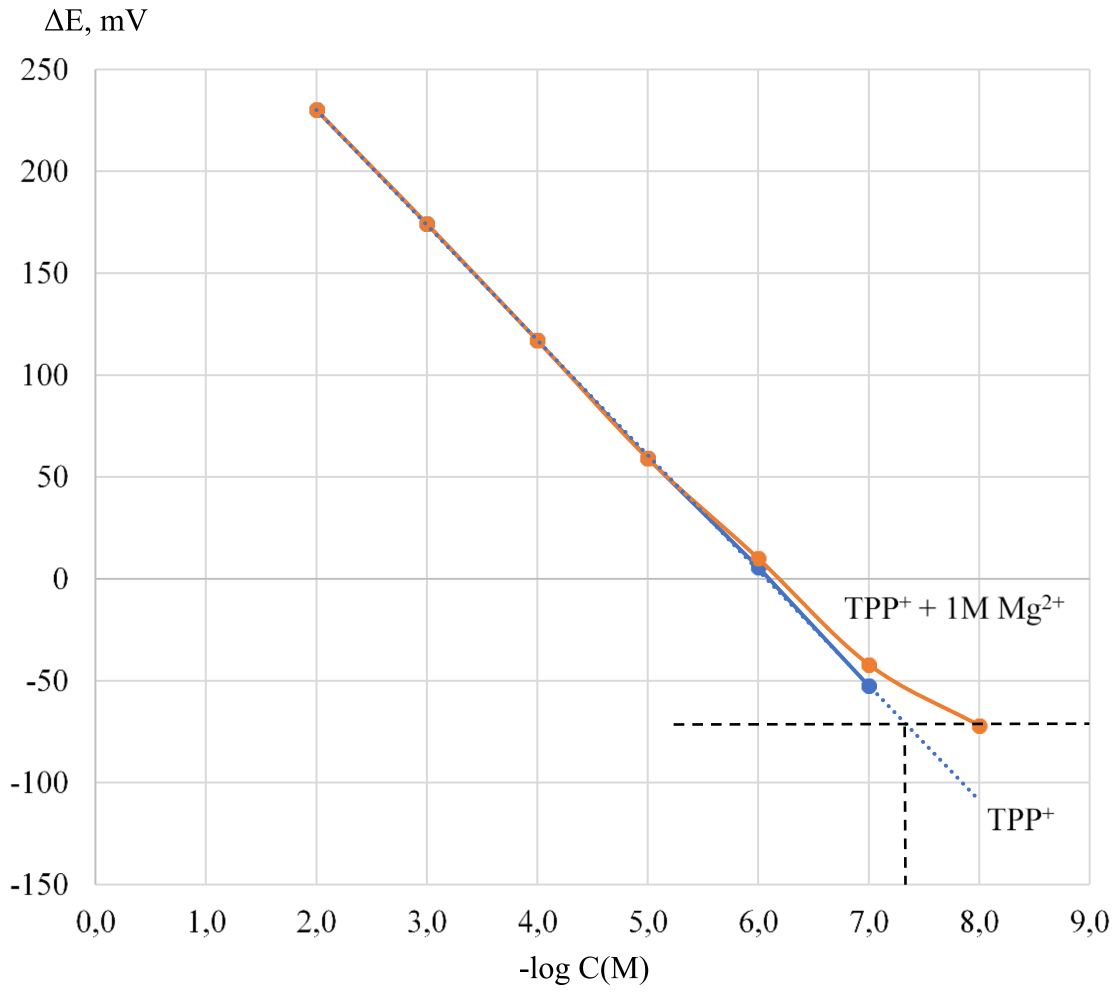

Figure S59. Graphical determination of the selectivity coefficient $pK^{pot}_{TPP^+/Mg^{2+}}$

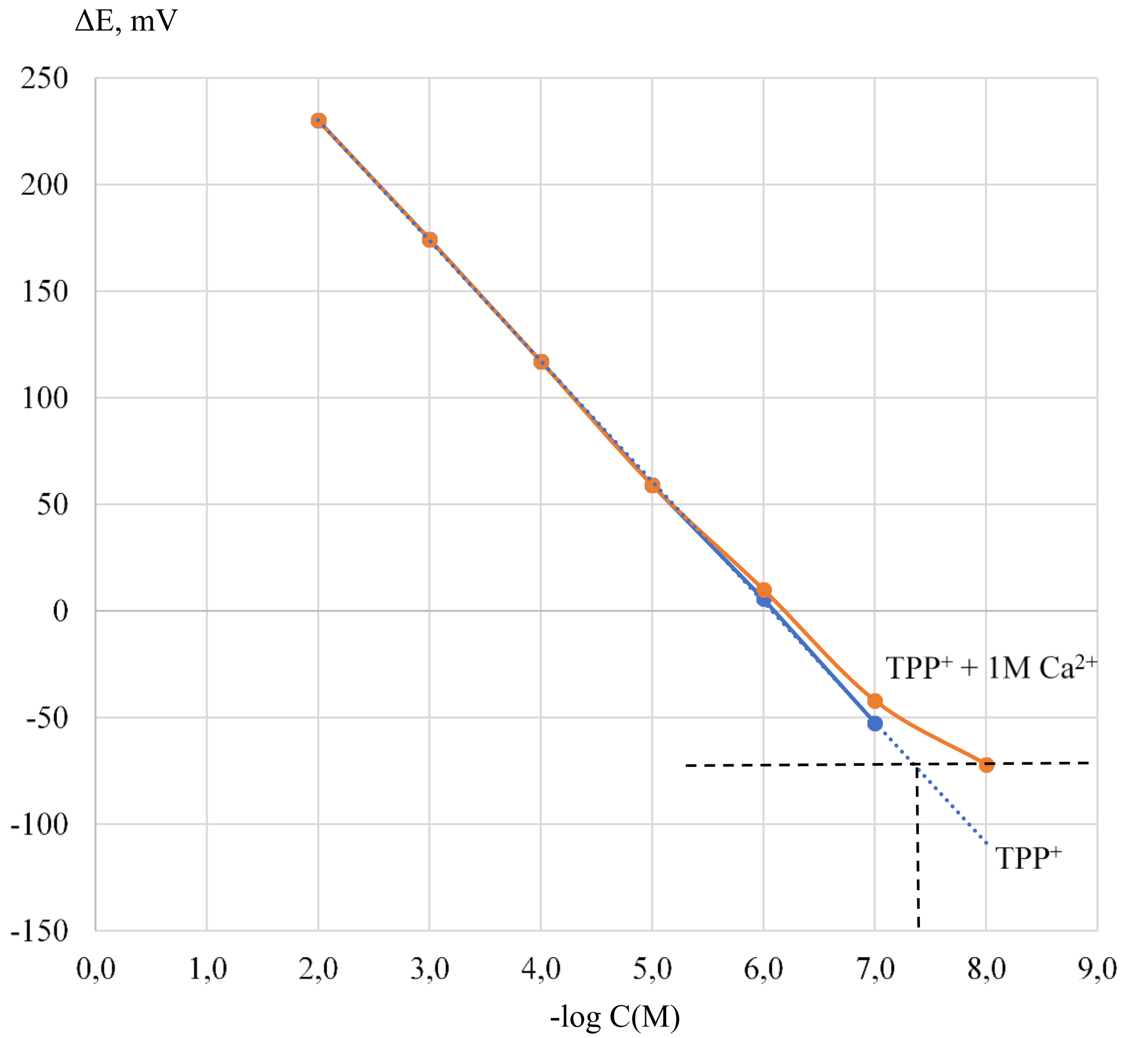

Figure S60. Graphical determination of the selectivity coefficient $pK^{pot}_{TPP^{+}/Ca^{2+}}$

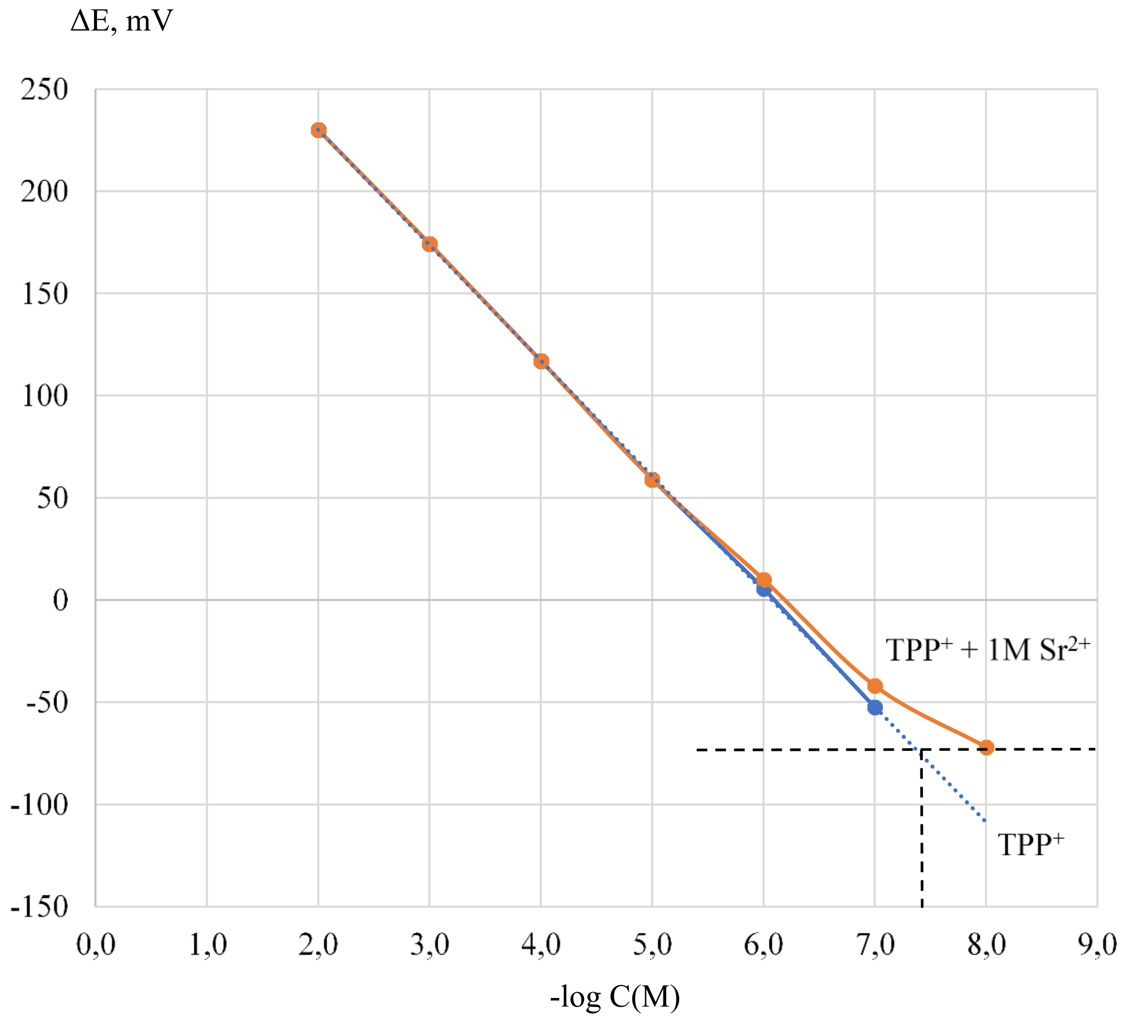

Figure S61. Graphical determination of the selectivity coefficient $pK^{pot}_{TPP^{+}/Sr^{2+}}$

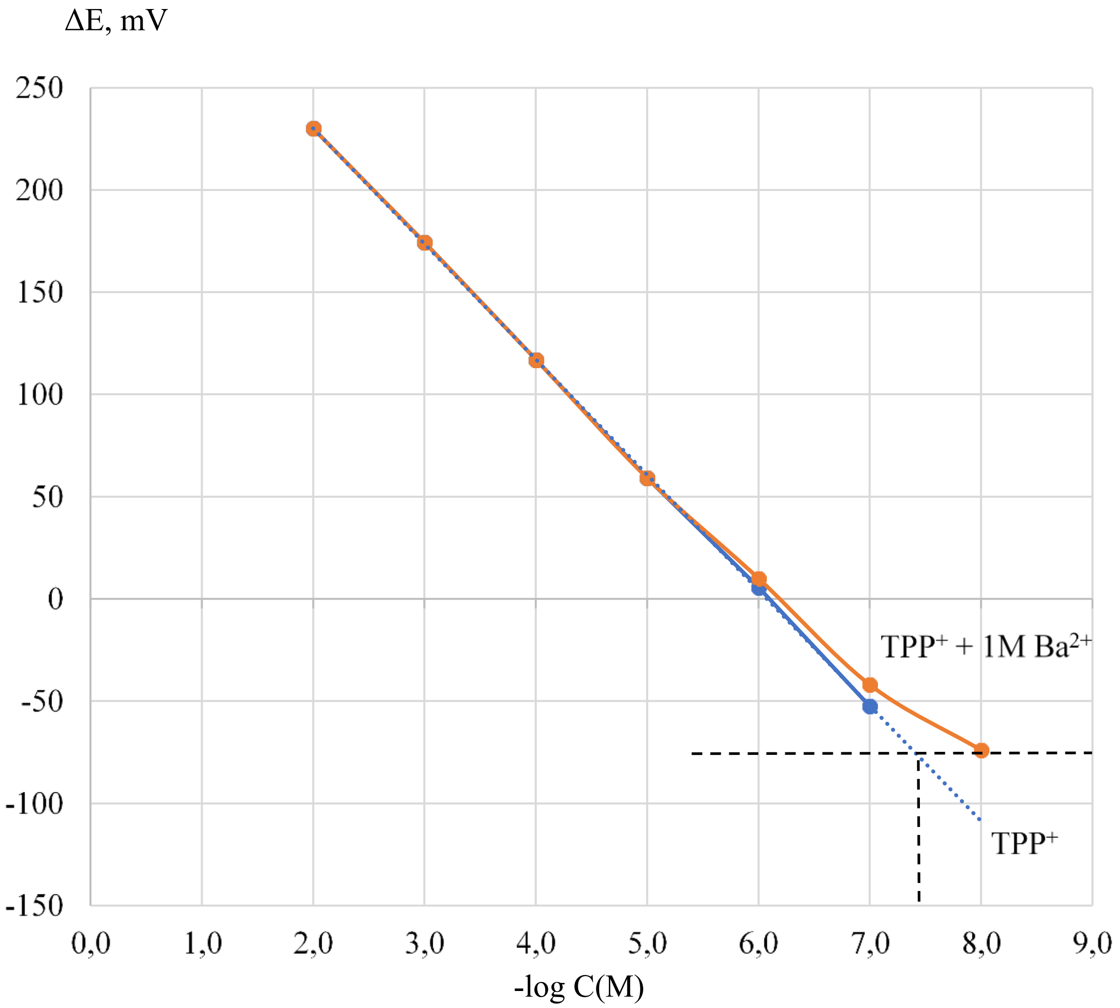

Figure S62. Graphical determination of the selectivity coefficient $pK^{pot}_{TPP^+/Ba^{2+}}$

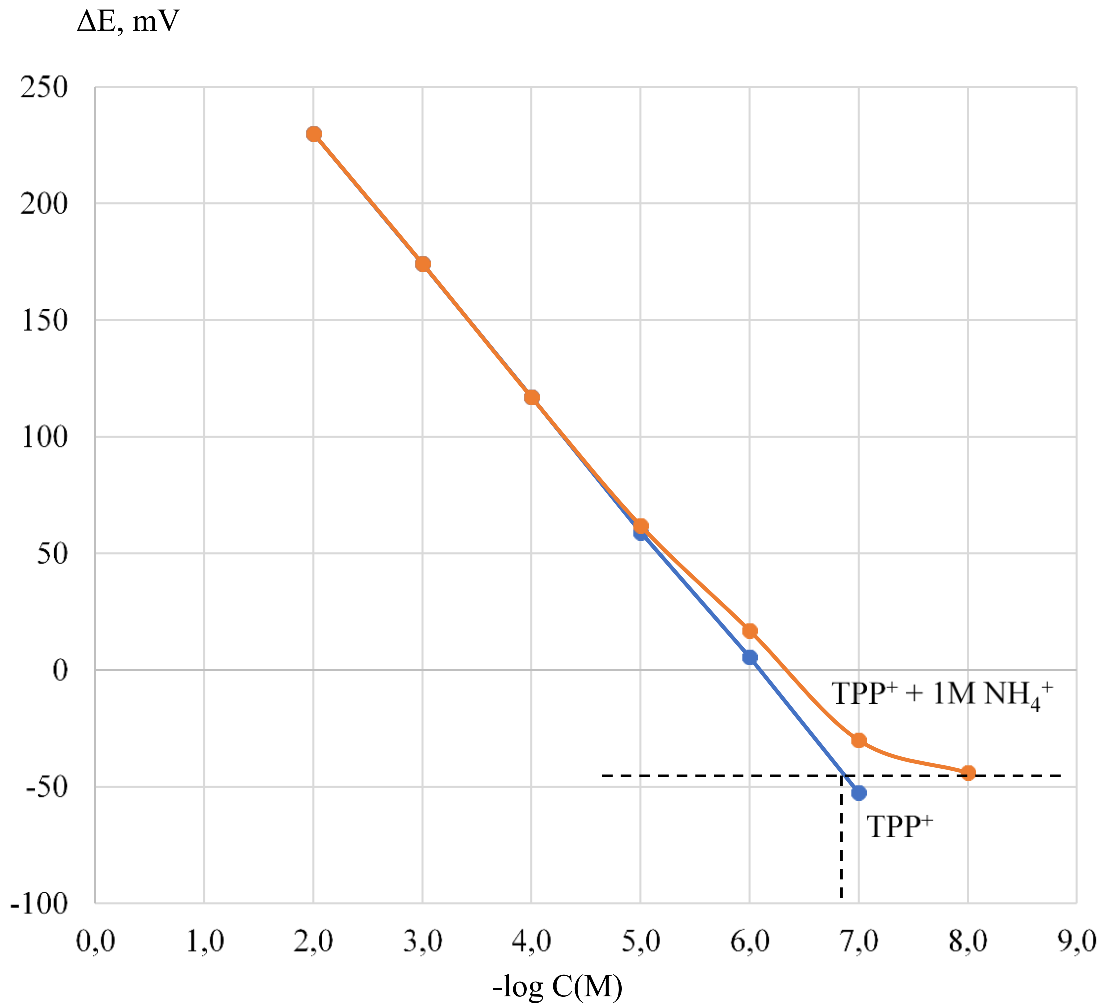

Figure S63. Graphical determination of the selectivity coefficient $pK^{pot}_{TPP^+/NH4^+}$

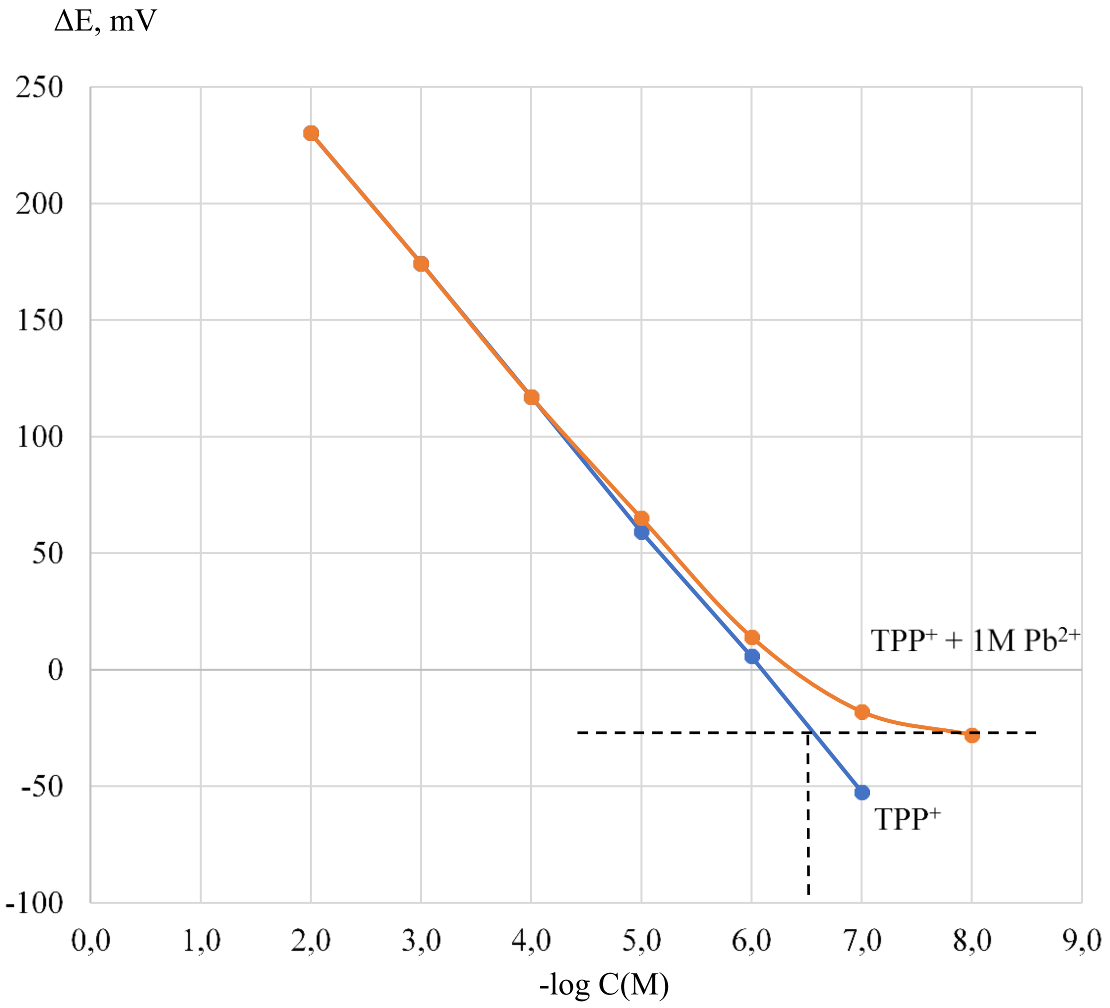

Figure S64. Graphical determination of the selectivity coefficient $pK^{pot}_{TPP^+/Pb^{2+}}$

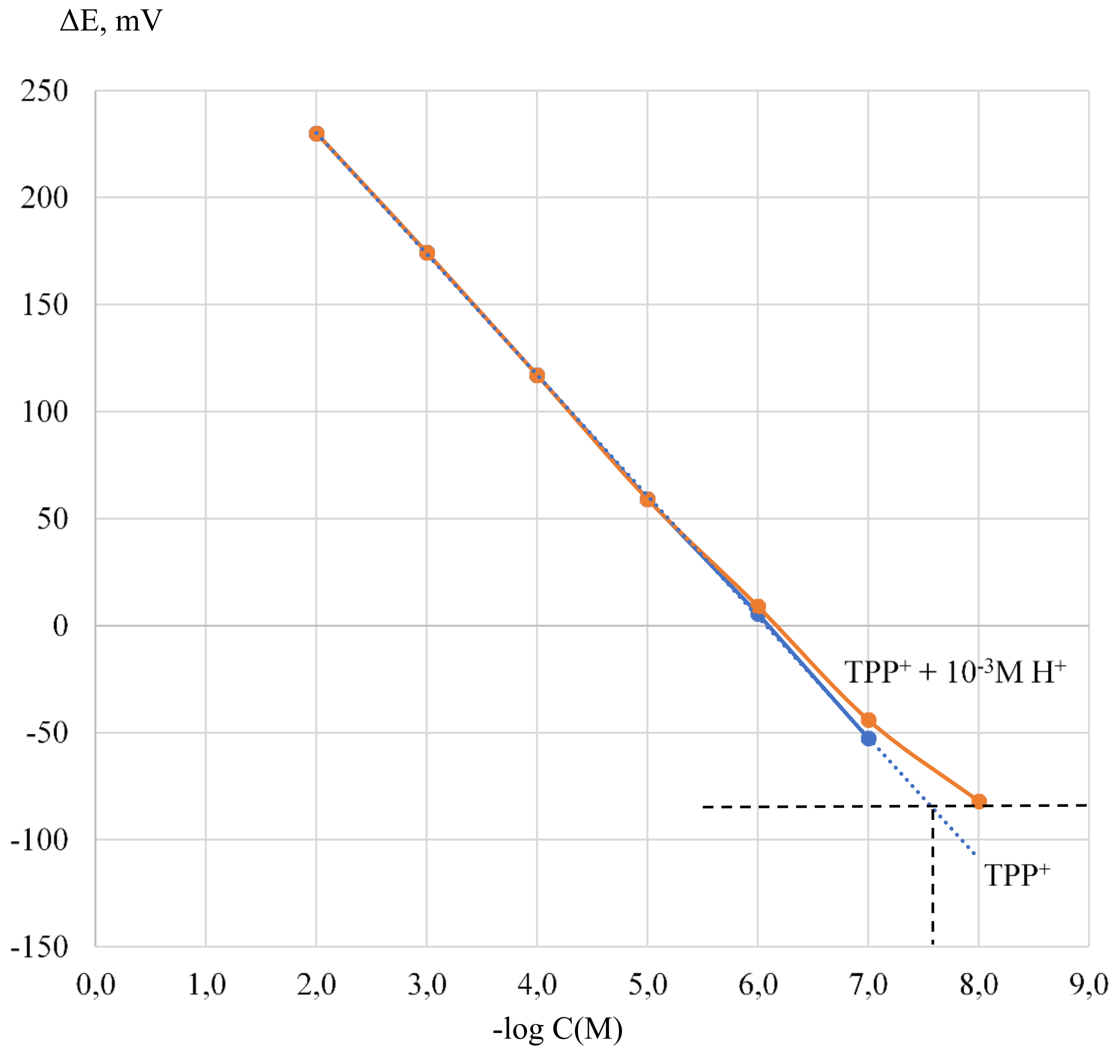

Figure S65. Graphical determination of the selectivity coefficient $pK^{pot}_{TPP^+/H^+}$

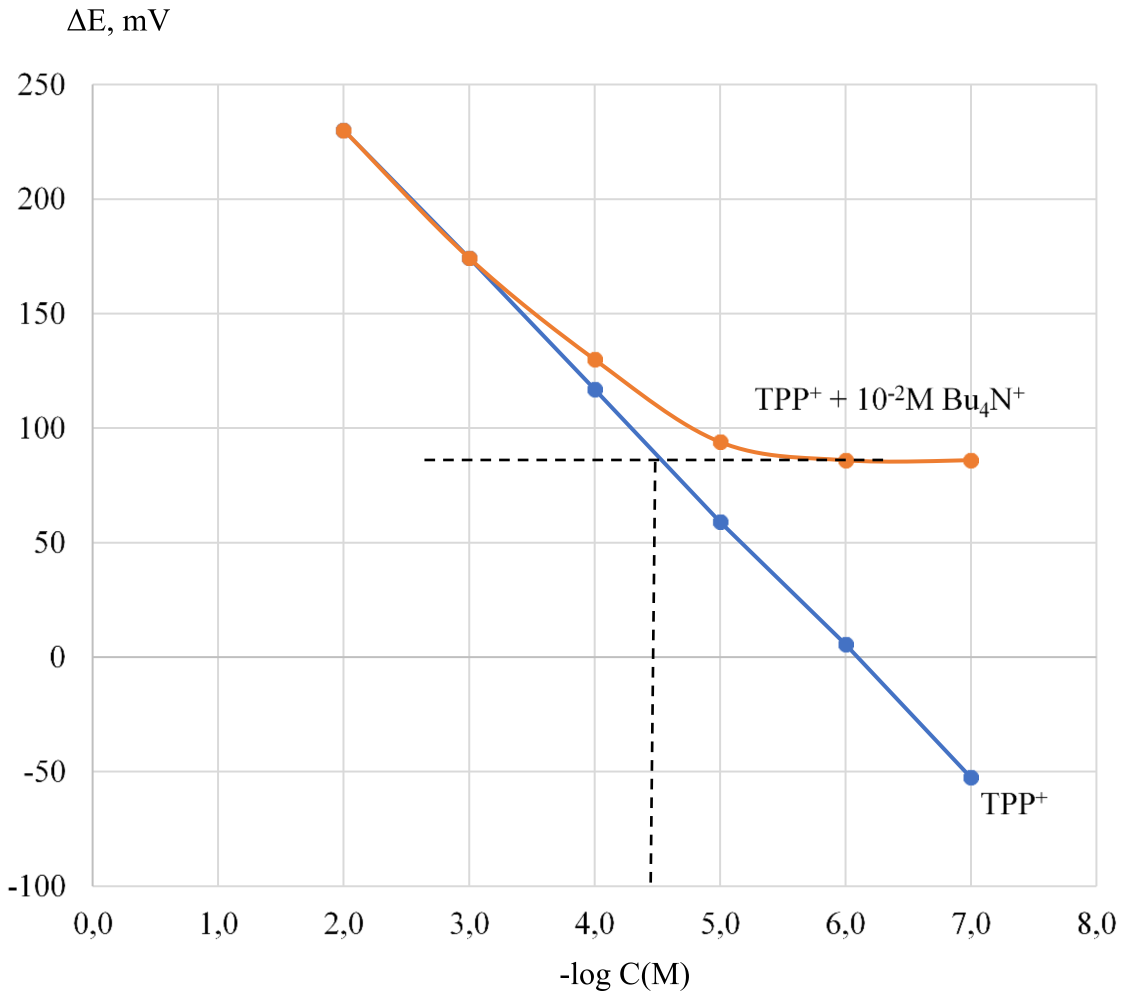

Figure S66. Graphical determination of the selectivity coefficient $pK^{pot}_{TPP^{+}/Bu4N^{+}}$
